# Multi-omics approach to COVID-19: a domain-based literature review

**DOI:** 10.1186/s12967-021-03168-8

**Published:** 2021-12-07

**Authors:** Chiara Montaldo, Francesco Messina, Isabella Abbate, Manuela Antonioli, Veronica Bordoni, Alessandra Aiello, Fabiola Ciccosanti, Francesca Colavita, Chiara Farroni, Saeid Najafi Fard, Emanuela Giombini, Delia Goletti, Giulia Matusali, Gabriella Rozera, Martina Rueca, Alessandra Sacchi, Mauro Piacentini, Chiara Agrati, Gian Maria Fimia, Maria Rosaria Capobianchi, Francesco Nicola Lauria, Giuseppe Ippolito

**Affiliations:** 1 National Institute for Infectious Diseases, “Lazzaro Spallanzani” – IRCCS, Via Portuense, 292, 00149 Rome, Italy; 2 Dept. Biology, University of Rome Tor Vergata, Via della Ricerca scientifica 1, Rome, Italy; 3 Dept. Molecular Medicine, Sapienza University of Rome, 00185 Rome, Italy

**Keywords:** COVID-19, SARS-CoV-2, Omics, Conceptual domain, Pathways, Host signatures, Phenotypes

## Abstract

**Background:**

Omics data, driven by rapid advances in laboratory techniques, have been generated very quickly during the COVID-19 pandemic. Our aim is to use omics data to highlight the involvement of specific pathways, as well as that of cell types and organs, in the pathophysiology of COVID-19, and to highlight their links with clinical phenotypes of SARS-CoV-2 infection.

**Methods:**

The analysis was based on the domain model, where for domain it is intended a conceptual repository, useful to summarize multiple biological pathways involved at different levels. The relevant domains considered in the analysis were: virus, pathways and phenotypes. An interdisciplinary expert working group was defined for each domain, to carry out an independent literature scoping review.

**Results:**

The analysis revealed that dysregulated pathways of innate immune responses, (i.e., complement activation, inflammatory responses, neutrophil activation and degranulation, platelet degranulation) can affect COVID-19 progression and outcomes. These results are consistent with several clinical studies.

**Conclusions:**

Multi-omics approach may help to further investigate unknown aspects of the disease. However, the disease mechanisms are too complex to be explained by a single molecular signature and it is necessary to consider an integrated approach to identify hallmarks of severity.

**Supplementary Information:**

The online version contains supplementary material available at 10.1186/s12967-021-03168-8.

## Background

Advances in molecular and cellular biology in last few decades have triggered tremendous growth in available experimental data, not easy to manage and interpret. Protein–protein interactions (PPI), gene expression, chromatin accessibility and epigenetic modification, signal transduction and metabolic networks are among the categories addressed in systems biology [1]. Mechanistic pathway models can link biological variations, molecular mechanisms and cellular behavior, by coupling molecular interactions in each pathway with their specific endpoint, and contextualizing database query through omics.

To represent pathophysiological mechanisms, the disease map is surely a key emerging concept. It connects bioinformatics, molecular biology and clinical research, with the potential to link conceptual domains of biomedical knowledge with clinical data, providing an intermediate step between conceptual and executable models [2, 3].

A domain can be defined as a conceptual repository, which exhaustively summarizes the variability of a specific biological field and are useful to summarize multiple biological pathways involved at different levels. The conceptual domains mirror the hierarchical structure of the corresponding biological contexts, that can be exemplified by the organization of protein domains. In fact, protein domains have specific functions into each single protein, so that they can be involved in different biological contexts, but on the other hand proteins with different biological functions can have similar domains. They may occur independently or as part of complex multidomain protein architecture with a specific function. Protein domains can therefore be viewed as a 'parts list' for biology. Since protein interactions characterize biological processes that allow the development of different levels of the biological architecture, a macro-domain can be identified as the structural biological level of defined biological processes.

One or multiple macro-domains can be identified for an infectious disease, such as COVID-19, organized on a hierarchical structure. At the upper level, the virus–host interactome based on PPIs could help to understand the disease mechanisms. However, the pathophysiology linking SARS-CoV-2 infection to its clinical phenotypes is still too complex and involves specific pathways, as well as different cell types and multiple organs. A systemic approach and perspective is necessary to untangle this complex network, by collecting mechanistic knowledge scattered across scientific literature and biological databases.

A network-based explorative method of molecular interactions, generally PPIs, can help to understand the disease mechanisms, and the virus–host interactome represents one of its main applications, as it can unravel clusters of molecular interactions, so highlighting the engagement of host pathways induced by the virus [4–6].

The visual exploration of diagrams on COVID-19 Disease Map repository allows to parse such clusters at mechanistic level, pointing out potential molecular interactions to be added to the existing diagrams on the repository (e.g., SARS-CoV-2 E protein interactions) [7, 8].

The aim of the present study is to shed light on the molecular pathophysiology of COVID-19, linking SARS-CoV-2 infection to its clinical phenotypes, and to delineate how different and complex COVID-19 clinical courses are, depending on the involvement of pathways, as well as different cell types (alveolar cells type 1, lymphocytes, neutrophils, platelets, ect) and organs (lungs, blood, colon, liver, etc.). To achieve this goal, we carried out a scoping review of the available literature data, based on conceptual domains.

## Materials and methods

### Conceptual domains identification

We used previous studies from two research groups as methodological reference in order to define the conceptual domains identified for this review.

Particularly, the study by Gordon and colleagues [6] was used to define the concept of host- pathogen interaction, since it represents the first exhaustive experimental work about in vitro molecular interactions between SARS-CoV-2 proteins and human proteins involved in complex biological processes.

In addition, the studies published by Ostaszewski et al. [7, 8] were selected for introducing the concept of Disease Map, since they represent theoretical and hierarchical models of complex biological mechanisms of interaction, describing the involvement of specific pathways, consistent with specific disease phenotypes.

We firstly identified two conceptual macro-domains: (1) networks of virus–human PPIs; (2) interaction clusters of pathways, involving dynamic processes at different times, not represented in a static view.

**Fig. 1:**
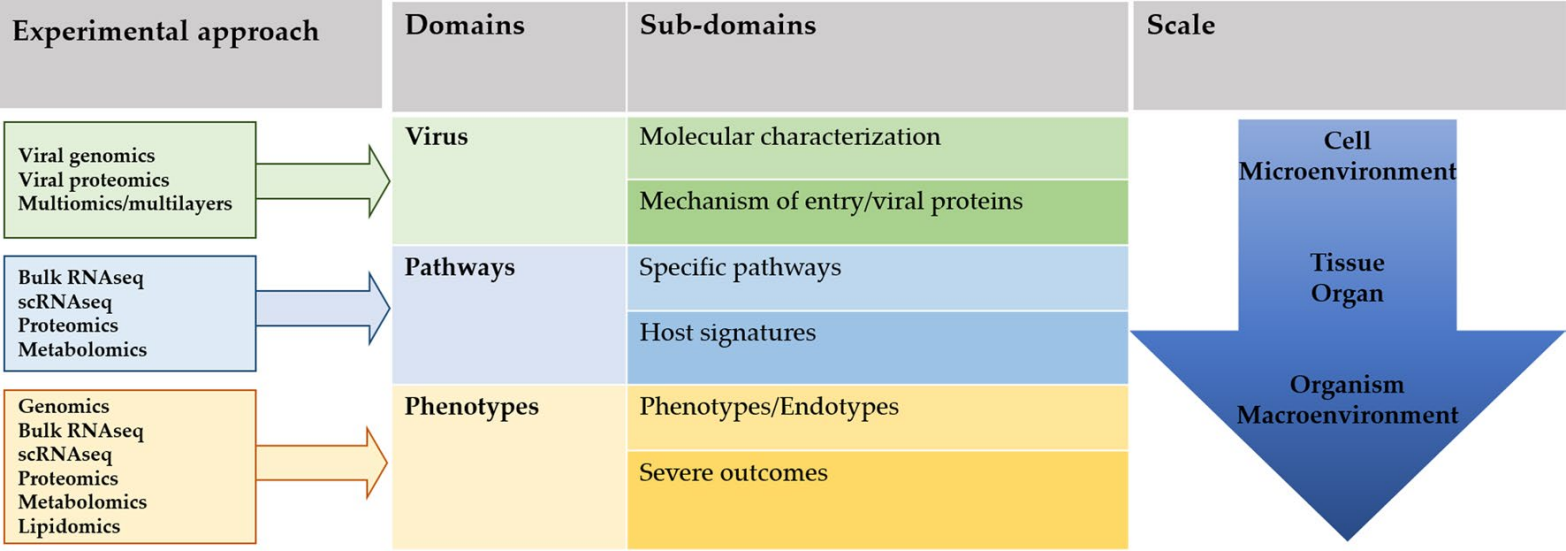
Schematic diagram of conceptual domains, subdomains, and omics data, distributed on scale gradient. The definition of COVID-19 phenotypes is the WHO one [9]

The macro-domains were grouped into three domains divided, in turn, in six sub-domains, as shown in Fig. 1.

### Scoping review

An interdisciplinary team was set up with expert addressing the three domains plus the sub-domain “host signatures”, that presented a conspicuous pertinent literature.

Each working group performed an independent literature review; this was conducted in compliance with international reference guidelines using the scoping review method [10, 11]. For each domain, the scoping review results were processed to identify features of COVID-19 and SARS-CoV-2 infection, compared with SARS-CoV and MERS-CoV infections. In particular, the review was focused on studies that provide omics data correlated to immune response and clinical phenotypes, in order to identify knowledge gaps regarding the link among omics and clinical data, focusing on the different COVID-19 features and the on risks of clinical progression.

**Fig. 2:**
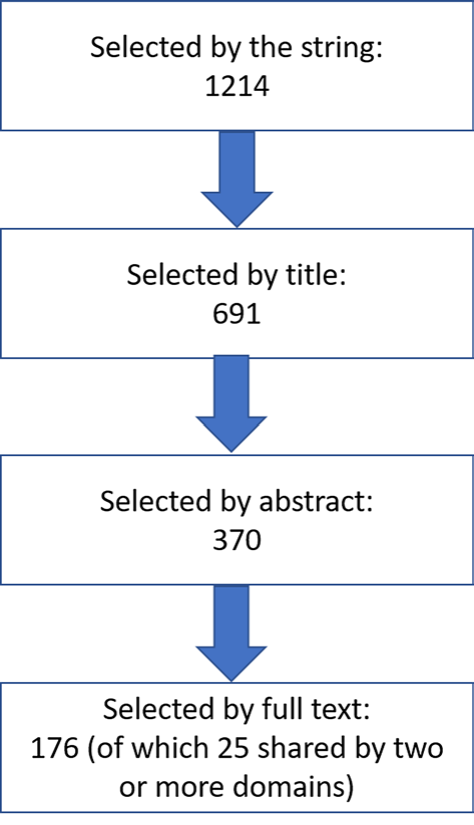
Article selection flowchart

Protocols and tools used for the scoping review are reported in detail in Supplementary text. Articles were selected using the string specified in the Supplementary text on PubMed, in the period between January 2002 and February 2021, with the identification of 1214 articles, after removing duplicates and non-English papers. As shown in Fig. 2, in the subsequent step 691 articles were selected, catalogued in 3 domains: (1) “virus”; (2) “pathways”; (3) “phenotypes”. Each working group evaluated full text article to define at best the possible subdomains, as shown in Fig. 1.

A further selection has been done in three steps, based on title, abstract and full text. This process led to a final selection of 176 articles, of which 25 were shared by two or more domains, providing 151 unique articles, listed in the table of evidences (TE). To univocally identify the mechanisms described for domains “pathways” and “phenotypes”, the Reactome Pathway nomenclature and Reactome Code were used [12].

## Results

### Molecular characterisation of the virus and of the entry phase into the host cells

For each domain, the omics literature was consulted in reference to available data, concerning viral genomics, proteomics and molecular interactions with the host, in light of their possible involvement in the pathogenic mechanisms of the disease, and based on information from other Coronavirus infections. The results of such analysis are summarized in Table 1, in Additional file 1: Tables S3 B and Annex 1, and in Additional file 2: Tables S3 A. In the next paragraphs the results of such analysis are described in more details.

**Table 1:** Mechanisms and current evidence about fields of SARS-CoV-2 characterization and entry reported for specific omics data: a) viral genomics and proteomics; b) host-virus interactions at multi-omics levels

A. SARS-CoV-2 characterization		
Investigation field	Viral genomics evidence	Viral proteomics evidence
Genome evolution and geographical distribution	Evolutionary history of SARS-CoV-2 reconstructed by a phylogenetic approach among the 5 subgenera of Betacoronaviruses [TE01-TE03] At the beginning of pandemic SARS-CoV-2 genomes were classified into 5 main clades: S84, V251, I378, D392, and G61 (the most frequent ancestral type) [TE04-TE05]	
Genomic hotspots for mutation, drivers of evolution and correlation with pathogenesis	In SARS-CoV-2 genomes: 10 hyper-variable genomic hotspots [TE14] Genomic regions encoding nsps, except nsp11, had values of dN/dS ratio < 1. Among the structural genes, only S and M displayed dN/dS < 1. Deletions in *ORF7b* and *ORF8* of SARS-CoV-2 genome confer lower odds of developing hypoxia in infected hosts [TE09; TE12]	
Intra-host genomic variability	Small- and large-scale intra-host variations [TE19-TE20] Spatial–temporal redistribution of variants in respiratory and gastro-intestinal tract [TE19-TE21]	
Single viral proteins		Two mutations in nsp6 and in a region near *ORF10* confer lower stability to S, N, M, E proteins, linked to autophagy. [TE24-TE25] Non-conservative substitutions in functional regions of the S, nsp1 and nsp3 may contribute to separate SARS-CoV and SARS-CoV-2 in spread and virulence [TE27]
Whole viral proteome		Dynamicome study, based on Viral Integrated Structural Dynamic Database (VIStEDD), among 273 virus/host PP interactions highlighted 6 major viral nodes influencing the activity of 166 host nodes involved in various cellular processes [TE28-TE29]
Immune proteomics		Viral proteomics was used to design multi-epitope vaccines and to find possible host–pathogen molecular mimicry [TE31]

B. SARS-CoV-2—host interactome	
Investigation field	Multi-omics
Viral RNA and protein interactions	9 potential silencer RNA (siRNA) targets, conserved among all the studied SARS-CoV-2 genomes. [TE32] 3 SARS-CoV small viral (sV) RNAs involved in lung pathology of mice. [TE33] In Vero E6 and to Huh-7 cells infected by SARS-CoV-2, 163 and 229 host protein bind SARS-CoV-2 RNA. [TE37] GO enrichment analysis revealed that most of the proteins were protective from virus-induced cell death, regulating SARS-CoV-2 pathogenicity. [TE38] Functional analysis discovered novel proviral genes and pathways, including chromatin remodelling complexes [TE33-TE39]
Virus–host protein–protein interactions	1311 PPIs were used to build a large coronavirus-host interactome. Relevant small protein complexes: EIF4E2-GIGYF2 dimer, involved in proteins translation repression and the MAT2A-MAT2B complex [TE42]; DNA-PK kinase contributing to interferon induction [TE42]; Mitochondrial proteins PHB, PHB2 and STOML2, regulating mitophagy. [TE42] Host interactome linked to S of SARS-CoV and MERS-CoV: innate immunity involved. [TE43] The International Molecular Exchange (IMEx) Consortium cured a dataset of PPI, contained interactions of SARS-CoV-2 and SARS-CoV, with human proteins [TE44]
Multilayer virus–host interactions	Multilayer analysis, in few cases may predict different SARS-CoV-2 disease phenotypes: Immune regulation appears to be linked to gene *TMPRSS2*, involved in SARS-CoV-2 virus entry [TE52] SARS-CoV-2 transcripts detectable only in BAL from severe COVID-19 patients. [TE53] SARS-CoV-2 transcripts strongly enriched in ciliated and epithelial progenitor cell population and in the SPP1 + macrophage population. [TE53] Master Regulator Analysis on multiple datasets showed that SARS-CoV-2 mainly affected: Apoptotic and mitochondrial mechanisms [TE49] ACE2 protein receptor regulation [TE49] COVID-19 Disease Map, an open-access repository containing ordered molecular interaction diagrams, implicated in the disease. It is available on website [TE50]
Virus–host receptor interaction	Interactome of 45 proteins connected to four cell surface seed proteins (ATP6V1A, AP3B1, STOM, and ZDHHC5) with physical affinity to viral S,E and M proteins. [TE73] 7 miRNA (miR-124-3p, let-7 g-5p, miR-133a-3p, miR-133b, miR-218-5p, miR-22-3p, and miR-506-3p) interconnect with proteins involved in viral entry and replication process. [TE73] A probabilistic modelling using iDREM (interactive Dynamic Regulatory Events Miner) revealed: 63 significant regulators expressed in SARS-CoV-2 infected Calu-3 cells (14 also identified analysing the transcriptome of PBMC and Broncho-alveolar cells) An interactome involved in viral entry, including prohibition (PHB) as alternative receptor or co-receptor [TE48]

#### Genome evolution and geographical distribution

In this field of investigation, the evolutionary history of SARS-CoV-2 was reconstructed, starting from phylogenetic comparison with related Betacoronaviruses [TE1-TE5].

Global Initiative on Sharing Avian Influenza Data (GISAID) classification of SARS-CoV-2 clades was reported together with their spread (Last up-dated on November 10th 2020, based on 175,000 genomes).

#### Genomic hotspots for mutation, drivers of evolution and correlation with COVID-19 pathogenesis

In SARS-CoV-2 genome, ten hyper-variable hotspots were identified. In the *S* gene, some regions presented signs of positive selection, i.e. dN/dS > 1, particularly within the receptor binding domain (RBD) in the S1 subunit, in the FURIN cleavage site and in the segment encoding the S2 and S2’ subunits. Also *ORF3a*, *E*, *ORF6*, *ORF7a*, *ORF8*, *N*, and *ORF10* presented dN/dS > 1, indicating positive selection pressure. People infected with *ORF7b* and *ORF8* SARS-CoV-2 deleted variants had a lower odd of developing hypoxia.

Integrated comparative genomics and machine learning techniques identified 11 regions in SARS-CoV-2 genome, reliably predictive of high fatality rate [TE6-TE18].

#### Intra-host genomic variability

In SARS-CoV-2 infected host, the virus displayed small scale intra-host variation, while spatial–temporal redistribution of SARS-CoV-2 “quasispecies” in respiratory and gastro-intestinal tracts in human hosts was observed, with a higher significantly genetic diversity observed in gastrointestinal compared to respiratory tract samples [TE19-TE22].

#### Single viral protein and whole viral proteome studies

This investigation field concerned omics studies about both single proteins and whole SARS-CoV-2 proteome characterization. Some of these studies were in silico and organized in a modular hierarchical scale of virus/host PPIs, allowing to build a dynamic and integrated structure, named “SARS-CoV-2 dynamicome” [TE24-TE29].

#### Immune proteomics

The host–pathogen molecular mimicry was investigated on the basis of viral proteomics, also used in studies aimed at developing innovative anti-COVID-19 vaccines [TE30-TE32].

#### Viral RNA and host protein interactions

In silico studies predicted possible SARS-CoV-2 RNA regions of interaction with host proteins.

Experimental studies provided description of the SARS-CoV-2 RNA–protein interactome in different SARS-CoV-2 in vitro infected cell lines. Other works provided functional interrogation of the host proteins involved in such interactions, revealing that most of them regulated virus entry into host cells, protected the host from virus-induced cell death or were involved in SARS-CoV-2 pathogenicity [TE33-TE39, TE45, TE51]. Besides interaction with host proteins, some studies also identified SARS-CoV-2 genomic regions to be potential silencer RNA (siRNA) targets, or could interact with host microRNAs (miRNAs) [TE32].

#### Virus-host protein–protein interactions (PPIs)

Several research studies addressed PPIs between single betacoronaviruses and host proteins or further detailed, by in silico analysis, already described interactomes [T40-TE44].

#### Multilayer analysis of virus-host interactions (transcriptomics, proteomics)

By integrating viral-host transcriptomics and proteomics in a multilayer analysis it was possible to characterize COVID-19 phenotypes [TE45]. Omics studies, particularly relevant for pathogenesis investigations, were based on ex-vivo studies, with clinical samples derived from SARS-CoV-2 infected subjects displaying different clinical phenotypes. In fact, some of these investigations were performed by multi-omics network-biology-fueled approach to provide the principal host components affected by SARS-CoV-2 infection. They pose the basis for the constructions of a COVID 19 Disease Map. Additional file 1: Table S2 A provides a more detailed description of the above reported information [TE48, TE49, TE50, TE52, TE53].

#### Viral entry

*Expression of host entry factors in human tissues* Expression of ACE2 and TMPRSS2 in human tissues was addressed by omics approaches in several studies [TE55, TE56, TE57, TE59, TE60, TE63]. Importantly, very low or absent ACE2 expression was reported in organs/tissues considered as the main target for SARS-CoV-2 replication, including lung, bronchus, and nasal mucosa, suggesting a dynamic regulation of entry factors upon infection and a role for possible alternative receptors.

*SARS-CoV-2 interaction with entry factors* Two studies explored the cross-talk between SARS-CoV-2 and host proteins during the entry and subsequent steps of viral replication. In the first paper proteins on the host cell membrane (ATP6V1A, AP3B1, STOM, and ZDHHC5) were identified that may enable binding to SARS-CoV-2 structural proteins. On other hand, several miRNAs were also identified to inhibit proteins involved in viral entry [TE73]. The second study proposed three interactomes from probabilistic modelling, using iDREM (interactive Dynamic Regulatory Events Miner): the first one, involved in creating a suitable environment for the virus, includes ATP6V1A; the second one includes PHB as alternative receptor or co-receptor; the third one, involved in sustaining viral replication, includes oxidative stress and inflammation proteins [TE48].

Regarding the viral variants of concern (VOC), B.1.351, P1 and some B.1.1.7 strains harbor, among others, mutations potentially important for pathogenesis, such as K417T/N, E484K, and N501Y. These substitutions seem to alter the interaction of S glycoprotein with ACE2 receptor, leading to increased transmissibility compared to the previous circulating strains, and especially for B.1.351 and P1 lineages, leading to reduced susceptibility to neutralizing antibodies elicited by non-variant strains and by current vaccines. In fact, viral evolution studies show that the RBD of S glycoprotein is highly variable and that immune escape mutations (i.e., E484K) may emerge independently, in multiple lineages, spreading worldwide, and leading to the further accumulation of additional changes, that may increase the risk of significant reduction of host immunity and/or of monoclonal antibody therapy, as well as of vaccine efficacy [TE69, TE70, TE71, TE72].

### Pathways

To hierarchically evaluate cellular processes involved in SARS-CoV-2 infection, we assigned a univocal Reactome Code to each cellular mechanism reported in the scanned literature for the pathway analysis. Of note, this approach was performed for proteomics, transcriptomics and bioinformatics studies in vitro, where the study model was represented by cell lines infected with SARS-CoV-2 (Table 2 and in Additional file 1: Tables S4 and Annex 2). The occurrence of each mechanism was organized on the basis of Reactome Pathways (Additional file 3: Table S6).

**Table 2:** Immune response to SARS-CoV-2 infection in lung and other tissues (A), peripheral blood (B) and specific cell types among blood immune cells (C). Pathways, host signature and body districts, subset per specific omics data: host proteomics, bulk and single cell RNAseq (scRNAseq)

A. Lung and other tissues			
Pathway	Omics	Body district(s)	Host signature
Inflammatory cytokines	Proteomics	Lung	DEP* up in fatal COVID-19 [TE96, TE110]
IFN response IL6 signaling Complement cascade	BULK RNAseq	Nasopharyngeal (NP) swabs BAL	DEG** up in COVID-19[TE99, TE108]
Monocyte and neutrophil recruitment	BULK RNAseq	Nasopharyngeal (NP) swabs BAL	DEG** up and down in COVID-19[TE99, TE108]
Morphogenesis and migration of immune cells	BULK RNAseq	BAL	DEG** down in COVID-19[TE108]
Neutrophils extracellular traps TGF-beta response Extracellular traps	BULK RNAseq	Nasopharyngeal (NP) swabs Lung colon	DEG** up in fatal cases and correlated with SARS-CoV-2 viral load in NP[TE119]
Anti-inflammatory pathways	scRNAseq/CyTOFF	BAL	DEG** down in CD14 + /CD16 + cells of severe cases[TE99]
Immune cell activation	scRNAseq/CyTOFF	Colon	DEG** down in fatal COVID-19 cases[TE110]

B. Peripheral blood			
Pathways	Omics		
	Proteomics	Bulk RNAseq	Metabolomics
Inflammation and immune response Neutrophil activation Acute phase response Platelet degranulation Antimicrobial response Tissue damage Coagulation and complement activation	Soluble mediators DEP* up in COVID-19 [TE87,TE88,TE89]		
Platelet degranulation Coagulation and complement activation	Soluble mediators DEP* down in COVID-19 [TE89,TE94, TE95]		
Inflammation and immune response Neutrophil activation Acute phase response Tissue damage	Soluble mediators DEP* up in severe COVID-19 vs mild [TE89, TE90, TE94];		
Inflammation and immune response Lipid metabolism Coagulation Tissue damage	Soluble mediators DEP* down in severe COVID-19 vs mild [TE89, TE90, TE94];		
Acute phase response Coagulation and complement activation Inflammation and immune response	Soluble mediators DEP* up in severe COVID-19 vs mild DEP* up in fatal COVID-19 DEP* up in poor prognosis for COVID-19 DEP* up in COVID-19 vs influenza [TE88, TE96];		
Inflammatory cytokines and chemokines IFN response		DEG* up and down in COVID-19 [TE90]	
Inflammation Immune response Lipid hormones Lipid metabolism Tissue damage			Metabolites up and down in COVID-19 [TE129]
Amino acid metabolism Inflammation and Immune response Lipid metabolism Oxidative Pathways of Cellular Energy Production REDOX homeostasis Tissue damage Urea cycle Xenobiotic metabolism			Metabolites up and down in severe COVID-19 vs. mild [TE101, TE102, TE103, TE104]
Inflammatory response Neutrophil activation generation of NETs Negative regulators of innate immune signaling TCR signaling		DEG** up in severe COVID-19 vs. mild [TE97]	
Negative regulators of innate immune signaling TCR signaling		DEG** down in severe COVID-19 vs. mild [TE89]	
IFN response		DEG** up in mild COVID-19 [TE89]	
Inflammatory response		DEG** up in fatal COVID-19 vs. mild [TE89]	
IFN response IL1 IL6, IL10 signaling Complement/coagulation cascade		DEG** up and down in moderate COVID-19 vs coronaviruses, influenza or bacterial pneumonia [TE98]	

C. Specific cell types among blood immune cells (CyTOF/scRNAseq)			
Innate cell compartment		Adaptive cell compartment	
Pathways	Modulation	Pathways	Modulation
Dysregulation of innate cells	DEG** up and down in COVID-19 [TE96, TE112]	Gene expression related to T cell apoptosis T cells activation, Th1, Th2 and Th17	DEG** up and down in COVID-19 [TE89, TE110, TE112, TE144]
Classical and non-classical Monocytes activation and recruitment Suppressive low density neutrophils NETs formation Dendritic cells function	DEG** up and down in severe COVID-19 vs. mild [TE89, TE105, TE112, TE113]	Negative T cell signaling T cell activation Antibodies production T cell exhaustion Cytotoxic effector cells CD8 polyfunctionality	DEG** up and down in mild, moderate, survivor COVID-19, severe vs mild, survivors vs fatal, COVID-19 vs seasonal coronavirus, influenza or bacterial pneumonia). [TE98, TE105, TE111]

*Differentially Expressed Proteins (up, down) detailed in Additional file 1: Table S4

** Differentially Expressed Genes (up, down) detailed in Additional file 1: Table S4

This approach allowed us to highlight several mechanisms altered by SARS-CoV-2 infection, i.e. signal transduction (R-HSA-162582), translation (R-HSA-72766), post-translation protein modifications (R-HSA-597592), immune response (R-HSA-168256), cell cycle (R-HSA-1640170), apoptosis (R-HSA-109581), autophagy (R-HSA-9612973), lipid metabolism (R-HSA-556833) and vesicle-mediated transport (R-HSA-5653656).

**Fig. 3:**
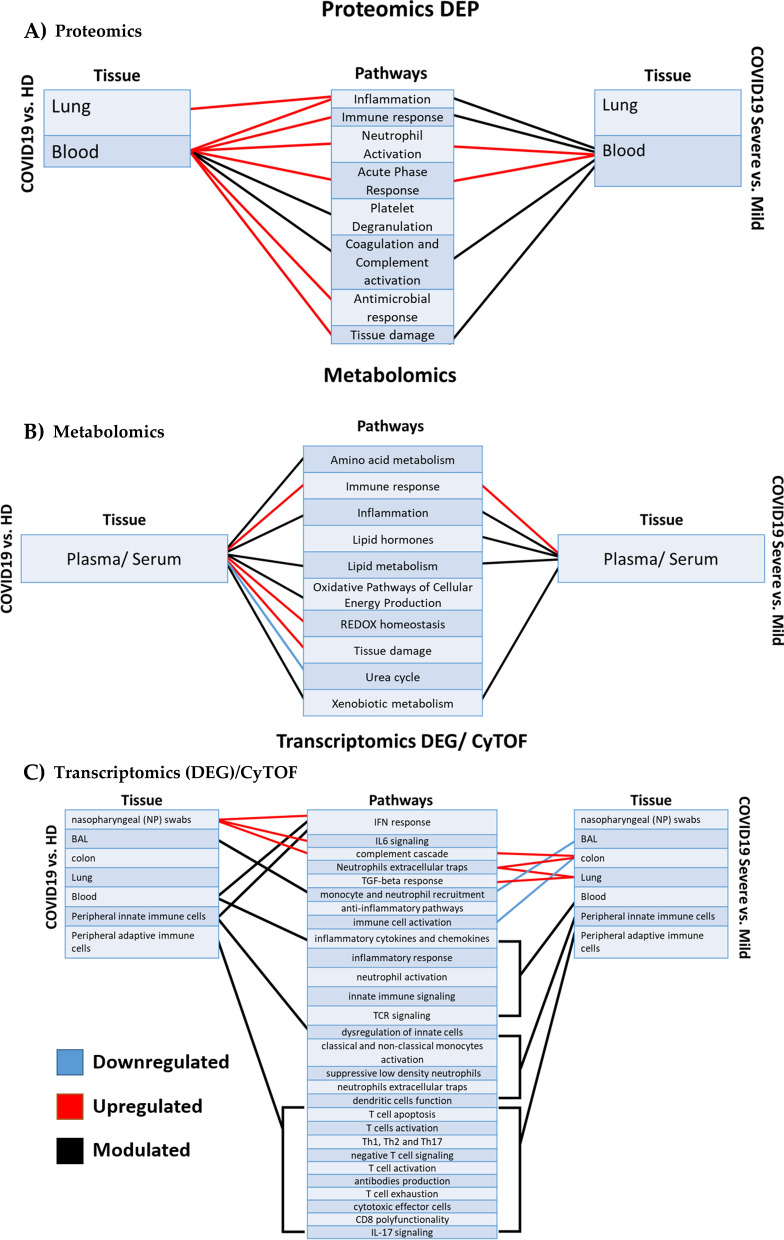
Omics contribution in understanding COVID-19 pathogenesis. Omics data analyzed, organized by omics technique, tissue and pathways comparing COVID-19 to healthy donors or severe versus mild outcomes. Red lines represent upregulated pathways, blue lines represent downregulated processes. A black line is used when the same pathway has been described as both up- and down-regulated

 Figure 3 shows how omics data, organized by omics technique and tissue (Proteomics, Metabolomics and Transcriptomics/CyTOF, in A, B and C respectively), contribute to highlight pathways up- or down-regulated in COVID-19 patients compared to healthy subjects, or in severe COVID-19 patients compared to mild patients.

With respect to signal transduction, mTOR pathway is strongly affected during SARS-CoV-2 infection, leading to the activation of PI3K/AKT and TNF cascades [13] [TE74, TE75, TE76, TE77, TE78]. Moreover, considering that the production of viral proteins depends on host cap-dependent translation [14], this mechanism is also altered by SARS-CoV-2 infection. While on one hand the virus usurps cellular translation machinery to promote its own reproduction, on the other hand cells attempt to reduce translation to contrast the infection. It has been extensively reported that several SARS-CoV-2 proteins bind E3-ubiquitin ligases, usurping cellular degradation machinery, thus promoting virus replication [5, 6] [TE40, TE41,TE49, TE80, TE83]. Other pathways extensively addressed in COVID-19 studies are represented by both innate and adaptive immunity. Specifically, IFN, TNF and NF-kB pathways, cytokine SPP1, GRN, the receptor tyrosine kinase AXL [TE85], NLR and RIG-I signaling are strongly altered by SARS-CoV-2 [TE76], contributing to severe forms of COVID-19 [6]. Similarly, cell cycle is affected by SARS-CoV-2 infection with a rapid reshape of several host mechanisms, leading to cell cycle arrest. However, it is important to consider that most of the reported studies have been performed in cell cultures in vitro, which represent an undisputed model for infection; nevertheless, cell cycle is intrinsically altered in the in vitro culture systems, introducing a bias in COVID-19 model conceptualization. Several network analyses of cellular pathways related to SARS-CoV-2 infection both in vitro [TE44, TE74, TE77, TE78, TE80] and in silico [TE73], show that cell death, in particular apoptosis, is affected. In addition, Stuckalov and colleagues observed an accumulation of several autophagic proteins (e.g. SQSTM1, GABARAPL2, NBR1, CALCOCO2, MAP1LC3B, TAX1BP1) following ORF3 expression, also observed in cells infected by the virus (SQSTM1, MAP1LC3B), suggesting an inhibition of the autophagic pathway during SARS-CoV-2 infection [TE81]. The metabolism of lipids is also affected, regarding the host protein SIGMAR1 involved in lipid metabolism and ER stress response proteins involved in binding nsp6 and ORF9c of SARS-CoV-2 [6] [TE77, TE83]. Finally, it is emerging that different coronaviruses hijack specific RAB GTPases during the infectious cycle. In fact, host RAB2A and RAB7A are critical for HCoV-229E, HCoV-OC43 and HCoV-NL63 infection, while RAB10 and RAB14 play an important role in SARS-CoV-2 infection [TE45].

### Host signatures

The host signature was addressed in studies evaluating the systemic profile of soluble mediators by proteomics and metabolomics approaches. Cellular immune response to SARS-CoV-2 infection was evaluated by transcriptomics, proteomics, and high dimensional single cell analysis (mass cytometry, CyTOF) in peripheral blood and tissues, reported in Table 2, and detailed in Additional file 1: Tables S4 and Annex 3.

#### Systemic profile of soluble mediators

##### Proteomic studies

During the early immune response, the activation of type I/III IFN response represents a key local innate immune player and is associated with chemokines [TE87] and proinflammatory mediators [TE88], leading up to a massive release of inflammatory mediators, called “cytokine storm”. In particular, mild COVID-19 is characterized by increase of IFN-α, IFN-γ, IL6, IL10, IL1RA [TE87, TE88, TE89], while severe disease is characterized by a downregulation of IFNα, and a parallel up-regulation of IFN-β, IFN-γ, IL6, TNF, IL10, CCL7 [TE89, TE90, TE94]. Finally, critical disease is characterized by the upregulation of IL6, TNFs, IL10, RIPK3 [TE89]. Several proteins linked to IL6-mediated proinflammatory cytokine signaling are strongly expressed in severe COVID-19 (Additional file 1: Annex 3A.1). The acute phase proteins (APPs) are an additional class of mediators involved in the early phase immune response in COVID-19. These proteins are up-regulated in the severe forms of COVID-19, and can induce inflammatory cytokines, influence lipid metabolism, and induce neutrophil activation, as shown for S100A8 and S100A9 [15], thus possibly contributing to amplify the cytokine storm. Moreover, APPs can also modulate platelet aggregation and activation of coagulation cascade [16, 17] which are closely related with the severity of COVID-19. Differently, in early COVID-19 infection, down-regulation of complement and coagulation cascades has been observed (C1R, C7) compared to influenza infection [TE88]. (Additional file 1: Annex 3A.2).

Several enzymes with antimicrobial activity are increased in COVID-19 patients’ sera, including CST3, DEFA1, and LYZC, indicating a possible secondary bacterial infection [TE97].

#### Metabolomic studies

Levels of most lipid-related molecules are altered in moderate and severe COVID-19 patients with a clear preference toward their downregulation. Main observed alterations are related to lipoproteins, cholesterol, triglycerides, glycerophospholipids and sphingolipids. Plasma lipid perturbations in COVID-19 patients are consistent with alterations in liver lipoprotein metabolism and changes in circulating exosome contents (Additional file 1: Annex 3A.3).

Metabolites of the tricarboxylic acid cycle (TCA) and β-oxidation are reduced in COVID-19, particularly in severe patients, whereas metabolic intermediates of the glycolysis and pentose phosphate pathways are increased [TE90, TE96, TE100, TE101, TE102, TE103]. This reduction may be the consequence of declined lung functions and blood oxygen level decrease, but may also mirror a response to nutritional changes, especially in severe patients (Additional file 1: Annex 3A.4).

In the serum of COVID-19 patients, amino acids and their derivatives result significantly decreased, especially the gluconeogenic and sulphur-containing ones [TE101, TE102, TE103, TE104]. Moreover, compounds of arginine metabolism, including urea cycle metabolic intermediates and arginine derivatives, dropped down in the serum of COVID-19 patients [TE103, TE104]. The observed changes in amino acid levels could be indicative of liver dysfunction. In addition, reduced circulating tryptophan levels are observed in COVID-19 patients, associated to elevated kynurenine levels [TE94, TE104, TE146]. Kynurenine/tryptophan levels are a general measure of indole 2,3-dioxygenase (IDO) activity. Such activity is induced by IFN-γ, in response to viral infections and plays an immunoregulatory role by limiting inflammation [18] (Additional file 1: Annex 3A.5).

#### Transcriptomics/CyTOF studies

##### Immune response in peripheral blood (BULK RNAseq)

Early SARS-CoV-2 infection triggers a powerful, IFN-driven transcriptional response in peripheral blood. Type I IFN response was impaired in severe and critical COVID-19 patients: striking downregulation of ISGs, IRF-1 and STAT3, absence of circulating IFN-β in patients with all disease-severity grades and low IFN-α production in severe COVID-19 patients were reported [TE90]. Moreover, elevated levels of chemokines and chemokine receptors were detected in severe patients, exhibiting an increase in neutrophils. Downregulation of negative regulators of innate immune system and TCR signaling kinases and adaptors was observed in severe patients (Additional file 1: Annex 3B).

##### Innate immune cell compartment (scRNAseq/CyTOF)

The initial local respiratory SARS-CoV-2 infection elicits dynamic changes of circulating blood cells with changes in innate immunity parameters. An elevated neutrophil/lymphocyte ratio has been identified as a sign of COVID-19 severity [TE89]. Investigation of the neutrophil transcriptomics signatures highlighted that excessive neutrophil activation is associated with severe COVID-19 more frequently than with mild disease. Moreover, Low Density Neutrophils with immature phenotype were up-regulated in severe disease [TE96, TE112]. An increase of classical CD14+ monocytes, especially in convalescence stages, non-classical CD16+ monocytes and natural killer (NK) cells also with exhaustion phenotype was observed [TE89, TE105, TE112, TE113]. Impaired IFN-α production by plasmacytoid dendritic cells was also observed in COVID-19 patients. However, some ISGs were up-regulated in monocytes and DC [TE89] (Additional file 1: Annex 3C).

##### Adaptive immune cell compartment (scRNAseq/CyTOF)

SARS-CoV-2 infection has a strong effect on the transcriptional profile of T and B cells. scRNAseq confirmed the drop in the percentage of circulating lymphocytes, including CD4+, and CD8+ T cells relative to increasing severity [TE89, TE112, TE113]. The increased expression of genes related to T cell apoptosis in COVID-19 patients may contribute to circulating lymphocyte depletion [TE83]. The upregulation of genes related to a strong T-cell response characterizes the immune response against SARS-CoV-2 in mild patients, reflecting T-cell signalling activation and T-cell differentiation, followed by rapid reduction thereafter. Differently, a negative T-cell signalling was persistently observed in severe patients along time. The transcriptional signatures of different T cells profiles types show that severe patients present an increase of naive T cells and a decrease in activated effector T cells as compared to mildly affected patients. Moderate COVID-19 patients present a proliferative exhausted CD8+ T cell subpopulation. This cell population has high cytotoxic signature, maintaining its naïve character. IL17-A and IL17-F are increased in COVID-19 patients [TE90], and genes related to IL17 signalling are significantly enriched in the severe-fatal group [TE96]. Finally, a significant activation of naïve B cells and expansion of antibody-secreting cells (ASCs) has been observed both in moderate and in severe patients, as compared to healthy and mild/asymptomatically infected subjects. (Annex 3D).

#### Immune response in lung and other tissues

Major deregulation of the innate immune response has been observed in lung samples. COVID-19 patient lungs showed a compartmentalization of innate immune cells (neutrophils and monocytes), in response to the chemokine secretion [TE106, TE108]. The transcriptional profiling of the lung tissue showed the over-expression of genes related to neutrophil activation and the generation of neutrophil extracellular traps (NETs), confirming the role of NETs formation in the immunopathology induced by SARS-CoV-2 infection.

Transcriptional profiling of nasopharyngeal swabs showed inflammatory response genes, IFN-α response, IL6/JAK/STAT3 signalling, and complement cascade activation in COVID-19 patients, while a down-modulation of anti-inflammatory pathways was observed by scRNAseq in CD14+ /CD16+ cells from severe patients [TE99].

The colon transcriptome of COVID-19 patients revealed an up-regulation of genes related to the response to TGF-β, whereas a down-modulation of genes involved in immune cell activation was found in fatal cases compared to healthy donors [TE110].

Proteomics analysis revealed the up-regulation of iNOS and IL1b and IL6 proteins in lung tissue of COVID-19 patients [TE113]. Moreover, proteomic analyses identified many key proteins, such as cathepsins B and L, and inflammatory response modulators, highly expressed and translated in fatal cases compared to healthy donors [TE110] (Additional file 1: Annex 3E).

### Phenotypes

We selected the studies with clear stratification of patients by disease severity; multi-omics data from these studies which highlighted significant differences between healthy controls and COVID-19 patients, and between mild and severe clinical presentation. We then classified the selected studies in four topics: (1) Key Genes and Proteins in SARS-CoV-2- host interactions and pathogenesis in the Lung; (2) DEG and DEP analysis in other organs and tissues; (3) Hub genes and pathways of innate immune response; (4) Comorbidities, further subclassified in comorbidities COVID-19-associated not sharing COVID-19 pathogenesis, and comorbidities associated and related to COVID-19 pathways, reported in Table 3, Additional file 1: Tables S5 and Annex 4.

**Table 3:** Pathogenic mechanisms in COVID-19 phenotype: SARS-CoV-2—host interactions in the lung. (A), DEG and DEP analysis in other organs and tissues (B) Hub genes and pathway of innate immune response (C), Comorbidities COVID19 associated not sharing COVID19 pathogenesis (D), Comorbidities associated and related to COVID-19 pathway (E)

A.SARS-CoV-2—host interactions in the lung		
Phenotypes	Pathways	Reactome code
Severe	Interferon type I signalling pathways ISGs and ACE2: gene highly expressed TMPRSS2 [TE107]	R-HSA-909733
	Metabolism of proteins Significant increase of antigens processing [TE108]	R-HSA-392499
	Cytokine Signalling in Immune system Upregulation of proinflammatory cytokine and chemokine genes [TE116]	R-HSA-128021
	Neutrophil degranulation Genes involved in neutrophil extracellular traps generation (NETs) [TE117]	R-HSA-6798695
	Disorders of transmembrane transporters Expression of the Lipopolysaccharide (LPS) sensors [TE114]	R-HSA-5619115
Mild/asymptomatic	Cytokine Signalling in Immune system Increasing of CCL2 chemokine [TE88]	R-HSA-1280215
Other infections	Interferon type I signalling pathways Increasing of five cytokines (IFNG, IL6, CXCL8, CXCL10 and CCL2) in in mild and severe COVID-19 patients than influenza [TE96]	R-HSA-909733

B. DEG and DEP analysis in other organs and tissues		
Phenotypes	Pathways	Reactome code
Severe	TMPRSS2 Mediated SARS-CoV-1 Spike Protein Cleavage and Endocytosis; ACE2 overexpression [TE121]	R-HSA-2022344
	Attachment and Entry Altered expression of ACE2 in intestinal tissue, as effect of SARS-CoV-2 ACE2 exhibits the highest co-expression correlation with TMPRSS2, SLC6A20C [TE121, TE123]	R-HSA-9678110
	Transport of small molecules Decrease of brain-enhanced proteins that regulate neurotransmitter synthesis (GLS, OGDH, DLD, etc.), neurotransmitter transport (GLUL, GLUD2, GLUD1), neurotransmitter receptors (HTRA3) [TE96]	R-HSA-382551
Mild/asymptomatic	Diseases of haemostasis Upregulated serum proteins, such as S100A8, S100A9, serum amyloid A1 (SAA1) [TE94]	R-HSA-9671793

C. Hub genes and pathways of innate immune response		
Phenotypes	Pathways	Reactome code
Severe	ISG15 antiviral mechanism IFN deficiency in the blood [TE130, TE89]	R-HSA-1169408
	Cytokine Signalling in Immune system Robust levels of chemokines, including CCL2, CCL8, and CCL11. Significant increase in circulating IL6, IL1RA levels along with CXCL9 and CXCL16, CCL8 and CCL2 [TE87]	R-HSA-1280215
	Clathrin-mediated endocytosis Clathrin-mediated endocytosis signalling, actin cytoskeleton signalling, mechanisms of viral exit from host cells [TE136]	R-HSA-8856828
	Toll-like Receptor Cascades Significantly upregulated NETs [TE137]	R-HSA-168898
Mild/asymptomatic	Interferon-α/β signaling Early, transient type I IFN production in the lung, that induces ISGs in the peripheral blood [TE89]	R-HSA-909733
	Programmed Cell Death T cell apoptosis [TE111]	R-HSA-5357801

D. Comorbidities COVID19 associated not sharing COVID19 pathogenesis		
Phenotypes	Pathways	Reactome code
Severe	Histone Modifications (Post-translational protein modification) Several genes related to histone modifications (HAT1, HDAC2, KDM5B) were identified in severe COVID19 patients with comorbidities. [TE119]	R-HSA-597592
	TMPRSS2 Mediated SARS-CoV-1 Spike Protein Cleavage and Endocytosis Several genes positively associated with ACE2 are regulated by KDM5B, and by specific histone acetylation. [TE119]	R-HSA-2022344

E. Comorbidities associated and related to COVID-19 pathway		
Phenotype	Pathways	Reactome code
Severe	Attachment and Entry Increased circulating furin levels that could cleave the spike protein and increase SARS-CoV-2 infectivity [TE121]	R-HSA-9678110
	Glycerophospholipid biosynthesis Lipids quantified in the negative mode (arachidonic acid, oleic acid, glycerophosphoethanolamines, and glycerophosphoethanolamines); Two significant pathways: fat digestion and adsorption and glycerophospholipid metabolism. [TE145]	R-HSA-1483206
Mild/asymptomatic	Glycerophospholipid biosynthesis Non-critical patients are characterized by strong alteration of lipids, including acylcarnitines [TE90]	R-HSA-1483206
	Tryptophan catabolism Metabolic alteration in mild/asymptomatic COVID19 patients include: altered tryptophan metabolism into the kynurenine pathway, regulating inflammation and immunity [TE146]	R-HSA-71240
	Bacterial infection Lipid rafts are associated with SARS-CoV-2 virulence [TE143]	R-HSA-9664407

Omics and pathway involved were split by different phenotypes: severe, mild/asymptomatic and other infections. Differentially Expressed Proteins and Differentially Expressed Genes (DEP and DEG, respectively) are detailed in Additional file 1: Table S5

As shown in Table 3A, our review confirms that SARS-CoV-2 RNA is highly localized in cells that express TMPRSS2, especially ciliated and secretory cells in the airway epithelium, and Alveolar Type 1 (AT1) cells in the lung [TE115]. As reported before, transcriptomics and proteomics analysis show the pathways more involved in patients with severe disease [TE107, E108, TE110, TE116, TE117]. Both mild and severe COVID-19 patients present elevation of chemokines associated with lung inflammatory disorders, such as acute respiratory distress syndrome, asthma, and pulmonary fibrosis [TE88].

Genomics highlights that chromosome 3 is significantly associated with respiratory failure, since in its loci genes functionally interacting with *ACE2* are located [TE130].

In Table 3B, pathways most involved in severe COVID- 19, highighted by transcriptomics and proteomics data on gastrointestinal, genital and neurologic departments, are reported. The potential susceptibility of these tissues to SARS-CoV-2 entry is due to the high co-expression of ACE2 and TMPRSS2 [TE122- TE127]. However, in these organs SARS-CoV-2 does not determine the damage observed in the respiratory tract, suggesting that ACE2/TMPRSS2 expression alone is not sufficient to mediate the tissue injury.

As shown in Table 3C, omics data highlight a dysregulation of innate immune response-related pathways in severe patients [TE131, TE132]. Both mild and severe patients present a significant downregulation of TCA and of glycolytic pathways, and upregulation of HIF1A signalling and host defense pathways. Proteome host signatures indicate high specificity of several inflammatory modulators, particularly IL6, IL1B, and TNF [TE138], as confirmed by the significant increase of these modulators observed in severe patients in clinical studies [TE96]. Immunosuppression and tight junction impairment occurs in the early phase of COVID-19, while the immune response is activated later [TE135]. The defective monocyte activation, combined with the dysregulated myelopoiesis, observed in patients with severe disease, may cause a continuous state of inflammation and ineffective host immune response [TE112].

The inflammation described in severe COVID-19 is also reflected by metabolomics and lipidomics data, which show imbalanced homeostasis of glycolysis, lipogenesis, heme and ketone biosynthesis, gluconeogenesis, fatty acids oxidation, and cholesterol biosynthesis, through the activation of β-oxidation pathways [TE109]. Metabolomics and lipidomics characterize the difference between mild and severe forms in quantitative terms, and are mostly found in COVID-19 phenotypes associated with comorbidities. The strong association between inflammation and metabolic alterations allows to identify two groups of comorbidities: (1) COVID-19-associated diseases that increase patient frailty by COVID-19-independent pathogenic mechanisms (e.g., chronic heart disease); (2) COVID-19-associated diseases that increase patient frailty by COVID-19-dependent pathogenic mechanisms (e.g., diabetes) (Table 3D, E).

Genomics studies suggest that ACE2 polymorphisms might be associated with cardiovascular and pulmonary conditions by altering the AGT-ACE2 interactions, and transcriptomics data confirm the upregulation of the gene encoding ACE2 receptor in lung tissue in several comorbidities associated with severe COVID-19, such as COPD or PAH, and even in people who smoke.

Diabetes is the best described co-morbidity related to COVID-19 pathways. This complex metabolic disease is able to complicate COVID-19 by several mechanisms: (1) presence of bone marrow changes, predisposing to excessive proinflammatory response and contributing to insulin resistance, reducing vascular repair and worsening function of heart, kidney, and systemic vasculature; (2) increased circulating FURIN levels, that cleaves the S glycoprotein; (3) dysregulated autophagy, that may promote replication and/or reduce viral clearance; (4) gut dysbiosis, leading to widespread systemic inflammation, increased glucose and sodium absorption, and reduced absorption of tryptophan needed for glucose homeostasis [TE142].

COVID-19 patients showed relevant changes in serum levels of lipoprotein subclasses and their components [TE94, TE100, TE102], mainly reflecting the metabolic pathways of lysine degradation, metabolism of taurine, hypotaurine, alphalinolenic acid, glycerophospholipid, arginine, proline, and arginine biosynthesis [TE145]. Severe patients are characterized by pathways listed in Table 3E, possibly linked to a reduced hepatic capacity to oxidize acetyl-CoA in the mitochondria, consistent with serum glucose elevation [TE104].

The technique of transcriptomics per single cell (scRNAseq) contributed also to better understand the etiology of COVID-19 neurological sequelae, although further analyses are needed [TE124].

## Discussion

This review highlights that omics technologies could significantly contribute to increase knowledge of COVID-19 pathophysiology. However, it also highlights that disease mechanisms are too complex to be explained by a single molecular signature, even if identified by a multi-omics approach, and an integrated approach is necessary to identify hallmarks of severity. In fact, specific omics techniques are useful to study specific organs and/or tissues and diseases phases, but a single omics cannot explain the complexity of pathways, as shown in Fig. 3, where the same pathway is reported to be eventually up- or down-regulated in SARS-CoV-2 infection and in the same severity profile.

The first step in COVID-19 understanding was the genomics and proteomics characterization of the new infectious agent in relation to other coronaviruses. Omics data helped to decipher the perturbation induced by SARS-CoV-2 in miRNA, highlighting possible interactions between viral genomes and host miRNAs. Moreover, they allowed to identify small non-coding viral RNA participating in the pathogenesis of lung disease and to identify regions of SARS-CoV-2 genome as potential sites for RNA silencing.

To reconstruct pathological interactions between virus and host, close to what happens in vivo, it is necessary to combine in silico and in vitro data, integrating as much as possible different kinds of omics data. The integrated multi-omics approach categorizes the most important molecular interactions, allowing the creation of a disease map, able to elucidate pathogenetic interactions between pathogen and host. In this view, results deriving from ex vivo studies should be preferred as compared to those coming from in vitro studies, since the latter may have several limitations, and are usually performed using immortalized cell line, derived in some cases from non-human hosts.

Besides virus structure and entry mechanisms, the omics approach allows to identify systemic effectors, and to identify pathways and host signatures correlated with both stage and severity of the disease, mirroring new aspects of COVID-19.

Particularly interesting were the findings regarding changes in oxidative pathways and in lipid metabolism. Alterations of the levels of tricarboxylic acid cycle (TCA) metabolites indicate a metabolic response to declining lung functions and to decreasing blood oxygen, but also reflect a response to nutrition changes, especially in severe patients, leading to glucose elevation as compensatory increase of gluconeogenesis [TE100]. The dysregulated metabolism of glyoxylate and dicarboxylate is also indicator of energy metabolic dysfunction. In moderate and severe patients, most of the lipidomics studies show alterations suggesting an increase in adipose tissue lipolysis [TE101, TE146].

The production of bioactive lipid mediators, such as prostaglandins, leukotrienes, and lysophospholipids, important for viral replication, contribute to form specialized membrane compartments for viral replication and to immune cell infiltration during vascular inflammation [19–21]. Moreover, LPCs potentiate the activation of T lymphocytes, macrophages, and neutrophils [22]. The significant decrease of amino acids and their derivatives [TE100, TE101, TE102, TE103, TE104] could indicate liver dysfunction [23].

Omics approach highlights that the early phase of the infection elicits a strong activation of innate immunity and an increase of APPs, followed by the “cytokine storm”. The upregulation of APPs induces inflammatory cytokines and neutrophil activation in the early phase, but also contributes to activate pathways involved in the subsequent stages of the disease, such as amplification of the cytokine storm, platelet aggregation, activation of coagulation cascades and complement system, and alteration of lipid metabolism.

However, the entity of these responses is correlated to disease severity: mild disease is characterized by increase of IFN-α, IFN-γ, IL6, IL10, IL1RA [TE80, TE91, TE92]; severe disease is characterized by a downregulation of IFN-α and up-regulation of IFN-β, IFN-γ, IL6, TNF, IL10, CCL7, IL8, and IL12 [TE83, TE90, TE91, TE96,TE97], expression of exhaustion-related genes, such as BATF, IRF4, and CD274 (PD-L1) [TE92]; critical disease is characterized by the upregulation of IL6, TNFs, IL10, RIPK3. The low IFN-α levels correlate with the reduction and functional impairment of plasmacytoid dendritic cells (pDCs), the main cell source of IFN-α. Thus, type I IFN deficiency could be a severity indicator in COVID-19 [TE89]. Severity-dependent connections between specific upregulated cytokines and metabolic processes downregulation suggest a down-up regulation between increasing disease severity, elevated inflammation and loss of key circulating nutrients. Particularly, omics approach evidences a significant similarity between moderate and severe COVID-19 and large difference between mild and moderate diseases [TE90].

Most of the studies analyzed in this review reveal that severity appears closely related to dysfunction of platelet degranulation and of coagulation cascades [TE92, TE95, TE96, TE97]. This was demonstrated by: elevation of components and regulators of the complement system; increased expression of thrombotic pathway genes and SERPINE1; decreased expression of PROS; increased levels of D-dimer and fibrinogen degradation products; decreased expression of F13A1 [TE96, TE97]. These evidences are consistent with several non-omics-based clinical studies, reporting a correlation between increased D-dimer concentration and poor prognosis. Other markers of coagulopathy, such as platelet counts and prothrombin time, were viewed as progressively dysregulated with worsening patient conditions [TE147, TE148].

In accordance with those findings, autoptic analysis on fatal COVID-19 cases highlighted high incidence of pulmonary macroemboli and occlusion of alveolar capillaries, revealing severe endothelial injury, increased angiogenesis and microemboli [TE149, TE150]. Only few evidences reported that early stages of COVID-19 are featured by downregulation of complement and coagulation cascades, compared to other viral infections [TE89].

Severity seems also related to changes in PBMCs: expression of neutrophil surface receptors (CXCR1, SINGLEC5, and CD177), neutrophil granule contents (DEFA1) and transcriptomics signatures related to neutrophil activation, chemotaxis, degranulation and migration, are dramatically upregulated in severe compared to mild patients. Other hallmarks of COVID-19 severity are elevated neutrophil/lymphocyte ratio [TE89] and HLA-DR down-modulation in monocytes [TE90].

Transcriptomics studies on local mucosal immune response at upper and lower respiratory tract demonstrate the induction of genes related to immune modulatory functions, including inflammatory response, IFN-α response, IL6/JAK/STAT3 signalling, neutrophil activation, and generation of NETs [TE99, TE110, TE111].

Transcriptomics studies on other districts (e.g., colon) suggest that SARS-CoV-2 infection might influence host responses in body districts not directly hosting the COVID-19 infection, sustaining the inflammatory processes even in sites without the direct viral injury [TE110].

SARS-CoV-2 infection has a strong effect on adaptive immune response, as shown by the transcriptional profile of T and B cells during the infection: the drop of circulating lymphocytes (including CD4+ and CD8+) is related to increased severity and might be due to the over-expression of genes inducing T cell apoptosis [TE83, TE89, TE110, TE111, TE112].

Proteomics and transcriptomics analyses showed that patients with mild COVID-19 present a robust T-cell response while severe patients show a negative T-cell signalling [TE83, TE89, TE96].

Several studies comparing the expression of cytokines and their receptors, and transcription factors in T cells subset, showed that upregulated genes mostly encode proinflammatory cytokines, as well as their respective receptors, and IFN-stimulated transcription factors, indicating a possible involvement of CD4+ T cells in the major inflammatory responses to cytokines. Plasma cells are also significantly increased [TE105, TE111]. Particularly, severe COVID-19 is characterized by elevation in plasmablasts/plasma cells [TE98] and by a significant activation of naive B cells [TE111].

The heterogeneity of COVID-19 phenotypes remains one of the key questions, just partially explained, and the omics approach might contribute to a better defining the specific mechanisms, characterizing and determining clinical phenotypes.

Like other respiratory infections, SARS-CoV-2 initially activates inflammatory mechanisms in the respiratory tract. Subsequently, the inflammation spreads to other organs and tissues. ACE2 expression in lung cells surely represents an entry factor for SARS-CoV-2, but it is not enough to explain why the damage of SARS-CoV-2 mainly occurs at lung level. Transcriptomics at the lung level optimally characterizes severe forms, showing the activation of several pathways such as Type I INF signalling, histone modifications, neutrophil degranulation, and disorders of transmembrane transporters, and particularly a global dysregulation of immune-related pathways in severe patients.

Metabolomics and lipidomics well characterized in quantitative terms the difference between mild and severe forms, and are particularly useful to study the role of comorbidities. Several amino acid profiles observed in COVID-19 are also observed during acute hepatic failure [24], insulin resistance and increased risk of Type-2 diabetes [25]. In addition, the increase of creatine, creatinine, polyamines spermidine and acetyl-spermidine could suggest renal dysfunction [26] [TE94, TE103, TE104]. These characteristics are well known features of increased risk of cardiovascular disease, diabetes, liver and renal diseases, and they could therefore explain the increased severity of COVID-19 in patients with these co-morbidities.

As discussed above, COVID-19 severity is also linked to dysfunction of coagulation cascades. Based on clinical courses and coagulation parameters, three stages of clinical COVID-19 coagulopathy can be defined [TE151]:

- Stage 1: mild systemic inflammation and coagulopathy, with patients having mild symptoms and no need for respiratory support;
- Stage 2: progressive pulmonary inflammation, coagulopathy in pulmonary alveoli and microthrombosis, with patients developing more severe symptoms and often requiring additional oxygen supply;
- Stage 3: strong pro-inflammatory reaction, development of local and systemic coagulopathy, characterized by high D-dimer and fibrinogen concentration, prolonged prothrombin time, reduced platelet counts, and high incidence of deep vein thrombosis (DVT) or pulmonary embolism (PE); patients’ conditions deteriorate, requiring organ support, in particular mechanical ventilation, including extracorporeal membrane oxygenation (ECMO) [TE108].

This sequence of events brings to the death of pneumocytes, along with pulmonary thrombotic microangiopathy (TMA), causing severe clinical conditions with high need for oxygen supply. In this context, the increased D-dimer and enhanced platelet activation levels are strongly linked with pathogenic pro-coagulant phenotype. In turn, the cytokine storm triggers inflammatory processes, bring to systemic endothelitis, cellular and organ dysfunction (i.e. acute respiratory distress syndrome, ARD), and activation of coagulation cascade [27–30].

## Limitations

Limits of this review are intrinsically related to the limits of the omics techniques. In fact, the current omics platforms have several technical limits and are not standardized for the translational use in a clinical context. Many aspects of pathogenetic mechanisms can be investigated by multi-omics approach, but biological mechanisms not yet identified by omics analysis may exist.

Moreover, we have considered the integration between different omics layers as a whole, without carrying out more in-depth analyses, which would have allowed the identification of further suitable markers of disease severity.

Even if clinical studies eventually show good correspondence with the observed omics data, their number is still quite small and some of them are editorials or observational studies. In addition, there are very few omics data regarding patients after hospital discharge. Clinical studies describe the persistence of functional respiratory limitations few months after discharge [31]; an omics approach to these “Long COVID” clinical observations could contribute elucidating pathogenic mechanisms involved in the medium- and long-term complications.

This review considered evidences up to the end of February 2021. It could be useful to periodically update it, considering the fast-growing bulk of evidences.

## Conclusions

In conclusion, multi-omics approach appeared to be useful in evaluating the host response and in identifying markers of clinical severity.

In particular, it revealed that: (i) the interactome can be considered as an in silico helpful model to investigate the viral-host interactions and to identify involved pathways; (ii) commonly dysregulated pathways of innate immune responses (e.g., complement activation, inflammatory responses, IFN system activation, neutrophil activation and degranulation, platelet degranulation and dysregulation of blood coagulation) can affect the clinical progression of COVID-19.

Multi-omics approach can therefore contribute to elaborate disease maps, identifying pathogenic mechanisms involved in the development of disease phenotypes and outcomes. Our work considered only the most common omics techniques, but it could be extended to other omics types, such as epigenomics (DNA methylation, histone modifications), glycomics, and long-noncoding RNA data. Several clinical observations are consistent with evidences highlighted by the omics studies analyzed in this review. Finally, the results suggest that multi-omics approach may be useful to further investigate unknown aspects of the disease, such as, for instance, the mechanisms involved in the long-term effects of COVID-19, the COVID-19 neurological involvement, and the Kawasaki-like COVID-19 multi-system inflammatory syndrome in children (MIS-C).

## Supplementary Information

**Additional file 1.** Scoping review protocol. Working method and tools used by the domain groups. Table S1. Selected articles catalogued by domain. Table S2. Tables of evidence. A) Virus characterization and viral entry: B) Host signature: C) Pathways: D. Phenotypes. Table S3. B) Conceptual table: SARS-CoV-2 entry. Tabla S4. Immune response to SARS-CoV-2 infection in lung and other tissues (A), peripheral blood (B) and specific cell types in blood immune cells (C). Pathways, host signature (differentially expressed genes and proteins) and body districts, subset per specific omics data: host proteomics, bulk RNAseq and scRNAseq. Table S5. Pathogenic mechanisms in COVID-19 phenotype: SARS-CoV-2—host interactions in the lung. (A), DEG and DEP analysis in other organs and tissues (B) Hub genes and pathway of innate immune response (C), Comorbidities COVID-19 associated not sharing COVID-19 pathogenesis (D), Comorbidities associated and related to COVID-19 pathway (E). Omics and pathway involved were split by different phenotypes: severe, mild/asymptomatic and other Infections. Annexes: Annex 1—Report from Working Group 1: Molecular characterisation of the virus; Annex 2—Report from Working Group 2—Pathways; Annex 3—Report from Working Group 3- Host signatures; Annex 4—Report from Working Group 4—Phenotype. Glossary.

**Additional file 2: Table S3.** A) Viral characterization—Conceptual table.

**Additional file 3: Table S6.** Pathways analysis based on Reactome Pathways Relation.

**Additional file 4: Table S7.** Detailed table of evidences.

## Data Availability

Data were taken from PubMed repository (https://pubmed.ncbi.nlm.nih.gov/). The results of scoping review research were reported in Additional file 4: Table S7.
