# Supplementary material for: Multi-omics approach to COVID-19: a domain-based literature review
Source: J Transl Med. 2021 Dec 7;19:501. doi: 10.1186/s12967-021-03168-8 (PMC8649311; doi:10.1186/s12967-021-03168-8)
Supplement: Supplementary file 1 — Additional file 1. Scoping review protocol. Working method and tools used by the domain groups. Table S1. Selected articles catalogued by domain. Table S2. Tables of evidence. A) Virus characterization and viral entry: B) Host signature: C) Pathways: D. Phenotypes. Table S3. B) Conceptual table: SARS-CoV-2 entry. Tabla S4. Immune response to SARS-CoV-2 infection in lung and other tissues (A), peripheral blood (B) and specific cell types in blood immune cells (C). Pathways, host signature (differentially expressed genes and proteins) and body districts, subset per specific omics data: host proteomics, bulk RNAseq and scRNAseq. Table S5. Pathogenic mechanisms in COVID-19 phenotype: SARS-CoV-2—host interactions in the lung. (A), DEG and DEP analysis in other organs and tissues (B) Hub genes and pathway of innate immune response (C), Comorbidities COVID-19 associated not sharing COVID-19 pathogenesis (D), Comorbidities associated and related to COVID-19 pathway (E). Omics and pathway involved were split by different phenotypes: severe, mild/asymptomatic and other Infections. Annexes: Annex 1—Report from Working Group 1: Molecular characterisation of the virus; Annex 2—Report from Working Group 2—Pathways; Annex 3—Report from Working Group 3- Host signatures; Annex 4—Report from Working Group 4—Phenotype. Glossary. [file 12967_2021_3168_MOESM1_ESM.docx]

**Supplementary materials**

[1. Scoping review protocol 2](#_Toc72411813)

[2. Working method and tools used by the domain groups 2](#_Toc87967410)

[3. Table S1. Selected articles catalogued by domain. 3](#_Toc87967411)

[4. Table S2. Tables of evidence. 4](#_Toc87967412)

[Table S2 A. Virus characterization and viral entry. 4](#_Toc87967413)

[Table S2 B. Host signature. 13](#_Toc87967414)

[Table S2 C. Pathways. 15](#_Toc87967415)

[Table S2 D. Phenotypes. 20](#_Toc87967416)

[5. Table S3 A. Viral characterization - Conceptual table (Additional file 2). 27](#_Toc87967417)

[6. Table S3 B. SARS-CoV-2 entry – Conceptual table. 28](#_Toc87967418)

[8. Table S4. Immune response to SARS-CoV-2 infection in lung and other tissues (A), peripheral blood (B) and specific cell types in blood immune cells (C). Pathways, host signature (differentially expressed genes and proteins) and body districts, subset per specific omics data: host proteomics, bulk RNAseq and scRNAseq. 30](#_Toc87967419)

[9. Table S5. Pathogenic mechanisms in COVID-19 phenotype: SARS-CoV-2 - host interactions in the lung. (A), DEG and DEP analysis in other organs and tissues (B) Hub genes and pathway of innate immune response (C), Comorbidities COVID-19 associated not sharing COVID-19 pathogenesis (D), Comorbidities associated and related to COVID-19 pathway (E). Omics and pathway involved were split by different phenotypes: severe, mild/asymptomatic and other Infections. 35](#_Toc87967420)

[10. Table S6. Pathways analysis based on Reactome Pathways Relation (Additional file 3). 41](#_Toc87967421)

[11. Table S7. Detailed table of evidences (Additional file 4). 41](#_Toc87967422)

[12. Annexes 41](#_Toc87967423)

[**Annex 1 – Report from Working Group 1 - Molecular characterisation of the virus** 41](#_Toc87967424)

[**Annex 2 – Report from Working Group 2 – Pathways** 46](#_Toc87967425)

[**Annex 3 – Report from Working Group 3 - Host signatures** 48](#_Toc87967426)

[**Annex 4 – Report from Working Group 4 – Phenotype** 54](#_Toc87967427)

[11. Glossary 60](#_Toc87967428)

1. **Scoping review protocol**
   Focusing on omics data, virus molecular characterization, and clinical and immunological findings to identify pathways and phenotypes of COVID-19, useful to build a disease model.

**Population (Who):** Human coronavirus (HCoV) diseases with mild, moderate, and severe outcomes. Replication cycle of HCoV, and host responses occurring in the different stages of HCoV replication. SARS CoV-2 characteristics and COVID-19 specific features.

**Concept (What):** summarize the current knowledge about virus characteristics, pathways involved during HCoV infection and disease phenotypes. In the light of the pathogenesis of the previously known coronavirus, a literature analysis was carried out about SARS-CoV-2 pathophysiology, its molecular interactions with specific gene/protein targets and the immune response to the virus, including dysfunctional immune responses to disease progression. Specifically, highlight the implications of specific features of the infection, omics data (signatures) found in populations of subjects with COVID-19 in order to identify possible phenotypes correlated with immune response patterns, radiological patterns, and clinical outcomes.

**Context (with what qualifiers):** We plan to evaluate the relevant scientific studies of the last 18 years, published in English, conducted on humans and non-human primates, as well as published databases covering clinical, radiological, immunological, epidemiological and treatment information, based on an integrated approach (including omics data, epidemiology, molecular diagnostics, immunology).

 RESEARCH METHODOLOGY:

**Inclusion criteria**

Will be included in the review papers written in English that report on studies conducted on human patients or non-human primates in different fields of the HCoV disease and contain both omics data and immunological/clinical / epidemiological information. Will be included papers published since January 1 2002 for which omics data are available and may be downloaded from open access databases.

**Information sources**

Articles in English of the last 18 years will be identified by searching the electronic databases MEDLINE via PubMed, Web of Science, Scopus and Google Scholar, Google Scholar, was included to enable the search for the key terms also in the full text of the manuscript.

Moreover, relevant studies will be also identified by searching on the following platforms: bioRxiv, medRxiv.

**Search string**

The literature was made in PubMed using the keywords: “HCoV disease” and/or “SARS-CoV” and or “MERS” and/or “SARS-CoV-2” and /or “COVID-19” in combination with other search terms that should be included are: “omics”, “genomics”, “epigenomics”, “proteomics”, “transcriptomics”, “metabolomics”, “modelling”, “network”, “interactome”, “disease model”, “disease map”, .The literature search made also in others platforms and in omics repository data base using the keywords: HCoV disease” and/or “SARS-CoV” and/or “MERS” and/or “SARS-CoV-2” and /or “COVID-19”.

## **Working method and tools used by the domain groups**

The evidences catalogued by domain and select for review are reported on Table S1. Each article selected for review was independently read and evaluated by two reviewers.

A checklist opportunely adapted from the reference methodology (PRISMA-ScR) was used for the assessment. Data extraction was done using a predetermined card format (identifier, population, concept, contest, main results). At the end of the evaluation, the data sheets were discussed and evaluated in a specific meeting of the group of domain experts. Each interdisciplinary group of domain experts discussed and approved a report regarding the major results of the domain. Each domain group have drawn up an evidence table and one or more conceptual tables, by subdomain separated and crossing pathways and kinds of omics.

## **Table S1. Selected articles catalogued by domain.**

|  | **VIRUS** | **PHENOTYPES** | **PATHWAYS** | **TOT** |
| --- | --- | --- | --- | --- |
| Articles selected by the string | 299 | 402 | 513 | 1214 |
| Selected by title | 127 | 181 | 383 | 691 |
| Selected by abstract review | 74 | 119 | 177 | 370 |
| Further selected by full text review | 73 | 48 | 56 | 177 |

## **Table S2. Tables of evidence.**

### Table S2 A. Virus characterization and viral entry.

| **ID TE** | **URL** | **Title** | **Domain/sub-domains** | **Investigation field** | **First Author (et al.)** | **Journal** | **Year** |
| --- | --- | --- | --- | --- | --- | --- | --- |
| **1** | PMID: 32007145 | Genomic characterisation and epidemiology of 2019 novel coronavirus: implications for virus origins and receptor binding | VIRUS CHARACTERIZATION | Genome evolution and geographical distribution | Lu R | Lancet | 2020 |
| **2** | PMID: 32742815 | Understanding genomic diversity, pan-genome, and evolution of SARS-CoV-2 | VIRUS CHARACTERIZATION | Genome evolution and geographical distribution | Parlikar A | PeerJ | 2020 |
| **3** | PMID: 32793182 | Geographic and Genomic Distribution of SARS-CoV-2 Mutations | VIRUS CHARACTERIZATION | Genome evolution and geographical distribution | Mercatelli D | Front Microbiol | 2020 |
| **4** | PMID: 32474553 | Analysis of RNA sequences of 3636 SARS-CoV-2 collected from 55 countries reveals selective sweep of one virus type | VIRUS CHARACTERIZATION | Genome evolution and geographical distribution | Biswas NK | Indian J Med Res | 2020 |
| **5** | PMID: 32841689 | A genetic barcode of SARS-CoV-2 for monitoring global distribution of different clades during the COVID-19 pandemic | VIRUS CHARACTERIZATION | Genome evolution and geographical distribution | Guan Q | Int J Infect Dis | 2020 |
| **6** | PMID:  33605421 | Comparative Genomics reveals early emergence and Biased Spatiotemporal Distribution of SARS-CoV-2 | VIRUS CHARACTERIZATION | Genome evolution and geographical distribution | Chiara M | Mol Bio Evol | 2021 |
| **7** | PMID:  33184173 | Analysis of genomic distributions of SARS-CoV-2 reveals a dominant strain type with strong allelic associations | VIRUS CHARACTERIZATION | Genome evolution and geographical distribution | Yang HC | PNAS | 2021 |
| **8** | PMID:  33581339 | Compositional Variability and Mutation Spectra of Monophyletic SARS-CoV-2 Clades | VIRUS CHARACTERIZATION | Genome evolution and geographical distribution | Teng X | Genom Proteom Bioinf | 2021 |
| **9** | PMID: 33170902 | SARS-CoV-2 genomes reveals a clonal geo-distribution and a rich genetic variations of hotspots mutations | VIRUS CHARACTERIZATION | Genomic hotspots for mutation, drivers of evolution and correlation with pathogenesis | Laamarti M | PLoS One | 2020 |
| **10** | PMID: 32522874 | Genomic determinants of pathogenicity in SARS-CoV-2 and other human coronaviruses | VIRUS CHARACTERIZATION | Genomic hotspots for mutation, drivers of evolution and correlation with pathogenesis | Gussow AB | Proc Natl Acad Sci U S A | 2020 |
| **11** | PMID: 33161087 | Trends of mutation accumulation across global SARS-CoV-2 genomes: Implications for the evolution of the novel coronavirus | VIRUS CHARACTERIZATION | Genomic hotspots for mutation, drivers of evolution and correlation with pathogenesis | Roy C | Genomics | 2020 |
| **12** | PMID:  33088633 | Positive selection within the genomes of SARS-CoV-2 and other Coronaviruses independent of impact on protein function | VIRUS CHARACTERIZATION | Genomic hotspots for mutation, drivers of evolution and correlation with pathogenesis | Berrio A | Peer J. | 2020 |
| **13** | PMID: 32723797 | Comparative Genomic Analysis of Rapidly Evolving SARS-CoV-2 Reveals Mosaic Pattern of Phylogeographical Distribution | VIRUS CHARACTERIZATION | Genomic hotspots for mutation, drivers of evolution and correlation with pathogenesis | Kumar R | mSystems | 2020 |
| **14** | PMID: 32470119 | Coronavirus3D: 3D structural visualization of COVID-19 genomic divergence | VIRUS CHARACTERIZATION | Genomic hotspots for mutation, drivers of evolution and correlation with pathogenesis | Sedova M | Bioinformatics | 2020 |
| **15** | PMID: 33024961 | SARS-CoV-2 gene content and COVID-19 mutation impact by comparing 44 Sarbecovirus genomes | VIRUS CHARACTERIZATION | Genomic hotspots for mutation, drivers of evolution and correlation with pathogenesis | Jungreis I | bioRxiv [Pre-print]. | 2020 |
| **16** | PMID:  30310104 | Attenuation of replication by a 29 nucleotide deletion in SARS-coronavirus acquired during the early stages of human-to-human transmission | VIRUS CHARACTERIZATION | Genomic hotspots for mutation, drivers of evolution and correlation with pathogenesis | Muth D | Sci Rep | 2018 |
| **17** | PMID: 32694143 | Discovery and genomic characterization of a 382-nucleotide deletions in ORF7b and ORF8 during the early evolution of SARS-CoV-2 | VIRUS CHARACTERIZATION | Genomic hotspots for mutation, drivers of evolution and correlation with pathogenesis | Su YCF | m Bio | 2020 |
| **18** | PMID: 32822564 | Effects of a major deletion in the SARS-CoV-2 genome on the severity of infection and the inflammatory response: an observational cohort study | VIRUS CHARACTERIZATION | Genomic hotspots for mutation, drivers of evolution and correlation with pathogenesis | Young BE | Lancet | 2020 |
| **19** | PMID: 33086379 | Specific re- distribution of SARS-CoV-2 variants in the respiratory system and intestinal tract | VIRUS CHARACTERIZATION | Intrahost genomic variablity | Du P | CID | 2020 |
| **20** | PMID: 32818852 | SARS-CoV-2 exhibits intra-host genomic plasticity and low-frequency polymorphic quasispecies | VIRUS CHARACTERIZATION | Intrahost genomic variablity | Karamitros T | J Clin Virol | 2020 |
| **21** | PMID: 32129843 | Genomic Diversity of Severe Acute Respiratory Syndrome-Coronavirus 2 in Patients with Coronavirus Disease | VIRUS CHARACTERIZATION | Intrahost genomic variablity | Shen Z | Clin Infect Dis | 2019 |
| **22** | PMID: 32858978 | Compartmentalized Replication of SARS-CoV-2 in Upper vs. Lower Respiratory Tract Assessed by Whole Genome Quasispecies Analysis | VIRUS CHARACTERIZATION | Intrahost genomic variablity | Rueca M | Microorganisms | 2020 |
| **23** | PMID: 33618765 | Intra-host variation and evolutionary dynamics of SARS-CoV-2 populations in COVID-19 patients | VIRUS CHARACTERIZATION | Intrahost genomic variablity | Wang Y | Genome Med | 2021 |
| **24** | PMID:  26304538 | Discovery of an essential nucleotidylating activity associated with a newly delineated conserved domain in the RNA polymerase-containing protein of all nidoviruses | VIRUS CHARACTERIZATION | Single viral proteins | Lehmann KC | Nucleic Acids Res | 2015 |
| **25** | PMID:  32283146 | Evolutionary analysis of SARS-CoV-2: how mutation of Non-Structural Protein 6 (NSP6) could affect viral autophagy | VIRUS CHARACTERIZATION | Single viral proteins | Benvenuto D | J Infect | 2020 |
| **26** | PMID: 16212442 | Prediction of Functional Class of the SARS Coronavirus Proteins by a Statistical Learing Method | VIRUS CHARACTERIZATION | Single viral proteins | Cai CZ | J Proteome Res | 2005 |
| **27** | PMID: 32941612 | Potential pathogenicity determinants identified from structural proteomics of SARS-CoV and SARS-CoV-2 | VIRUS CHARACTERIZATION | Single viral proteins | Prates ET | Mol Biol Evol | 2020 |
| **28** | PMID: 32754056 | SARS-CoV-2 Molecular Network Structure | VIRUS CHARACTERIZATION | Whole viral proteome | Diaz J | Front Physiol | 2020 |
| **29** | PMID: 32587094 | SARS-CoV-2 (COVID-19) structural and evolutionary dynamicome: insights into functional evolution and human genomics | VIRUS CHARACTERIZATION | Whole viral proteome | Gupta R | J Biol Chem | 2020 |
| **30** | PMID: 33024578 | Benchmarking evolutionary tinkering underlying human-viral molecular mimicry shows multiple host pulmonary-arterial peptides mimicked by SARS-CoV-2 | VIRUS CHARACTERIZATION | Immune proteomics | Venkatakrishnan AJ, | Cell Death Discov | 2020 |
| **31** | PMID:  32832263 | Anti-COVID-19 multi-epitope vaccine designs employing viral genome sequences | VIRUS CHARACTERIZATION | Immune proteomics | Zaheer T | Peer J | 2020 |
| **32** | PMID: 32577654 | Landscape and Selection of Vaccine Epitopes in SARS-CoV-2 | VIRUS CHARACTERIZATION | Immune proteomics | Smith CC | bioRxiv [Pre-print]. | 2020 |
| **33** | PMID: 33068416 | Structural analysis of SARS-CoV-2 genome and predictions of the human interactome | VIRUS CHARACTERIZATION | Interactome: Viral RNA and protein interactions | Vandelli A | Nucleic Acids Res | 2020 |
| **34** | PMID: 32780783 | Global cataloguing of variations in untranslated regions of viral genome and prediction of key host RNA binding protein microRNA interactions modulating genome stability in SARS-CoV-2 | VIRUS CHARACTERIZATION | Interactome: Viral RNA and protein interactions | Mukherjee M | PLoS One | 2020 |
| **35** | PMID: 32297156 | Computational Identification of Small Interfering RNA Targets in SARS-CoV-2 | VIRUS CHARACTERIZATION | Interactome: Viral RNA and protein interactions | Chen W | Virol Sin | 2020 |
| **36** | PMID: 28216251 | SARS-CoV-Encoded Small RNAs Contribute to Infection-Associated Lung Pathology | VIRUS CHARACTERIZATION | Interactome: Viral RNA and protein interactions | Morales L | Cell Host Microbe | 2020 |
| **37** | PMID: 33349665 | The SARS-CoV-2 RNA–protein interactome in infected human cells | VIRUS CHARACTERIZATION | Interactome: Viral RNA and protein interactions | Schmidt N | Nat Microbiol | 2021 |
| **38** | PMID: 33052334 | Systematic discovery and functional interrogation of SARS-CoV-2 viral RNA-host protein interactions during infection | VIRUS CHARACTERIZATION | Interactome: Viral RNA and protein interactions | Flynn RA | bioRxiv [Preprint]. | 2020 |
| **39** | PMID: 33147444 | Genome-wide CRISPR Screens Reveal Host Factors Critical for SARS-CoV-2 Infection | VIRUS CHARACTERIZATION | Interactome: Viral RNA and protein interactions | Wei J | Cell | 2021 |
| **40** | PMID: 33263384 | Comparative multiplexed interactomics of SARS-CoV-2 and homologous coronavirus non-structural proteins identifies unique and shared host-cell dependencies | VIRUS CHARACTERIZATION/Pathway | Interactome: Virus-host protein-protein interactions (PPI) | Davies JP | ACS Infect Dis | 2020 |
| **41** | PMID: 33103435 | Computational Identification of Human Biological Processes and Protein Sequence Motifs Putatively Targeted by SARS- CoV-2 Proteins Using Protein-Protein Interaction Networks | VIRUS CHARACTERIZATION/Pathway | Interactome: Virus-host protein-protein interactions (PPI) | Nadeau R | J Proteome Res | 2020 |
| **42** | PMID: 32811513 | The current landscape of coronavirus-host protein- protein interactions | VIRUS CHARACTERIZATION | Interactome: Virus-host protein-protein interactions (PPI) | Perrin-Cocon L | J Transl Med | 2020 |
| **43** | PMID: 32522207 | COVID-19: Viral-host interactome analyzed by network based-approach 1 model to study pathogenesis | VIRUS CHARACTERIZATION/Pathway | Interactome: Virus-host protein-protein interactions (PPI) | Messina F | J Transl Med | 2020 |
| **44** | PMID: 33206959 | The IMEx coronavirus interactome: an evolving map of Coronaviridae-host molecular interactions | VIRUS CHARACTERIZATION/Pathway | Interactome: Virus-host protein-protein interactions (PPI) | Perfetto L | Database | 2020 |
| **45** | PMID: 33357464 | Functional interrogation of a SARS-CoV-2 host protein interactome identifies unique and shared coronavirus host factors | VIRUS CHARACTERIZATION/Pathway | Interactome: multilayer (transcriptomics, proteomics) virus-host interactions | Hoffmann HH | Cell Host Microbe | 2021 |
| **46** | PMID: 33164753 | Single-cell multiomic profiling of human lungs reveals cell-type-specific and age-dynamic control of SARS-CoV-2 host genes | VIRUS CHARACTERIZATION | Interactome: multilayer (transcriptomics, proteomics) virus-host interactions | Wang A | Elife | 2020 |
| **47** | PMID: 32479746 | Host-Viral Infection Maps Reveal Signatures of Severe COVID-19 Patients | VIRUS CHARACTERIZATION | Interactome: multilayer (transcriptomics, proteomics) virus-host interactions | Bost P | Cell | 2020 |
| **48** | PMID: 32895641 | Integrative Network Biology Framework Elucidates Molecular Mechanisms of SARS-CoV-2 Pathogenesis | VIRUS CHARACTERIZATION/Phenotypes | Interactome: multilayer (transcriptomics, proteomics) virus-host interactions | Kumar N | iScience | 2020 |
| **49** | PMID: 32244779 | Master Regulator Analysis of the SARS-CoV-2/Human Interactome | VIRUS CHARACTERIZATION/Pathway | Interactome: multilayer (transcriptomics, proteomics) virus-host interactions | Guzzi PH | J Clin Med | 2020 |
| **50** | PMID: 32371892 | COVID-19 Disease Map, building a computational repository of SARS-CoV-2 virus-host interaction mechanisms | VIRUS CHARACTERIZATION | Interactome: multilayer (transcriptomics, proteomics) virus-host interactions | Ostaszebski M | Sci Data | 2020 |
| **51** | PMID: 32142651 | SARS-CoV-2 Cell Entry Depends on ACE2 and TMPRSS2 and Is Blocked by a Clinically Proven Protease Inhibitor. | VIRUS CHARACTERIZATION | Viral entry | Hoffmann M | Cell | 2020 April |
| **52** | PMID: 33082294 | Neuropilin-1 is a host factor for SARS-CoV-2 infection. | VIRUS CHARACTERIZATION | Viral entry | Daly JL | Science | 2020 Nov |
| **53** | PMID: 33082293 | Neuropilin-1 facilitates SARS-CoV-2 cell entry and infectivity | VIRUS CHARACTERIZATION | Viral entry | Cantuti-Castelvetri | Science | 2020 Nov |
| **54** | PMID: 33164751 | ACE2: Evidence of role as entry receptor for SARS-CoV-2 and implications in comorbidities. | VIRUS CHARACTERIZATION | Viral entry | Zamorano Cuervo N, | Elife | 2020 Nov |
| **55** | PMID: 32715618 | The protein expression profile of ACE2 in human tissues. | VIRUS CHARACTERIZATION | Viral entry | Hikmet F, | Mol Syst Biol | 2020 Jul |
| **56** | PMID: 32463365 | Knowledge synthesis of 100 million biomedical documents augments the deep expression profiling of coronavirus receptors. | VIRUS CHARACTERIZATION | Viral entry | Venkatakrishnan AJ, | Elife | 2020 May |
| **57** | PMID:  32669955 | A comprehensive investigation of the mRNA and protein level of ACE2, the putative receptor of SARS-CoV-2, in human tissues and blood cells. | VIRUS CHARACTERIZATION | Viral entry | Wang Y, | Int J Med Sci | 2020 Jun |
| **58** | PMID: 32662421 | Does the human placenta express the canonical cell entry mediators for SARS-CoV-2? | VIRUS CHARACTERIZATION | Viral entry | Pique-Regi R, | Elife | 2020 Jul |
| **59** | PMID: 32675206 | Gene expression and <i>in situ</i> protein profiling of candidate SARS-CoV-2 receptors in human airway epithelial cells and lung tissue. | VIRUS CHARACTERIZATION | Viral entry | Aguiar JA, | Eur Respir J | 2020 Sep |
| **60** | PMID: 32413319 | SARS-CoV-2 Receptor ACE2 Is an Interferon-Stimulated Gene in Human Airway Epithelial Cells and Is Detected in Specific Cell Subsets across Tissues. . | VIRUS CHARACTERIZATION | Viral entry | Ziegler CGK | Cell | 2020 May |
| **61** | PMID:  32703421 | Genetic variants that influence SARS-CoV-2 receptor TMPRSS2 expression among population cohorts from multiple continents. | VIRUS CHARACTERIZATION | Viral entry | Irham LM | Biochem Biophys Res Commun | 2020 Aug |
| **62** | PMID: 32410735 | Interaction of the spike protein RBD from SARS-CoV-2 with ACE2: Similarity with SARS-CoV, hot-spot analysis and effect of the receptor polymorphism. | VIRUS CHARACTERIZATION | Viral entry | Othman H, | Biochem Biophys Res Commun | 2020 Jun |
| **63** | PMID: 32946807 | A Single-Cell RNA Expression Map of Human Coronavirus Entry Factors. | VIRUS CHARACTERIZATION | Viral entry | Singh M, | Cell Rep | 2020 Sep |
| **64** | PMID: 15654751 | The aromatic domain of the coronavirus class I viral fusion protein induces membrane permeabilization: putative role during viral entry. | VIRUS CHARACTERIZATION | Viral entry | Sainz B Jr, | Biochemistry | 2005 Jan |
| **65** | PMID: 32817270 | In situ structural analysis of SARS-CoV-2 spike reveals flexibility mediated by three hinges. | VIRUS CHARACTERIZATION | Viral entry | Turonová B, | Science | 2020 Oct |
| **66** | PMID: 32075877 | Cryo-EM structure of the 2019-nCoV spike in the prefusion conformation. | VIRUS CHARACTERIZATION | Viral entry | Wrapp D, | Science | 2020 Mar |
| **67** | PMID: 32155444 | Structure, Function, and Antigenicity of the SARS-CoV-2 Spike Glycoprotein. | VIRUS CHARACTERIZATION | Viral entry | Walls AC, | Cell | 2020 Apr |
| **68** | PMID:  32790406 | Does SARS-CoV-2 Bind to Human ACE2 More Strongly Than Does SARS-CoV? | VIRUS CHARACTERIZATION | Viral entry | Nguyen | J Phys Chem B | 2020 Aug |
| **69** | PMID: 33556558 | SARS-CoV-2 mutation 614G creates an elastase cleavage site enhancing its spread in high AAT-deficient regions. | VIRUS CHARACTERIZATION | Viral entry | Bhattacharyya C | Infect Genet Evol | 2021 July |
| **70** | PMID:  32697968 | Tracking Changes in SARS-CoV-2 Spike: Evidence that D614G Increases Infectivity of the COVID-19 Virus. | VIRUS CHARACTERIZATION | Viral entry | Korber B, | Cell | 2020 Aug |
| **71** | PMID: 33581803 | SARS-CoV-2 variants and ending the COVID-19 pandemic. | VIRUS CHARACTERIZATION | Viral entry | Fontanet A, | Lancet | 2021 Mar |
| **72** | PMID: 33621484 | Circulating SARS-CoV-2 spike N439K variants maintain fitness while evading antibody-mediated immunity. | VIRUS CHARACTERIZATION | Viral entry | Thomson EC, | Cell | 2021 Mar |
| **73** | PMID: 32695025 | Regulatory Cross Talk Between SARS-CoV-2 Receptor Binding and Replication Machinery in the Human Host | Viral entry/ Pathways | IN SILICO VIRUS-HOST INTERACTOMES | Ahmed SSSJ | Front Physiol | 2020 |

### Table S2 B. Host signature.

| **ID TE** | **URL** | **Title** | **Domain/sub-domains** | **Investigation field** | **First Author (et al.)** | **Journal** | **Year** |
| --- | --- | --- | --- | --- | --- | --- | --- |
| **83** | PMID:  32838362 | Virus-Host Interactome and Proteomic Survey Reveal Potential Virulence Factors Influencing SARS-CoV-2 Pathogenesis | Pathways/Host signature | INTERACTOMICS | Li J | Med | 2021 |
| **87** | PMID:  32416070 | Imbalanced Host Response to SARS-CoV-2 Drives Development of COVID-19 | Host signature/Phenotypes | Host signature | Blanco-Melo | Cell | 2020 |
| **88** | PMID:  32788344 | Serum Protein Profiling Reveals a Landscape of Inflammation and Immune Signaling in Early-stage COVID-19 Infection | Pathways/Host signature/Phenotypes | Host signature | Hou X | Mol Cell Proteomics | 2020 |
| **89** | PMID:  32661059 | Impaired type I interferon activity and inflammatory responses in severe COVID-19 patients. | Host signature/Phenotypes | Host signature | Hadjadj | Science | 2020 |
| **90** | PMID:  33171100 | Multi-Omics Resolves a Sharp Disease-State Shift between Mild and Moderate COVID-19 | Pathways/Host signature | Host signature | [Su Y](https://pubmed.ncbi.nlm.nih.gov/?sort=date&term=Su+Y&cauthor_id=33171100) | Cell | 2020 |
| **91** | PMID:  33239683 | Longitudinal proteomic profiling reveals increased early inflammation and sustained apoptosis proteins in severe COVID-19 | Pathways/Host signature | Host signature | Haljasmägi L | Scientific Reports | 2020 |
| **92** | PMID:  32619549 | Ultra-High-Throughput Clinical Proteomics Reveals Classifiers of COVID-19 Infection | Pathways/Host signature | Host signature | Messner CB | Cell Systems | 2020 |
| **93** | PMID:  33128875 | Plasma Proteomics Identify Biomarkers and Pathogenesis of COVID-19 | Pathways/Host signature | Host signature | Shu Ting | Immunity | 2020 |
| **96** | PMID:  33140861 | Blood molecular markers associated with COVID-19 immunopathology and multi-organ damage | Pathways/Host signature/Phenotypes | Host signature | Chen YM | The EMBO Journal | 2020 |
| **98** | PMID:  33597532 | Dysregulated transcriptional responses to SARS-CoV-2 in the periphery | Host signature | Host signature | [McClain MT](https://pubmed.ncbi.nlm.nih.gov/?sort=date&term=McClain+MT&cauthor_id=33597532) | MedRxive/Nat Commun | 2020/2021 |
| **99** | PMID:  32747830 | Immune complement and coagulation dysfunction in adverse outcomes of SARS-CoV-2 infection | Host signature | Host signature | Ramlall V | Nat. Med | 2020 |
| **105** | PMID:  33296687 | Longitudinal Multi-omics Analyses Identify Responses of Megakaryocytes, Erythroid Cells, and Plasmablasts as Hallmarks of Severe COVID-19 | Host Signature | Host signature | Bernardes | Immunity | 2020 |
| **106** | PMID:  32670298 | Immune and Metabolic Signatures of COVID-19 Revealed by Transcriptomics Data Reuse | Host signature | Host signature | Gardinassi | Frontiers in Immunology | 2020 |
| **107** | PMID:  32599245 | Targeting hub genes and pathways of innate immune response in COVID-19: A network biology perspective | Host signature/Phenotypes | Host signature | Prasad K | International Journal of Biological Macromolecules | 2020 |
| **108** | PMID:  32407669 | Heightened Innate Immune Responses in the Respiratory Tract of COVID-19 Patients | Host signature/Phenotypes | Host signature | Zhou Z | Cell Host & Microbe | 2020 |
| **109** | PMID:  33060566 | Pathological features of COVID-19-associated lung injury: a preliminary proteomics report based on clinical samples | Host signature/Phenotypes | Host signature | Leng L | Signal Transduct Target Ther | 2020 |
| **110** | PMID:  33082228 | Transcriptional and proteomic insights into the host response in fatal COVID-19 cases | Host signature/Pathways/Phenotypes | Host signature | Wu M | Proc Natl Acad Sci U S A | 2020 |
| **111** | PMID:  32783921 | Single-Cell Sequencing of Peripheral Mononuclear Cells Reveals Distinct Immune Response Landscape of COVID-19 and Influenza Patients | Host signature/Phenotypes | Host signature | Zhu L | Immunity | 2020 |
| **112** | PMID:  32810438 | Severe COVID-19 Is Marked by a Dysregulated Myeloid Cell Compartment | Host signature/Phenotypes | Host signature | Schulte-Schrepping | Cell | 2020 |
| **113** | PMID:  33131070 | Novel insight from the first lung transplant of a COVID-19 patient | Host signature | Host signature | Chen XJ | Eur J Clin Invest. | 2021 |
| **114** | PMID:  33015591 | PPARγ Cistrome Repression during Activation of Lung Monocyte-Macrophages in Severe COVID-19. | Host signature/Phenotypes | Host signature | Desterke C | iScience | 2020 |

### Table S2 C. Pathways.

| **ID TE** | **URL** | **Title** | **Domain/sub-domains** | **Investigation field** | **First Author (et al.)** | **Journal** | **Year** |
| --- | --- | --- | --- | --- | --- | --- | --- |
| **73** | PMID:  32695025 | Regulatory Cross Talk Between SARS-CoV-2 Receptor Binding and Replication Machinery in the Human Host | Viral entry/ Pathways | IN SILICO VIRUS-HOST INTERACTOMES | Ahmed SSSJ | Front Physiol. | 2020 |
| **74** | PMID:  20392858 | Quantitative proteomics analysis reveals BAG3 as a potential target to suppress severe acute respiratory syndrome coronavirus replication | Pathways | PROTEOMICS ON IN VITRO INFECTIONS | Zhang L | J Virol | 2010 |
| **75** | PMID:  32691695 | Dysregulation in Akt/mTOR/HIF-1 signaling identified by proteo-transcriptomics of SARS-CoV-2 infected cells. | Pathways | TRANSCRIPTOMICS AND PROTEOMICS ON IN VITRO INFECTIONS | Appelberg S | Emerg Microbes Infect | 2020 |
| **76** | PMID:  32971089 | Integrative transcriptomics analysis of lung epithelial cells and identification of repurposable drug candidates for COVID-19 | Pathways | IN SILICO TRANSCRIPTOMICS | Islam T | Eur J Pharmacol | 2020 |
| **77** | PMID:  33060197 | Comparative host-coronavirus protein interaction networks reveal pan-viral disease mechanisms | Pathways | PROTEOMICS AND GENETIC SCREEN ON IN VITRO INFECTIONS | Gordon DE | Science | 2020 |
| **78** | PMID:  33259812 | Actionable Cytopathogenic Host Responses of Human Alveolar Type 2 Cells to SARS-CoV-2 | Pathways | PROTEOMICS ON IN VITRO INFECTIONS | Hekman RM | Mol Cell | 2020 |
| **79** | PMID:  32408336 | Proteomics of SARS-CoV-2-infected host cells reveals therapy targets. | Pathways | TRANSCRIPTOMICS AND PROTEOMICS ON IN VITRO INFECTIONS | Bojkova D | Nature | 2020 |
| **80** | PMID:  32645325 | The Global Phosphorylation Landscape of SARS-CoV-2 Infection | Pathways | PROTEOMICS ON IN VITRO INFECTIONS | Bouhaddou M | Cell | 2020 |
| **81** | PMID:  33845483 | Multi-level proteomics reveals host-perturbation strategies of SARS-CoV-2 and SARS-CoV | Pathways | PROTEOMIC AND INTERACTOMICS IN VITRO | Stukalov A | bioRxiv  Pre-print | 2020 |
| **82** | [PMID: 20478047](https://pubmed.ncbi.nlm.nih.gov/20478047/) | Interactions of SARS coronavirus nucleocapsid protein with the host cell proteasome subunit p42 | Pathways | INTERACTOMICS | Wang Q | Virol J | 2010 |
| **83** | [PMID: 32838362](https://pubmed.ncbi.nlm.nih.gov/32838362/) | Virus-Host Interactome and Proteomic Survey Reveal Potential Virulence Factors Influencing SARS-CoV-2 Pathogenesis | Pathways/host signature | INTERACTOMICS | Li J | Med | 2021 |
| **84** | [PMID: 32575076](https://pubmed.ncbi.nlm.nih.gov/32575076/) | Re-analysis of SARS-CoV-2-infected host cell proteomics time-course data by impact pathway analysis and network analysis: a potential link with inflammatory response | Pathways | IN SILICO PROTEOMIC | Bock JO | Aging | 2020 |
| **85** | [PMID: 32591346](https://pubmed.ncbi.nlm.nih.gov/32591346/) | Data, Reagents, Assays and Merits of Proteomics for SARS-CoV-2 Research and Testing. | Pathways | PROTEOMICS ON IN VITRO INFECTIONS | Zecha J | Mol Cell Proteomics | 2020 |
| **86** | [PMID: 32619390](https://pubmed.ncbi.nlm.nih.gov/32619390/) | Shotgun proteomics analysis of SARS-CoV-2-infected cells and how it can optimize whole viral particle antigen production for vaccines | Pathways | PROTEOMICS ON IN VITRO INFECTIONS | Grenga L | Emerg Microbes Infect | 2020 |
| **88** | PMID: 32788344 | Serum Protein Profiling Reveals a Landscape of Inflammation and Immune Signaling in Early-stage COVID-19 Infection | Pathways/Host signature/phenotypes | Host signature | Hou X | Mol Cell Proteomics | 2020 |
| **90** | PMID: 33171100 | Multi-Omics Resolves a Sharp Disease-State Shift between Mild and Moderate COVID-19 | Pathways/Host signature | Host signature | [Su Y](https://pubmed.ncbi.nlm.nih.gov/?sort=date&term=Su+Y&cauthor_id=33171100) | Cell | 2020 |
| **91** | PMID: 33239683 | Longitudinal proteomic profiling reveals increased early inflammation and sustained apoptosis proteins in severe COVID-19 | Pathways/Host signature | Host signature | Haljasmägi L | Scientific Reports | 2020 |
| **92** | [PMID: 32619549](https://pubmed.ncbi.nlm.nih.gov/32619549/) | Ultra-High-Throughput Clinical Proteomics Reveals Classifiers of COVID-19 Infection | Pathways/Host signature | Host signature | Messner CB | Cell Systems | 2020 |
| **93** | [PMID: 33128875](https://pubmed.ncbi.nlm.nih.gov/33128875/) | Plasma Proteomics Identify Biomarkers and Pathogenesis of COVID-19 | Pathways/Host signature | Host signature | Shu Ting | Immunity | 2020 |
| **94** | PMID: 32492406 | Proteomic and Metabolomic Characterization of COVID-19 Patient Sera | Pathways/Phenotypes | Plasma | Shen B | Cell | 2020 |
| **95** | PMID: 33096026 | Large-Scale Multi-omic Analysis of COVID-19 Severity | Pathways | Plasma | Overmyer KA | Cell Systems | 2021 |
| **96** | PMID: 33140861 | Blood molecular markers associated with COVID-19 immunopathology and multi-organ damage | Pathways/Host signature/phenotypes | Host signature | Chen YM | The EMBO Journal | 2020 |
| **97** | PMID: 32786691 | Serum Proteomics in COVID-19 Patients: Altered Coagulation and Complement Status as a Function of IL-6 Level | Pathways/Phenotypes | Plasma | D'Alessandro A | J Proteome Res. | 2020 |
| **100** | PMID: 33043283 | SARS-CoV-2 Infection Dysregulates the Metabolomic and Lipidomic Profiles of Serum | Pathways/Phenotypes | Plasma | Bruzzone C | iScience | 2020 |
| **101** | PMID: 33207699 | Large-Scale Plasma Analysis Revealed New Mechanisms and Molecules Associated with the Host Response to SARS-CoV-2 | Pathways/Phenotypes | Plasma | Barberis E | Int J Mol Sci. | 2020 |
| **102** | PMID: 32610096 | Omics-Driven Systems Interrogation of Metabolic Dysregulation in COVID-19 Pathogenesis | Pathways | Plasma | Song J-W | Cell Metabolism | 2020 |
| **103** | PMID: 33103907 | Evidence of Structural Protein Damage and Membrane Lipid Remodeling in Red Blood Cells from COVID-19 Patients | Pathways | Plasma | Thomas T | J Proteome Res. | 2020 |
| **104** | PMID: 32806897 | Integrative Modeling of Quantitative Plasma Lipoprotein, Metabolic, and Amino Acid Data Reveals a Multiorgan Pathological Signature of SARS-CoV-2 Infection | Pathways | Plasma | Kimhofer T | J Proteome Res. | 2020 |
| **110** | PMID: 33082228 | Transcriptional and proteomic insights into the host response in fatal COVID-19 cases | Host signature/Pathways/Phenotypes | Host signature | Wu M | Proc Natl Acad Sci U S A | 2020 |
| **146** | PMID: 32559180 | COVID-19 infection alters kynurenine and fatty acid metabolism, correlating with IL-6 levels and renal status | Pathways/Phenotypes | Plasma | Thomas T | JCI Insight. | 2020 |
| **147** | PMID: 32073213 | Abnormal coagulation parameters are associated with poor prognosis in patients with novel coronavirus pneumonia. | Pathways |  | Tang N, | J Thromb Haemost | 2020 |
| **148** | PMID: 32306492 | D-dimer levels on admission to predict in-hospital mortality in patients with COVID-19. | Pathways |  | Zhang L, | J Thromb Haemost | 2020 |
| **149** | PMID: 32526193 | Pulmonary post-mortem findings in a series of COVID-19 cases from northern Italy: a two-centre descriptive study. | Pathways |  | Carsana L | Lancet Infect Dis. | 2020 Oct |
| **150** | PMID: 32437596 | Pulmonary Vascular Endothelialitis, Thrombosis, and Angiogenesis in COVID-19. | Pathways |  | Ackermann | N Engl J Med. | 2020 Jul |
| **151** | PMID: 32685880 | A proposal for staging COVID-19 coagulopathy. | Pathways |  | Thachil J | Res Pract Thromb Haemost. | 2020 Jul |

### Table S2 D. Phenotypes.

| **ID TE** | **URL** | **Title** | **Domain/sub-domains** | **Investigation field** | **First Author (et al.)** | **Journal** | **Year** |
| --- | --- | --- | --- | --- | --- | --- | --- |
| **48** | PMID: 32895641 | Integrative Network Biology Framework Elucidates Molecular Mechanisms of SARS-CoV-2 Pathogenesis | VIRUS CHARACTERIZATION/Phenotypes | Interactome: multilayer (transcriptomics, proteomics) virus-host interactions | Kumar N | iScience | 2020 |
| **87** | PMID: 32416070 | Imbalanced Host Response to SARS-CoV-2 Drives Development of COVID-19 | Host signature/Phenotypes | Host signature | Blanco-Melo | Cell | 2020 |
| **88** | PMID: 32788344 | Serum Protein Profiling Reveals a Landscape of Inflammation and Immune Signaling in Early-stage COVID-19 Infection | Pathways/Host signature/phenotypes | Host signature | Hou X | Mol Cell Proteomics | 2021 |
| **89** | PMID: 32661059 | Impaired type I interferon activity and inflammatory responses in severe COVID-19 patients. | Host signature/Phenotypes | Host signature | Hadjadj | Science | 2020 |
| **94** | PMID: 32492406 | Proteomic and Metabolomic Characterization of COVID-19 Patient Sera | Pathways/Phenotypes | Plasma | Shen B | Cell | 2020 |
| **96** | PMID: 33140861 | Blood molecular markers associated with COVID-19 immunopathology and multi-organ damage | Pathways/Host signature/phenotypes | Host signature | Chen YM | The EMBO Journal | 2020 |
| **97** | PMID: 32786691 | Serum Proteomics in COVID-19 Patients: Altered Coagulation and Complement Status as a Function of IL-6 Level | Pathways/Phenotypes | Plasma | D'Alessandro A | J Proteome Res. | 2020 |
| **100** | PMID: 33043283 | SARS-CoV-2 Infection Dysregulates the Metabolomic and Lipidomic Profiles of Serum | Pathways/Phenotypes | Plasma | Bruzzone C | iScience | 2020 |
| **101** | PMID: 33207699 | Large-Scale Plasma Analysis Revealed New Mechanisms and Molecules Associated with the Host Response to SARS-CoV-2 | Pathways/Phenotypes | Plasma | Barberis E | Int J Mol Sci. | 2020 |
| **107** | PMID: 32599245 | Targeting hub genes and pathways of innate immune response in COVID-19: A network biology perspective | Host signature/Phenotypes | Host signature | Prasad K | International Journal of Biological Macromolecules | 2020 |
| **108** | PMID: 32407669 | Heightened Innate Immune Responses in the Respiratory Tract of COVID-19 Patients | Host signature/Phenotypes | Host signature | Zhou Z | Cell Host & Microbe | 2021 |
| **109** | PMID: 33060566 | Pathological features of COVID-19-associated lung injury: a preliminary proteomics report based on clinical samples | Host signature/Phenotypes | Host signature | Leng L | Signal Transduct Target Ther | 2020 |
| **110** | PMID: 33082228 | Transcriptional and proteomic insights into the host response in fatal COVID-19 cases | Host signature/Pathways/Phenotypes | Host signature | Wu M | Proc Natl Acad Sci U S A | 2020 Dec |
| **111** | PMID: 32783921 | Single-Cell Sequencing of Peripheral Mononuclear Cells Reveals Distinct Immune Response Landscape of COVID-19 and Influenza Patients | Host signature/Phenotypes | Host signature | Zhu L | Immunity | 2020 Oct |
| **112** | PMID: 32810438 | Severe COVID-19 Is Marked by a Dysregulated Myeloid Cell Compartment | Host signature/Phenotypes | Host signature | Schulte-Schrepping | Cell | 2020 |
| **114** | PMID: 33015591 | PPARγ Cistrome Repression during Activation of Lung Monocyte-Macrophages in Severe COVID-19. | Host signature/Phenotypes | Host signature | Desterke C | iScience | 2020 |
| **115** | PMID: 33180746 | Age-determined expression of priming protease TMPRSS2 and localization of SARS-CoV-2 in lung epithelium | Phenotypes | Severity profiles | Schuler BA | J Clin Invest | 2020 |
| **116** | PMID: 32497778 | A pharmacological interactome between COVID-19 patient samples and human sensory neurons reveals potential drivers of neurogenic pulmonary dysfunction. | Phenotypes | Severity profiles | Ray PR, | Brain Behav Immun. | 2020 |
| **117** | PMID: 33354650 | Host–Viral Interactions Revealed among Shared Transcriptomics Signatures of ARDS and Thrombosis: A Clue into COVID-19 Pathogenesis | Phenotypes | Severity Profiles | Mishra A | TH Open | 2021 Jan |
| **118** | PMID: 33098359 | Proteomic characteristics of bronchoalveolar lavage fluid in critical COVID-19 patients. | Phenotypes | Severity Profiles | Zeng HL, | FEBS J. | 2020 Oct |
| **119** | PMID: 32526012 | ACE2 Expression Is Increased in the Lungs of Patients With Comorbidities Associated With Severe COVID-19. | Phenotypes | Severity Profiles | Pinto BGG | J Infect Dis | 2020 Oct |
| **120** | PMID: 33521749 | Cell-free DNA tissues of origin by methylation profiling reveals significant cell, tissue, and organ-specific injury related to COVID-19 severity | Phenotypes | Severity Profiles | Cheng AP | Med (N Y). | 2020 Aug |
| **121** | PMID: 32915959 | Intestinal Receptor of SARS-CoV-2 in Inflamed IBD Tissue Seems Downregulated by HNF4A in Ileum and Upregulated by Interferon Regulating Factors in Colon | Phenotypes | Localisation | Verstockt B | Journal of Crohn's and Colitis | 2020 Oct |
| **122** | PMID: 32622411 | Coronavirus disease-19 and fertility: viral host entry protein expression in male and female reproductive tissues. | Phenotypes | Localisation | Stanley KE | Epub | 2020 Oct |
| **123** | PMID: 33049061 | Co-Expression of Mitochondrial Genes and ACE2 in Cornea Involved in COVID-19 | Phenotypes | Localisation | Yuan J, | Invest Ophthalmol Vis Sci. | 2020 |
| **124** | PMID: 33382973 | Neurological Manifestations of COVID-19 Feature T Cell Exhaustion and Dedifferentiated Monocytes in Cerebrospinal Fluid | Phenotypes | Localisation | Heming M. | Immunity. | 2020 Dec |
| **125** | PMID: 32975064 | Quantitative Proteomic Analysis of the Expression of SARS-CoV-2 Receptors in the Gut of Patients with Chronic Enterocolitis | Phenotypes | Localisation | Park J | Yonsei Med J. | 2020 |
| **126** | PMID: 32598884 | Alterations in Fecal Fungal Microbiome of Patients With COVID-19 During Time of Hospitalization until Discharge. | Phenotypes | Localisation | Zuo T | Gastroenterology | 2020 |
| **127** | PMID: 32641214 | SARS-CoV-2 infection risk assessment in the endometrium: viral infection-related gene expression across the menstrual cycle | Phenotypes | Localisation | Henarejos-Castillo | Fertility and Sterility | 2020 Nov |
| **128** | PMID: 33011738 | Identification and validation of predictive factors for progression to severe COVID-19 pneumonia by proteomics. | Phenotypes | severity profiles | Di B | Signal Transduct Target Ther | 2020 Nov |
| **129** | PMID: 33097684 | Omics study reveals abnormal alterations of breastmilk proteins and metabolites in puerperant women with COVID-19 | Phenotypes | Localisation | Zhao Y | Signal Transduct Target Ther. | 2020 Nov |
| **130** | PMID: 33092637 | Human genetic factors associated with susceptibility to SARS-CoV-2 infection and COVID-19 disease severity | Phenotypes | severity profiles | Anastassopoulou C | Hum Genomics | 2021 March |
| **131** | PMID: 33425248 | Host transcriptomic profiling of COVID-19 patients with mild, moderate, and severe clinical outcomes | Phenotypes | Severity Profiles | Jain R | Comput Struct Biotechnol J. | 2020 Dec |
| **132** | PMID: 32788292 | Systems biological assessment of immunity to mild versus severe COVID-19 infection in humans. | Phenotypes | Severity profiles | Arunachalam PS | Science | 2020 |
| **133** | PMID: 32780218 | A human circulating immune cell landscape in aging and COVID-19. | Phenotypes | Severity profiles | Zheng Y | Protein Cell. | 2021 Feb |
| **134** | PMID: 33298875 | Initial whole-genome sequencing and analysis of the host genetic contribution to COVID-19 severity and susceptibility | Phenotypes | Severity Profiles | Wang F | Cell Discov. | 2020 Dec |
| **135** | PMID: 33203833 | Immune suppression in the early stage of COVID-19 disease | Phenotypes | severity profiles | Tian W | Nat Commun. | 2020 Sep |
| **136** | PMID: 32981365 | Clinical and Proteomic Correlates of Plasma ACE2 (Angiotensin-Converting Enzyme 2) in Human Heart Failure | Phenotypes | severity profiles | Chirinos JA | Hypertension | 2020 Oct |
| **137** | PMID: 33558857 | Proteomic investigation reveals dominant alterations of neutrophil degranulation and mRNA translation pathways in patients with COVID-19 | Phenotypes | Severity Profiles | Bankar R. | iScience. | 2017 Aug |
| **138** | PMID: 33376242 | In‑depth blood proteome profiling analysis revealed distinct functional characteristics of plasma proteins between severe and non‑severe COVID‑19 patients | Phenotypes | Severity Profiles | Park J | Sci Rep | 2021 Feb |
| **139** | PMID: 32448590 | Marked Up-Regulation of ACE2 in Hearts of Patients With Obstructive Hypertrophic Cardiomyopathy: Implications for SARS-CoV-2-Mediated COVID-19. | Phenotypes | severity profiles and localization | Bos JM | Mayo Clin Proc | 2020 |
| **140** | PMID: 33160965 | Altered Intestinal ACE2 Levels Are Associated With Inflammation, Severe Disease, and Response to Anti-Cytokine Therapy in Inflammatory Bowel Disease | Phenotypes |  | Potdar AA | Gastroenterology. | 2020 |
| **141** | PMID: 33038424 | SARS-CoV-2 receptor networks in diabetic and COVID-19-associated kidney disease. | Phenotypes |  | Menon R, | Kidney Int. | 2020 |
| **142** | PMID: 32669391 | ARS-CoV-2 Infections and ACE2: Clinical Outcomes Linked With Increased Morbidity and Mortality in Individuals With Diabetes. | Phenotypes |  | Obukhov AG | Diabetes | 2020 Oct |
| **143** | PMID: 32833058 | Various Facets of Pathogenic Lipids in Infectious Diseases: Exploring Virulent Lipid-Host Interactome and Their Druggability. | Phenotypes |  | Dadhich R | J Membr Biol. | 2020 Jul |
| **144** | PMID: 28831119 | Altered Lipid Metabolism in Recovered SARS Patients Twelve Years after Infection | Phenotypes |  | Wu Q | Sci Rep | 2020 Jul |
| **145** | PMID: 33596592 | Plasma metabolomic profiling of patients recovered from COVID-19 with pulmonary sequelae 3 months after discharge | Phenotypes | Severity Profiles | Xu J | Clin Infect Dis | 2021 |
| **146** | PMID: 32559180 | COVID-19 infection alters kynurenine and fatty acid metabolism, correlating with IL-6 levels and renal status | Pathways/Phenotypes | Plasma | Thomas T | JCI Insight. | 2021 |

## **Table S3 A. Viral characterization - Conceptual table (Additional file 2).**

## **Table S3 B. SARS-CoV-2 entry – Conceptual table.**

| **Subdomains** | **Cellular transcriptomics** | **Cellular proteomics** | **Cellular genomics** | **Viral genomics** |
| --- | --- | --- | --- | --- |
| **Viral receptors expression, tropism** | 1) Single cells and bulk RNAseq for ACE2 expression profile:  -high: intestinal tract, kidney, testis, gallbladder, heart, and the eye.  -Low: thyroid gland and adipose tissue,  -Very low: liver, female reproductive organs, nasal mucosa, and lung,  -No expression: Brain, lymphoid tissues, skin, smooth muscle, and immune cells.  2) Type II pneumocytes, nasal goblet secretory cells, and ileal absorptive enterocytes are ACE2+/TMPRSS2+  3) single-cell transcriptome profiling of the RNA levels of 28 genes named “SCARFs”, for SARS-CoV-2 and coronavirus-associated receptors and factors, suggests that intestine, kidneys, placenta, and spermatogonia are most permissive for coronaviruses | 1) IHC on ACE2 expression profile:  -high/medium: Small intestine duodenum and colon enterocytes and crypt cells, Kidney - proximal tubule cells,  Testis - Sertoli cells and Leydig cells, Gallbladder - glandular cells, Eye – conjunctiva, Seminal vesicle, Pancreas - interlobular ducts  -Low: heart, thyroid gland,  liver, epididymis, placenta, eye cornea  - No expression: nasal mucosa, bronchus, and lung; adipose tissue, brain, lymphoid tissues, skin, smooth muscle, and immune cells.  2) TMPRSS2, CD147 and GRP78 are expressed in airway epithelial cell lines and in lung tissues | *TMPRSS2* AA rs469390, GG rs2070788, TT rs383510, TT rs464397 genotypes are associated with the higher expression in the lung and lower frequencies in Asian compared to European, and American populations |  |
| **Structure of viral proteins involved in virus entry** |  |  |  | 1) One site change at position 23403 in the Wuhan reference strain leading to Aspartate to Glycine mutation in *S* gene residue 614 (D614G) emerged in March 2020 and soon spread and became prevalent worldwide, showing potentially higher viral loads in COVID-19 patients, no changes in disease severity, nor in neutralizing activity of convalescent sera.  2) Cleavage sites in Spike protein of 614G subtypes can be linked to the prevalent spread of this variant in European and North America population.  3) VOC B.1.351, P1 lineages, and B.1.1.7 show: K417N, E484K, and N501Y “escape mutations” in the S protein which alter interaction with hACE2, greater transmissibility, and reduced neutralization activity (B.1.351 and P1 lineages)  4) Other immune escape mutations can emerge independently in multiple lineages circulating worldwide (i.e., N439K) |

## 8. **Table S4. Immune response to SARS-CoV-2 infection in lung and other tissues (A), peripheral blood (B) and specific cell types in blood immune cells (C). Pathways, host signature (differentially expressed genes and proteins) and body districts, subset per specific omics data: host proteomics, bulk RNAseq and scRNAseq.**

**A. Lung and other tissues**

|  | **Localization** | **Gene/Protein** | **Modulation** | **Groups comparison** |
| --- | --- | --- | --- | --- |
| **PROTEOMIC** | lung | iNOS, IL1β and IL6 , S100 A8, A9, A11, A12, and P, SARS-CoV-2 entry-associated protease cathepsins B and L | **up** | fatal COVID-19 vs controls |
| **BULK RNAseq** | nasopharyngeal (NP) swabs | ‘Inflammatory response’ genes, ‘interferon_alpha_response’, and ‘IL6/JAK/STAT3 signaling. | **up** | COVID-19 vs HD |
|  |  | complement cascade (Serpine1, C1Q, C4B, C4A, C2, C3, and others) |  |  |
|  | BAL | IL1B, CXCL17, CXCL8,CXCL1, CXCL2, CCL2, CCL7 and CXCR2, ISG | **up** |  |
|  |  | IL1RN and SOCS3, calgranulin and ISG |  |  |
|  |  | neutr/lympho |  |  |
|  |  | NCKAP1L, DOCK2, SPN, DOCK10 | **down** |  |
|  | nasopharyngeal (NP) swabs | C8G, FCN1 | **up** | significance with SARS-CoV-2 viral load |
|  | lung | neutrophil activation, generation of neutrophil extracellular traps (myeloperoxidase, lactoferrin, and histones) | **up** | fatal cases vs control |
|  | colon | genes related to the response to the TGF-β | **up** |  |
| **scRNAseq/CyTOFF** | BAL | members of the PPAR complex as EP300 RXRA RARA SUMO1 NR3C1 CCDC88A in CD14+/CD16+ cells | **down** | severe |
|  | colon | genes involved in immune cell activation and immunity | **down** | fatal vs control |

**B. Peripheral blood**

|  | **Gene/Protein** | **Modulatio**n | **Groups comparison** |
| --- | --- | --- | --- |
| **PROTEOMICS**  **Soluble mediators** | Pro-inflammatory cytokines (e.g., CXCL6) and proteins associated with immune cell activation (e.g., CD244 and CD40), cytokines and chemokines IL-6, IL1RA, CCL2, CCL8, CXCL2, CXCL8, CXCL9, CXCL16, complement factors, coagulation system, inflammation modulators, and upstream and downstream pro-inflammatory factors of IL6.  Factors: A1BG, ACTB;ACTG1, ALB, APOA1, APOC1, C1R, C1S, C8A, CD14, CFB, CFH, CFI [complement factor I], CRP, FGA, FGB,  FGG [fibrinogen alpha, beta, and gamma chains], GSN, HP,  ITIH3 [inter-alpha-trypsin inhibitor heavy chain 3],  ITIH4 [interalpha-trypsin inhibitor heavy chain 4],  LBP, LGALS3BP, LRG1,  SAA1 [serum amyloid A1], SAA1; SAA2 [serum amyloid A1 and A2 protein group],  SERPINA10, TF,  platelet degranulation (TAGLN2, PPBP, FGG, FGA, TLN1, FGB,ORM2, ORM1, THBS1, PF4, APOH, VWF, Serpin A3, ALDOA, TIMP1, PLG, ITIH3, KNG1;F13A1, Serpin F2, SPP2, FN1, CLEC3B, CD109, A2M, Serpin A4, APOA1)  the complement and coagulation cascades (FGG, FGA, FGB, CFI, VWF, F9, CFH, C4BPB, C9, VTN, PLG, KNG1, F13A1, Serpin F2, C4B, Serpin A5, Serpin D1, PROC, A2M),  CASP8, IFNγ, IL-18R and CCL8  Proteins with neutrophil functions: PRTN3, LCN2, CD24, BPI, CTSG, DEFA1, DEFA4, MMP8, and MPO  Thrombosis-related proteins: calprotectin, ferritin, and histone H3.  Enzymes with antimicrobial activity: CST3, DEFA1, LRG1, LYZC  Proteins involved in blood vessel damage response: Angiotensinogen, Fibulin-5, Neuropilin-1, Neuropilin-2 | up | COVID-19 vs HD |
|  |  |  |  |
|  |  |  |  |
|  | FETUB, CETP, and PI16 | down |  |
|  | CCL7, IL10, and IL6, S100A9, CRP, AZGP1, CFI, SERPINA3/ACT, and LCP1/LPL, IL-6, CXCL10, CXCL11, IFNγ, IL10, and monocyte-attracting CCL2, CCL7, CCL8, CASP8, TNFSF14, HGF, and TGFB1, OSM and S100A12, Neutrophil surface receptors (CXCR1, Siglec5, and CD177) and Neutrophil granule contents (DEFA1), IL8 and IL6 in PMBCs  Acute-phase proteins: SAA1, SAA2, SAA4, CRP, SAP/APCS, S100A9, AZGP1, Glyc A and Glyc B, AZGP1;  C6 and CFB. CFP and CPN1  FV, FVII, FX, fibrinogen alpha and beta, SerpinF2 and SERPINA1, SERPINA3, vitamin K dependent protein S, LCP1/LPL  Apoptosis-associated proteins CASP8, TNFSF14, HGF, and TGFB1 | up | severe vs mild |
|  | IFNa, IFNb  APOA1, APOA2, APOH, APOL1, APOL6, APOD and APOM  FETUB (fatty acid metabolism and inflammation inhibition)  CETP (promoter of lipid transfer between lipoproteins)  PI16 (Inhibitor of the chemokine Chemerin)  Gelsolin (extracellular actin scavenger)  Coagulation factors: prothrombin (F2) and thrombin-activation factor XIII (F13A1 and F13B), Plasminogen, SERPIND1, Protein C and SERPINA5 (Protein C inhibitor)  Factors involved in platelet degranulation: GPLD1 and CLEC3B | down |  |
|  | IFNG, IL6, CXCL8, CXCL10, and CCL2 | up | mild vs severe |
|  | S100 A8, A9, A11, A12, and P | up | fatal vs control |
|  | F13A1 | down | poor prognosis |
|  | D-dimer and fibrinogen degradation products | up |  |
|  | IL20, CCL27, IL21, IL6, IL17, CCL2, CXCL10 | up | early stage COVID-19 vs influenza |
|  | PLG | down |  |
|  | IFNG, IL6, CXCL8, CXCL10, and CCL2 | up | COVID-19 vs influenza |
|  | C1R, and C7 | down |  |
| **BULK RNAseq** | neutrophils and chemokines, IL-1β-related genes and vasodilatory signaling genes, IRF1, STAT3 | up | COVID-19 vs HD |
|  | Type I and III IFNs, ISG | down |  |
|  | Genes related to blood coagulation in PBMC, IL12 and IFN-response activation,  neutrophil activation, degranulation, and translation initiation, IFN signaling,  neutrophil chemotaxis, activation, and migration markers,  genes encoding molecules associated with neutrophil extracellular traps (AZU1, DEFA3, MPO, PRTN3, CTSG) and  ARG1, BATF, IL-6, IL-8, and IL-10 | up | severe vs mild |
|  | negative innate immune regulators (e.g. TRIM59, USP21, and NLRC3),  IRF1, STAT3, CD4, CD8a, CD8b, CD2, CD5, and CD7, HLA-DRA,  TCR signaling kinases and adaptors LCK, FYN, SKAP1, CARD11, CAMK4, and BTN3A1, and T cell differentiation and survival (BCL11B) | down |  |
|  | IFN-a | up | mild vs hd |
|  | IL13 and IFNG, p38 MAPK activation | up | fatal vs not fatal |
|  | KLKB1, FXII, the plasminogen activator inhibitor (SERPINE1),  RSAD2, IFIT2, CCL2, LAMP3 | up | early moderate COVID-19 vs seasonal coronavirus, influenza or bacterial pneumonia |
|  | PROS, LY6E, OASL, IFI27, IFI6, IL1, JAK/STAT, IL6, and IL10 signaling pathways | down |  |

**C. Specific cell types in blood immune cells**

|  | **Gene/Protein** | **Modulation** | **Groups**  **comparison** |
| --- | --- | --- | --- |
| **Innate cell compartment** | Non-classical monocytes, ISG15, IFI44L, MX1, X-linked inhibitor of apoptosis (XIAP)-associated factor 1 (XAF1) in NK, and DC cell. | **up** | COVID-19 vs HD |
|  | HLA-DR in myeloid cells, IL6 and TNF in mDC upon TLR stimulation, IFN-a in pDC, TRADD in NK cells | **down** |  |
|  | neutrophils, CD64 and PD-L1 on immature and the mature neutrophils, alarmins S100A8/9/12, relative percentage of natural killer (NK) cells, LDN expressing SMPO, ELANE, and PRTN3 (neutrophil extracellular trap formation), LDN with immature phenotype, including pro- and pre-neutrophils expressing S100A12, S100A9, MMP8, ARG1, and OLFM4.classical monocyte (CD14highCD16low) expressing inflammation-related transcripts such as S100A4, S100A9, S100A12, and decreased HLA-DR surface protein, polyfunctional monocytes | **up** | severe vs mild |
|  | HLA-DR, marker genes indicative of anti-inflammatory functions (e.g., CD163 and PLAC8) in monocytes. Non-classical monocyte fraction (CD14lowCD16high) expressing FCGR3A, CX3CR1, CD56bright subpopulation expressing IL7R, CD27, and CD62L, HLA-DR in myeloid cells.IL6 and TNF in mDC upon TLR stimulation. IFN-a in pDC | **down** |  |
|  | expression of CD226 and CD69 in classical monocytes | **up** | severe vs HD |
|  | pDC | **down** |  |
| **adaptive cell compartment** | ISG15, IFI44L, MX1, X-linked inhibitor of apoptosis (XIAP)-associated factor 1 (XAF1) in T, B cells. TNFSF10 (TRAIL) and its receptor TNFRSF10A, TNFRSF1B. TNFSF10 and FADD and FAS in B cells, Effector-like cells, CD4 polyfunctionality, in particular Th1 cytokine IFN-g, Th17 cytokines IL17-A, IL17-F, Th2 cytokine IL-4, and cytotoxic molecule granzyme B | **up** | COVID-19 vs HD |
|  | CD4+ and CD8+ T, cD38+, HLA-DR+ activated T cells. in CD8 T cells naive-related mRNA and protein markers LEF1, TCF7, and CD197, CD4 naive-like T cells (TCF7, CCR7, and CD197), Total lymphocytes and T cells | **down** |  |
|  | plasmablasts/ plasma, negative T-cell signaling, naive CD8 T cells, IGHA1, IGHG1, IgA+, and IgG+ in memory B cells and plasmablast, LTF, A proliferative exhausted CD8+ T cell expressing LAG3, TIGIT, and CD279, proliferation markers (MKI67 and TYMS), high cytotoxic signatures, and not fully lost its naïve signature. Set of TCRs in CD8 T cells with cytotoxic effector phenotype. activated naive B cells (downregulation of FCER2 and upregulation of SLAMF7) and antibody-secreting cells (ASCs) was observed in samples | **up** | severe vs mild |
|  | HLA-DR, CD8 + T cells, TCR diversity, effector CD8 T cells, CXCR5 and CCR6 in B cells | **down** |  |
|  | relative percentage of lymphocytes, including CD4+ T cells, and CD8+ T cells | **up** | relative to increasing severity |
|  | set of TCRs in CD8 T cells with memory-like phenotype | **up** | mild vs severe |
|  | cytotoxic effector CD8 T cells (GZMH, KLRD1, SLC9A3R1) | **up** | patients who improved |
|  | CD8 Polyfunctionality, in particular granzymeB and perforin, CD4 T cells expressing cytotoxic transcripts PRF1 and GNLY; CD4 T cells expressing exhaustion markers, elevated Th1 signature, elevated proliferation signature | **up** | moderate vs mild, severe, HD |
|  | pd1, cd19 | **up** | severe vs HD |
|  | OLAH | **up** | poor prognosis |
|  | CD3E | **down** |  |
|  | IFNG and GZMB in T cells | **up** | survivors vs fatal |
|  | CD4 T-cell activation | **down** |  |
|  | IL-17 signaling | **up** | sever-fatal vs severe-survivor |
|  | early B-cell activation, antibody production (IGHG1, IGHV2-5, IGHV3.30, IGLV3-19, IGLV3-25, and others) | **up** | COVID-19 vs seasonal coronavirus, influenza or bacterial pneumonia |

## 9. **Table S5. Pathogenic mechanisms in COVID-19 phenotype: SARS-CoV-2 - host interactions in the lung. (A), DEG and DEP analysis in other organs and tissues (B) Hub genes and pathway of innate immune response (C), Comorbidities COVID-19 associated not sharing COVID-19 pathogenesis (D), Comorbidities associated and related to COVID-19 pathway (E). Omics and pathway involved were split by different phenotypes: severe, mild/asymptomatic and other Infections.**

**A. SARS-CoV-2 - host interactions in the lung**

|  | **Transcriptomics** | **Proteomics** | **Genomics** |
| --- | --- | --- | --- |
| **COVID-19** | **SEVERE**:  In ciliated cells and type I alveolar epithelial cells (AT1 ), IFN stimulated genes (ISGs) and ACE2 gene highly expressed TMPRSS2 and SARS-CoV-2 RNA is highest  many genes contribute to generate neutrophil extracellular traps (NETs),  -Upregulation of proinflammatory cytokine and chemokine genes (IL-1B, CXCL17, CXCL8, and CCL2), typical antiviral ISGs (IFIT and IFITM family genes).  Increased cytokine signaling causing a “cytokine storm”, hypoxia (HIF1A, HLF), and inflammasome and sepsis-related genes (IL1R1/2, IL5RA, IL33, IL31RA).  CD48 is associated with increased disease severity but CD40 is not.  In BALF at the single-cell level, expression of the lipopolysaccharide (LPS) sensors TLR2 and TLR4 is induced: TLR2 expression increases with disease severity.    HLA-B is highly expressed in all lymphoid and myeloid cell subpopulations in the lungs of the severe COVID-19. | **SEVERE**:  Up-regulated DEPs in BALF: immunoglobulins, DEPs belonged to proinflammatory extracellular factors (Cytokines),  Increase of surface antigens (lymphocyte antigen 6D/E48 antigen (LY6D), CD9 antigen, CD177 antigen) and prostate stem cell antigen (PSCA). | **SEVERE**:  A multi-gene cluster on chromosome 3 significantly associated with respiratory failure. *LZTFL1*(strongly expressed in lung ) and *SLC6A20* (encodes for the sodium-imino acid (proline) are located within these loci, functionally interacting with *ACE2* |
|  |  | **MILD/ASYMPTOMATIC:** In both mild and severe COVID-19 reported the elevation of CCL2, a chemokine that attracts monocytes, memory T lymphocytes, and basophils. |  |
| **Other Infections** |  | Several DEPs between COVID-19 and influenza. High levels of IFNG, IL6, CXCL8, CXCL10 and CCL2 were reported in mild and severe COVID-19 cases |  |

**B. DEG and DEP analysis in other organs and tissues**

|  | **Transcriptomics** | **Proteomics** | **Metabolomics/**  **lipidomics** |
| --- | --- | --- | --- |
| **COVID-19** | **SEVERE**:  -ACE2 overexpression regulates mitochondria-localized NADPH oxidase 4, involved in Reactive Oxygen Species (ROS) production, indicated as a major player in COVID-19 pathogenesis and severity.  -Although there is not yet direct evidence that altered expression of intestinal ACE2 directly affects SARS-CoV-2 intestinal entry and tropisms to different intestinal sites, pro-inflammatory cytokines can upregulate colonic epithelial ACE2 expression in IBD patients,.  -ACE2 exhibits the highest co-expression correlation with TMPRSS2, SLC6A20C, and LZTFL1 in the cornea compared to the lungs and retinal pigment epithelium (RPE) suggesting that the cornea may provide a susceptibility for SARS-CoV-2 entry. | **SEVERE**:  On serum and BALF several specific proteins, including lung proteins, peripheral blood cellular proteins (CARD9, CD177, CHI3L1, PODXL, S100A12 and TNC) and plasmatic proteins (CP, CTNNA3, LY6D, and PSCA), TNC and KL-6 were significantly higher.  Serum amyloid A-2 protein (SAA2) and alpha-1-antichymotrypsin are predictive factors of potential progression from moderate to severe forms.  In severe patients, there was a significant suppression of lymphocyte and monocyte count.  Viral entry of SARS-CoV-2 into the host cell is not facilitated by gut inflammation associated with chronic colitis  Data suggest that the endometrium of older women undergoing ART is at higher risk of viral infection.  A multi-omics approach on peripheral blood and plasma samples showed a decreasing of brain-enhanced proteins regulating neurotransmitter synthesis (GLS, OGDH, DLD, etc.), neurotransmitter transport (GLUL, GLUD2, GLUD1), neurotransmitter receptors (HTRA3) | **SEVERE:**  In serum of severe COVID-19, patients it was found increased level of CRP and AST, down-regulation of multiple apolipoproteins (APOA1, APOA2, APOH, APOL1, APOD, and APOM) associated with macrophage functions.  Downregulation of liver-enhanced proteins regulating the transportation of sterol and cholesterol  . Phenylalanine and its decarboxylation product phenethylamine intertwine with more immune-mediated DEPs. The analysis identified also lipids with significant changes between the two groups of women.  Steroid hormones and bile acid derivatives  21-hydroxypregnenolone (essential intermediate for Corticosterone synthesis)  Phosphocholine (intermediate product for producing phosphatidylcholine) |
|  |  | **MILD/ASYMPTOMATIC:** Upregulated serum proteins, such as S100-A8, S100-A9, serum amyloid A-1 protein (SAA1) and A-2 protein (SAA2), and alpha-1-antichymotrypsin (SERPINA3) |  |
| **Other Infections** |  | Patients with COVID-19 have a less severe dysbiosis of gut microbiome compared with patients with community-acquired pneumonia |  |

**C. Hub genes and pathway of innate immune response**

|  | **Transcriptomics** | **Proteomics** | **Genomics** |
| --- | --- | --- | --- |
| **COVID-19** | **SEVERE**:  IFN deficiency in the blood as a hallmark of severe COVID-19.  Genes encoding both TNFSF14 and OSM are downregulated in the PBMCs from COVID-19 patients with severe disease.  The gene encoding EN-RAGE, was over expressed in blood myeloid cells in patients with severe COVID-19.  NETs are significantly upregulated in severe disease patients.  Neutrophil associated with severe rather than mild disease:   - PD-L1 on neutrophils only found in severe COVID-19; - CD177 on mature activated neutrophils and of genes associated with anti-inflammatory functions (CD274 and ZC3H12A), including increased NET formation, coagulation and immune-thrombosis.   Monocytes with low expression of HLA-DR, and expression of marker genes of anti-inflammatory functions (e.g., CD163 and PLAC8) in severe COVID-19.  Robust levels of chemokines, including CCL2, CCL8, and CCL11, marked by a significant increase in circulating IL-6, IL1RA levels and significant elevation of CXCL9 and CXCL16 (chemo-attractants of T or natural killer (NK) cells, respectively), CCL8 and CCL2 (which recruit monocytes and/or macrophages), | **SEVERE:**  Top pathways associated with plasma ACE2:  clathrin-mediated endocytosis signaling, actin cytoskeleton signaling, mechanisms of viral exit from host cells, EIF2 (eukaryotic initiation factor 2) signalling, and the protein ubiquitination pathway.  Significant downregulation of the tricarboxylic acid cycle (TCA) and glycolytic pathways in both mild and severe patients;  The hypoxia-inducible factors (HIF-1) signaling pathways and host defence pathways elevated.  Clinical data show significant increases of IL-6, IL-8, and IL-10 levels in severe patients. | **SEVERE:**  Several host's genes (OAS1-3, IRF7, IRF9, STAT1 and IFIH1) are highly expressed.  *HLA-B*46:01*: The most significant gene loci associated with disease severity were located in *TMEM189-UBE2V1* involved in the IL-1 signalling pathway.  Blood groups A and O are associated with decreased risk of COVID-19 than non-A and non-O groups, respectively. A GWAS found 3p21.31 gene cluster in patients with COVID-19  In locus 3p21.31, a cluster of six genes (*SLC6A20, LZTFL1, CCR9, FYCO1, CXCR6,* and *XCR1*), which have functions linked to COVID-19 pathogenesis, was significantly associated with susceptibility to COVID-19  - T allele (946T)–carrying people have higher IFN levels, along with a lower risk of SARS-CoV-2 infection, and they should be protected from infection. |
|  | **MILD/ASYMPTOMATIC**:  SARS-CoV-2 infection seems to induce an early, transient type IFN production in the lungs that induces ISGs in the peripheral blood.  Activated HLA-RhiCD11chiCD14+ monocytes were increased in patients with mild COVID-19, similar to patients with SARS-CoV- 2 negative flu-like illness.  In COVID-19 patients there is also an increase of XIAP associated factor 1 (XAF1)-, tumour necrosis factor (TNF)-, and FAS induced T cell apoptosis and COVID-19 activates distinct pathway (STAT1/IRF3) versus influenza (STAT3/NFkB). | **MILD/ASYMPTOMATIC**:  SARS-CoV-2 immunity in mild patients is characterized by a robust T-cell response, reflected in T-cell signalling activation and T-cell differentiation on admission, followed by subsequent rapid reduction |  |
| **Other Infections** | In SARS-CoV and MERS-CoV higher expression of ACE2 in the lungs. - Other respiratory viruses show a higher antiviral transcriptional response, associated with high type I IFN level and low chemokine expression. |  | In West Nile encephalitis, Tick-borne encephalitis, Dengue fever, Chikungunya, and SARS several host's genes (*OAS1-3, IRF7, IRF9, STAT1* and *IFIH1*) are highly expressed |

**D. Comorbidities COVID-19 associated not sharing COVID-19 pathogenesis**

|  | **Transcriptomics** | **Genomics** |
| --- | --- | --- |
| **COVID-19** | Several genes related to histone modifications (HAT1, HDAC2, KDM5B) were identified in severe COVID-19 patients with comorbidities.  Several genes related to histone modifications, such as HAT1, HDAC2, KDM5B, and positively correlated genes, as ADAM10, regulate ACE2 cleavage in human airway epithelia.  Several genes positively associated with ACE2 are regulated by KDM5B, and by specific histone acetylation (H3K27ac) and histone methylation (H3K4me1 and H3K4me3).  In the human lung, peaks for H3K4me1 and H3K4me3, as well as H3K27ac, are in the ACE2 locus, suggesting that ACE2 may be epigenetically regulated in the lung.  The transcriptomic differences identified between patients with hypertrophic cardiomyopathy (HCM) and controls, on cardiac tissue flash frozen showed that the single most upregulated gene in HCM was the transcript for ACE2, suggesting the heart’s compensatory effort to mount an anti-hypertrophic and anti-fibrotic response.  Impaired ileal ACE2 expression leads to worse outcomes in colon disease indicating that ACE2 pathway has a protective, tryptophan-dependent anti-inflammatory mechanism in severe IBD.  A total RNA sequencing colon samples from 9 fatal COVID-19 cases, even in absence of any gastrointestinal symptoms, found that there is a dramatic change in the transcriptome in the colon tissues in the fatal cases compared to healthy controls.  In ACE2-positive, proximal tubular epithelial cells obtained from urine samples from COVID-19 patients suggested an ACE2-coregulated proximal tubular epithelial cell (PTEC) expression that could interact with the SARS-CoV-2 infection processes. | In the African/African-American population, several ACE2 polymorphisms are significantly associated with cardiovascular and pulmonary conditions by altering the angiotensinogen (AGT)-ACE2 interactions, such as *p.Arg514-Gly.*  Since *ACE2* gene is located in the X chromosome, initially older men with comorbidities seems to have severe COVID-19 compared to women, suggesting that ACE2 expression might be sex biased in the lung.  *HAT* and *HDAC* modulate chromatin and DNA condensation by changing histone acetylation status, which could facilitate ACE2 expression. |

**E. Comorbidities associated and related to COVID-19 pathway**

|  | **Metabolomics/ Lipidomics** |
| --- | --- |
| **COVID-19** | **SEVERE:**  Specific factors in COVID-19 individuals with diabetes:  the presence of bone marrow changes (myeloidosis) that predispose to an excessive proinflammatory response and contribute to insulin resistance, reducing vascular repair and worsening function of the heart, kidney, and systemic vasculature;  increased circulating furin levels that could cleave the spike protein and increase SARS-CoV-2 infectivity;  dysregulated autophagy that may promote replication and/or reduce viral clearance;  gut dysbiosis that leads to widespread systemic inflammation;  Metabolites able to discriminate COVID-19 patients from healthy subjects are:  lipids quantified in the positive mode (phosphatidylcholine, phosphatidylcholine, and phosphatidylethanolamine);  lipids quantified in the negative mode (arachidonic acid, oleic acid, glycerophosphoethanolamines, and glycerophosphoethanolamines);  KEGG functional enrichment analyses shows the activation of two significant pathways: fat digestion and adsorption and glycerophospholipid metabolism.  Critical patients are characterised by triglycerides upregulation; dysregulation of porphyrin metabolism; activation of gluconeogenesis; abundance of oleic acid and arachidonic acid; glycerophosphocholine and sphingomyelins downregulation, increased 21-hydroxypregnenolone level (essential for corticosterone synthesis); activation of kynurenine pathway; downregulation of choline and its derivatives; accumulation of mannose and its derivatives.  L1TG and PL in HDL1 were overexpressed in severe patients, compared with mild patients, while cholesterol in HDL1, HDL2, HDL4, and its components were downexpressed. The levels of some key proteins of lipoprotein metabolism in mild and severe COVID-19-infected patients were significantly lower than in healthy controls |
|  | **MILD/ASYMPTOMATIC:**  Non-critical patients are characterized by strong alteration of lipids, including acylcarnitines, diacylglycerols, fatty acids, glycerophosphoethanolamines, glycerophosphocholines, sphingomyelins, and triacylglycerols; perturbation of phenylalanine,  Metabolic alteration in mild/asymptomatic COVID-19 patients include: altered tryptophan metabolism into the kynurenine pathway, which regulates inflammation and immunity;  Metabolite levels in these pathways correlated with clinical laboratory markers of inflammation (i.e., IL-6 and C-reactive protein) and renal function (i.e., blood urea nitrogen). |
| **Other Infections** | Lipid rafts are associated with SARS-CoV-2 virulence and may correlate with different severity profiles also in bacterial (E. coli), mycobacteria, and viral infections (HIV and coronaviruses) |

## **Table S6. Pathways analysis based on Reactome Pathways Relation (Additional file 3).**

## **Table S7. Detailed table of evidences (Additional file 4).**

## **12. Annexes**

### **Annex 1 – Report from Working Group 1 - Molecular characterisation of the virus**

Working group 1: Isabella Abbate, Francesca Colavita, Giulia Matusali, Gabriella Rozera, Martina Rueca.

#### Viral genomic

Genome evolution and geographical distribution

The evolutionary history of SARS-CoV-2 was reconstructed starting from phylogenetic comparison with the related five subgenera of Betacoronaviruses, showing that SARS-CoV-2 receptor-binding domain in Spike (S) protein was closely related to SARS-CoV S [PMID: 32007145, TE01]. The pan-genome analysis of the Betacoronavirus genus in terms of origin, diversity and evolution showed that SARS-CoV-2 genome is quite distinct from those of other Sarbecovirus, with a close relationship with Bat-CoV and Pangolin-CoV [PMID: 32742815, TE02].

In GISAID database, SARS-CoV-2 genomes are classified by clades and lineages, providing a geographic distribution and genomic specificity. At the beginning of the pandemic, the ancestral type was soon replaced by the A2a type (clade G), characterized by the D614G non-synonymous mutation in Spike protein coding gene [PMID: 32474553, TE04]. The clades were named after their respective amino acid mutations: S (ORF8, L84S), V (ORF3a, G251V), I (ORF1ab, V378I), D (ORF1ab, G392D), and G (Spike, D614G) [PMID: 32793182, TE03]; the clades were named after their respective amino acid mutations: S (ORF8, L84S), V (ORF3a, G251V), I (ORF1ab, V378I), D (ORF1ab, G392D), and G (Spike, D614G) [PMID: 32793182, TE03]. At that time, Clade G was widely distributed globally, representing 71.14% of all the sequenced viral sequences, followed by S (10.85%), V (7.66%), D (1.03%), and I (1.70%) [PMID: 32841689, TE05]. Last up-dated GISAID database consulted by the cited references: 175,000 genomes (November 10th 2020).

Genomic hotspots for mutation, possible drivers of evolution and correlation with COVID-19 disease pathogenesis

More than half of recurrent mutations were found in ORF1ab, although a high mutation rate was observed in nsp3, nsp12 and nsp2. Ten hyper-variable genomic hotspots with high frequencies of mutated allele were also identified. Mutation P323L in RNA-directed RNA polymerase (RdRp or nsp12) was associated with higher mutation frequency in all genome, which could be related to the over activation of the RNA polymerase complex [PMID: 32470119, TE14]. All the SARS-CoV-2 genomic loci encoding nsps, except nsp11, were found to have the ratio of non-synonymous to synonymous substitutions (dN/dS) values <1. In structural genes, the same was true for S and M, whereas for *ORF3a*, *E*, *ORF6*, *ORF7a, ORF8*, *N*, and *ORF10* dN/dS was >1 [PMID: 33161087, TE11]. The analysis of 5,597 within S gene signals of positive selection were detected in four segments: the receptor binding domain (RBD); the segment encoding the S1 subunit; the furin cleavage site and the segment encoding the S2 and S2’ subunits [PMID: 33170902; PMID: 33088633, TE09, TE12]. By comparative genomics on Sarbecoviruses, it was observed strong protein-coding signatures for all named genes, for hypothetical ORFs 3a, 6, 7a, 7b, 8, and ORF3a. By contrast, ORF10 shows no protein-coding signatures but shows unusually high nucleotide-level constraint, indicating important but non-coding functions. ORF8 had a specific replication-enhancing effect in SARS-CoV. In contrast, deletions in ORF7b and ORF8 in SARS-CoV-2 showed similar replicative features of wild type virus. Moreover, people infected with deleted variants had a lower odd of developing hypoxia. Integrated comparative genomics and machine learning techniques identified in human coronavirus 11 regions, reliably predictive of high case fatality rate, occurring in nsp3, nsp4, nsp14, S, membrane glycoprotein (M), and nucleocapsid (N) [PMID: 33605421, PMID: 33184173, PMID: 33581339, PMID: 33170902, PMID: 32522874, PMID: 33161087, PMID: 33088633, PMID: 32723797, PMID: 32470119, PMID: 33024961, PMID: 30310104, PMID: 32694143, PMID: 32822564, TE06 – TE18].

Intra-host genomic variability

Clinical samples from SARS-CoV-2 infected individuals were analysed in order to identify small- and large-scale intra-host variations in the viral genome [PMID: 33086379, PMID: 32818852, TE19 - TE20]. It was observed spatial temporal redistribution of SARS-CoV-2 variants in respiratory and gastro-intestinal tracts in human hosts [PMID: 33086379, TE19], with a higher significantly genetic diversity observed in gastrointestinal compared to respiratory tract samples [PMID: 32818852, PMID: 32129843, TE20 - TE21]. A metatranscriptome sequencing on bronchoalveolar lavage fluid (BALF) samples showed intra host variants. The overall dN/dS ratio was significantly smaller than 1, suggesting a purifying selection [PMID: 32858978, TE22]. In clinical samples intra-host single nucleotide variants (iSNVs) were observed mainly in ORF1ab, S, ORF3a, ORF6, ORF7 and N coding genes.

#### Viral proteomics

Single viral protein studies

A novel signature was named nidovirus RdRp-associated nucleotidyltransferase (NiRAN), displaying a nucleotidylation activity and presenting upstream the N-terminal of the RNA-dependent RNA polymerase (RdRp) of all Nidoviruses. This was discovered and found to be able to influence SARS-CoV replication [TE24 PMID: 26304538]. Two mutations in the amino acid positions 3691 and 9659 (in *nsp6* and *ORF 10* regions respectively), confer lower stability to the protein structures and are possibly involved in autophagy [TE25, PMID: 32283146].

Cai et al 2005, combined protein modelling and molecular dynamics simulations, in order to identify the elements of the viral proteome that may explain the difference between SARS-CoV and SARS-CoV-2 pathogenicity in humans [TE26, PMID: 16212442]. Non-conservative substitutions in functional regions of the S, nsp1, and nsp3 might contribute to virulence differences. The highly conserved nsp5 protease was part of the mechanism of circumventing interferon (INF) antiviral response [TE27, PMID: 32941612].

Whole viral proteome

Diaz et al, re-analysing *in silico* all the SARS-CoV-2 human protein interactions of the published interactome of Gordon et al, found a modular hierarchical scale free structure among the total of 273 virus/host PP interactions [TE28 - TE29, PMID: 32754056, PMID: 32587094]. In particular, 6 viral nodes influenced the activity of 166 host nodes (59.7% of the total host nodes) in different cellular processes. In particular, virus proteins ORF8, M, and nsp7 were the three nodes with the most connections. Based on structural, dynamic and evolutionary SARS-CoV-2 protein properties a Viral Integrated Structural Dynamic Database (VIStEDD) was designed, named SARS-CoV-2 dynamicome, which showed highly conserved amino acid surface for both nsp6 and N proteins, likely to drive protein-protein interactions. Potential functional missense variants within ACE2/SLC6A19/transmembrane serine protease 2 (TMPRSS2) were also identified in S protein [PMID: 32587094, TE29].

#### Virus - host interactome

Viral RNA and protein interactions

Two works *in silico* identified SARS-CoV-2 RNA genomic regions more suited for interacting with proteins [PMID: 33068416 PMID: 32780783, TE33, TE34]. The first study indicated that the highest amounts of RNA structures were located at the 5’ end and in S and M. CatRAPID omics predicted that the 5’ end of SARS-CoV-2 had strong propensity to bind human proteins involved in viral infections, among which the ATP-dependent RNA helicase DDX1 and the ds-RNA specific editase ADAR and ADARB1. Other predicted interactions were with XRCC5 and XRCC6, the 2–5A-dependent ribonuclease RNASEL and 2–5-oligoadenylate synthase 2 OAS2 [PMID: 33068416, TE33]. The second study analysed the interactions between 5’ and 3’ untranslated SARS-CoV-2 regions, host RNA binding proteins and host microRNA. It elaborates a list of host proteins able to bind to 5' and 3' ends of SARS-CoV-2 genome, and a list of hsa micro RNA able to bind 3' end of SARS-CoV-2 genome, by using GISAID, RBPmap and microRNAs databases [PMID: 32780783, TE34].

Moreover, a potential silencer RNA (siRNA) targets conserved among all the studied SARS-CoV-2 genomes, was identified [TE32, PMID: 32577654]. Experimental studies involving deep sequencing of cell associated RNAs, discovered 3 SARS-CoV small viral (sV)RNAs (18-22 nt in length) in mouse SARS-CoV-2 lung infected cells, derived from nsp3 and one from N coding region. Specific antagomirs (anti-svRNA-N LNA), administrated prior the infection of mice with SARS-CoV, were able to reduce the pro-inflammatory response and therefore lung pathology of the mice in response to SARS-CoV infection [TE33, PMID: 33068416]. Two experimental researches provided a detailed description of the SARS-CoV-2 RNA–protein interactome in infected human cells using a similar experimental approach. [PMID: 32780783, PMID: 32297156, TE34, TE35]. The isolated fragments were analysed for spectrometry mass MS to identify the single proteins covalently associated to a particular RNA fragment characterized by sequencing the bound nucleic acid probe. Schimdt et al identified 15 SARS-CoV-2 proteins in SARS-CoV-2 Huh-7 cells (human liver cells), bound to SARS-CoV-2 genome and sub-genomic RNAs and 57 significantly enriched human proteins (core interacting). An expanded interactome included 104 proteins. Cellular nuclei acid-binding protein (CNBP) and La-related protein (LARP), two of the most enriched viral RNA binders, were able to restrict SARS-CoV-2 replication in infected cells. In human cell lines it was shown that SARS-CoV-2 replication is reduced when PPIA, ATP1A1 and ARP2/3 complex is pharmacologically inhibited [PMID: 33349665, TE37]. The SARS-CoV-2 RNA–protein interactome was applied to SARS-CoV-2 Vero E6 (kidney monkey) and to Huh-7 cells: 163 (VeroE6) and 229 (Huh7.5) host factors were found to bind the SARS-CoV-2 RNA. The authors also provided a comparison of their results to those of Schmidt et al highlighting an overlaid of 30/48 (63%) interactions, whereas only 11/332 host factors (3.3%) were found in PPIs network obtained by Gordon et al 2020 [PMID: 33349665, PMID: 33052334 PMID: 32142651, TE37, TE38, TE51]. GO enrichment analysis of the binder proteins to the SARS-CoV-2 genome identified all the host pathways involved in SARS-CoV-2 infection. Functional interrogation of these proteins revealed most of them protected the host from virus-induced cell death and regulated SARS-CoV-2 pathogenicity [PMID: 33052334, TE38]. Hoffmann et al 2021 designed a focused high-coverage CRISPR-Cas9 library, targeting 332 members of the Gordon identified SARS-CoV-2-host protein interactome, to determine which of the interacting host proteins were essential for infection by SARS-CoV-2 of Huh-7.5 hepatoma cells compared with the infection of other three seasonal human coronaviruses (HCoV-229E, HCoV-NL63, and HCoV-OC43). No sgRNAs targeting genes likely to dominate the screen (e.g., the SARS-CoV-2 cellular receptor, ACE2) were used for improving signal-to-noise ratios. Virus-specific differences in Rab GTPase requirements and glycosylphosphatidylinositol (GPI) anchor biosynthesis were found, among different coronaviruses, whereas multiple pan-coronavirus factors were identified involving cholesterol homeostasis [PMID: 33357464, TE45].

To identify host genes regulating SARS-CoV-2 infection in Vero E6 SARS-CoV-2 infected cells, *C. sabaeus* genome-wide pooled CRISPR screening assay was carried out, using a library of 83,963 targeting single guide RNAs (sgRNAs). The strongest resistance hit was for the viral receptor ACE2. CTSL (Cathepsin L protease) was also positively selected. The authors did not observed enrichment of TMPRSS2, nor the proteases TMPRSS4 and FURIN, which have also been implicated in SARS-CoV-2 entry [PMID: 33147444, TE39].

Virus-host protein-protein interactions (PPI)

Davies et al 2020, identified both unique and shared nsp2 and nsp4 host cell protein binding partners of three human betacoronavirus strains (OC43, SARS-CoV, SARS-CoV-2) [PMID: 33263384, TE40]. Nadeau et al 2020 implemented the interactome of SARS-CoV-2 and human proteins published by Gordon et al 2020 and found 329 Gene Ontology terms for which the SARS-CoV-2-interacting human proteins were significantly clustered in PPI networks. A new protein motif discovery algorithm, LESMoN-Pro, allowed to identify 9 amino acid motifs for which the associated proteins were clustered in PPI networks [PMID: 33103435, TE41]. Perrin-Cocon et al 2020 obtained a large coronavirus-host interactome built upon 1311 PPIs retrieved from literature. Small protein complexes that appeared particularly relevant to coronavirus infection were: EIF4E2-GIGYF2 dimer involved in the repression of protein translation and the MAT2A-MAT2B complex controlling SAM synthesis; DNA-PK kinase contributing to INF induction; mitochondrial proteins PHB, PHB2 and STOML2 regulating mitophagy [PMID: 32811513, TE42]. Messina et al 2020, found that, although the amino acid sequences of the S-glycoprotein were different between the different Human CoV, the best 3D structural overlap shared by SARS-CoV and SARS-CoV-2 was in the region involved with ACE2 binding receptor. The host interactome, linked to the S-glycoprotein of SARS-CoV and MERS-CoV, mainly highlighted innate immunity pathway components, such as Toll Like receptors, cytokines and chemokines [PMID: 32522207, TE43]. A dataset of physical and molecular interactions focused on proteins from SARS-CoV-2, SARS-CoV and other members of the Coronaviridae family has been manually extracted and curated by the International Molecular Exchange (IMEx) Consortium and can be accessed in the standard formats recommended by the Proteomics Standards Initiative (HUPO-PSI) at the IntAct database website (https://www.ebi.ac.uk/intact) [PMID: 33206959, TE44].

Multilayer (transcriptomics, proteomics) virus-host interactions

Other studies aimed to integrate, in a multilayer analysis, viral-host transcriptomics and proteomics and in few cases; they try to predict different SARS-CoV-2 disease phenotypes. Interrogation of chromatin accessibility and gene expression studies conducted on diseased human lungs identified age-increased activity and immune regulation of cis-regulatory elements (cCREs) that could control the expression of SARS-CoV-2 host entry gene TMPRSS2. Another distal cCRE was found to be linked to SLC6A20, a gene expressed in alveolar cells, with functional association with the SARS-CoV-2 receptor ACE2 [TE52, PMID33164753]. Other studies, in order to characterize the in vivo crosstalk of SARS-CoV-2 with the human host, re-analysed available scRNAseq data set obtained from BALF of mild and severe COVID-19 patients to find SARS-CoV-2 transcripts among the cellular RNA. SARS-CoV-2 transcripts were present only in samples derived from individuals with severe disease, while were strongly enriched in the ciliated and epithelial progenitor population and in the SPP1+ macrophage population, all expressing CD147, a potential new SARS-CoV-2 receptor. Differential gene expression was found between infected cells and bystander macrophages, while co-infection with human metapneumovirus (hMPV) was detected in a severe patient associated with a strong downregulation of the IFN response pathway [TE53, PMID 32479746]. Moreover, a new multi-omics network approach allows to generate human-SARS-CoV-2 interactome based on Calu-3 cell line (CSI), using transcriptome data from SARS-CoV-2 infected cells collected in GEO database, pertaining to lung epithelial cells with human interactome [TE48, PMID32895641]. A Master Regulator Analysis was predicted, on the basis of multiple available datasets obtained by coronavirus *in vitro* infected human 2B4 bronchial epithelial cells and the largest human lung RNAseq dataset, available from the Genotype - Tissue Expression (GTEx) Project consortium, showing which parts of the human interactome were most affected by the SARS-CoV-2 infection. The results indicated that apoptotic and mitochondrial mechanisms, as well as ACE2 protein receptor downregulation, were the principal host components affected by SARS-CoV-2 infection [TE49, PMID: 32244779]. Finally, The COVID-19 Disease Map was obtained to build a knowledge repository of molecular interactions between SARS-CoV-2 and host cells, following skills of domain experts [TE50, PMID: 32371892].

#### Viral entry

The S protein consists of the S1 region, containing the receptor-binding domain (RBD), and the S2 region harbouring the fusion peptide. Based on evidences from SARS-CoV, the angiotensin-converting enzyme 2 (ACE2) and the cellular serine protease TMPRSS2 have been identified as host cell entry mediators for SARS-CoV-2 [TE51, PMID: 32142651]. Two studies identified the protein Neuropilin-1 (NRP1) as an additional cellular factor, which may facilitate viral entry and infectivity [TE52, TE53, PMID: 33082294, PMID: 33082293]. Alternative SARS-CoV-2 entry factors have been investigated, including heparan-sulfates (HS), Sialic Acid, L-SIGN/DC-SIGN CD147, glucoseregulated protein 78 (GRP78), angiotensin II receptor type 2 (AGTR2), and receptor for advanced glycation end products (RAGE) [TE54, PMID: 33164751].

Entry factors expression in human tissues

Several studies investigated the expression of ACE2 and TMPRSS2 in human tissues by bulk and scRNAseq, Western blot, mass spectrometry and immunohistochemistry (IHC). Analysing ACE2 RNA expression in multiple organ systems (by scRNAseq and transcriptomic datasets reported in HPA, GTEx, FANTOM5 databases), allowed to find ACE2 protein in 44 different tissues, with the highest expression in intestinal tract, kidney, testis, gallbladder, and heart. Other tissues showed a low (e.g., thyroid gland and adipose tissue), very low (e.g. liver, female reproductive organs, epididymis, seminal vesicle, pancreas, and placenta) or no (brain, lymphoid tissues, skin, smooth muscle, and immune cells) expression [TE55, PMID: 32715618].

Moreover, cornea and conjunctiva have a high expression of ACE2 [TE55, PMID: 32715618]. A novel cloud-based platform named nferX® showed [TE56, PMID: 32463365]. results consistent with previous research and underlined the expression of ACE2 and other coronaviruses receptors in more mature subset of small intestine enterocytes (supporting the potential oro-fecal transmission) and ACE2 expression in tongue keratinocytes and nasal epithelial cells (supporting dysgeusia and anosmia observed in COVID-19 patients).

Contrasting data were published for ACE2 and TMPRSS2 in the public databases for the placenta and the ovary [TE57, TE58, PMID: 32669955; PMID: 32662421].

Importantly, a very low or lack of ACE2expression was reported in organs considered as the main target for SARS-CoV-2 replication including the lung, bronchus, and nasal mucosa [PMID: 32715618, PMID: 32463365, PMID: 32675206, TE55, TE56, TE59]. Nevertheless, TMPRSS2, CD147 and GRP78 proteins were expressed in airway epithelial cell lines and in lung tissues, indicating that alternative receptors for SARS-CoV-2 may facilitate host cell infection and suggesting a dynamic regulation of ACE2 expression in human lung upon SARS-CoV-2 infection [TE59, PMID: 32675206]. A meta-analysis of human, non-human primate, and mouse scRNAseq datasets for putative SARS-CoV-2 targets, revealed that Type II pneumocytes (ATII), nasal goblet secretory cells, and ileal absorptive enterocytes are ACE2+/TMPRSS2+ [TE60, PMID: 32413319].

Genetic variants of entry factors may influence the expression level of cellular factors involved in SARS-CoV-2 entry. Through GTEx Portal, SNPnexus, and Ensembl four variants associated with differential expression of *TMPRSS2* have been identified (rs469390, rs2070788, rs383510, and rs464397). The AA rs469390, GG rs2070788, TT rs383510, TT rs464397 genotypes are associated with the higher expression in the lung and lower frequencies in Asian compared to European, and American populations [TE61, PMID:32703421]. On other hand, eight genetic variants of ACE2 (from gnomAD https://gnomad.broadinstitute.org) were found as mapping to region binding between RBD domain in S glycoprotein, not showing any modification in the affinity with the virion [TE62, PMID: 32410735].

A more comprehensive analysis based on scRNAseq profiling of the RNA levels of 28 genes for SARS-CoV-2 and coronavirus-associated receptors and factors (named “SCARFs”), in both somatic and reproductive tissues, suggests that intestine, kidneys, placenta, and spermatogonia are most permissive for coronaviruses, while the nasal epithelium shows high expression of both promoting and restricting components [TE63, PMID: 32946807]. SCARFs comprise receptors and co-receptors (i.e., ACE2, CD147, DPP4, ANPEP, CD209, CLEC4G/M), proteases as priming factors (i.e., TMPRSS2, TMPRSS4, TMPRSS11A/B, Furin, Cathepsin B), proteins involved in viral trafficking and replication interacting with SARS-CoV-2 (i.e., TOP3B, MADP1, Rho-GTPases, AP2 complex). Restriction factors against entry were also included in the analysis (i.e., LY6E, IFITM1-3).

SARS-CoV-2 interaction with entry factors

The role of the viral proteins involved in the entry of SARS-CoV-2 and their interactions with cellular factors is addressed by several studies. The relevance of the following structural aspects have been evidenced. At first, an aromatic domain proximal to the transmembrane region of the S2 subunit [TE64, PMID: 15654751]. The heavy glycosylation status and flexibility of the S protein [TE65, PMID: 32817270], and the presence of residues Q493 and P499 in the RBD for a stable binding to ACE2 [TE62, PMID: 32410735]. The stronger association of the SARS-CoV-2-RBD to the ACE2 peptidase domain (ACE2-PD) compared to SARS-CoV (2-fold higher binding affinity) is a property that may be associated to the greater severity of the new virus [TE66, TE67, TE68, PMID: 32075877, PMID: 32155444, PMID: 32790406]. One site change at position 23403 in the Wuhan reference strain (NC_045512.2, Wh-01) leading to Aspartate to Glycine mutation in S residue 614 (D614G), was identified in early march 2020 and soon spread and became prevalent worldwide. D614G seems not to be associated with disease severity, but instead with higher viral loads. Spike’s D614G lays in a pocket adjacent to the fusion peptide near the expected TMPRSS2 cleavage site, suggesting that there could be differences in the propensity and/or requirement for TMPRSS2 of the D614G variant. This hypothesis was not confirmed *in vitro* in ACE2 expressing 293T cell [TE70, PMID:32697968]. An additional cleavage site for neutrophil elastase (ELANE) near the S1-S2 junction of the Spike protein was recently found in D614G subtypes using *in silico* model. This site facilitates host cell entry for D614G when ELANE level at the site of infection is elevated, such as in individuals exhibiting genotype-dependent deficiency of α1-antitrypsin (AAT), the ELANE inhibitor. The AAT deficiency is highly prevalent in European and North-American populations, but much less so in East Asia [TE69, PMID: 33556558]. Most importantly, D614G-bearing virions were intrinsically lower resistant to neutralization by convalescent sera, an issue of particular interest nowadays with the spread of the new variants of concern [TE70, PMID:32697968]. In fact, B.1.351, P1 lineages, and B.1.1.7 strains, have, amongst others, K417N, E484K, and N501Y “escape mutations”. These substitutions seem to alter the interaction of viral S protein with receptor hACE2, with a greater transmissibility compared to the previous circulating strains, and especially for B.1.351 and P1 lineages, lead to a reduction in neutralization activity, potentially compromising vaccine and immune-therapies (i.e. monoclonal antibodies) efficacy [TE71, PMID: 33581803].

In addition, viral evolution studies are showing how the RBD of the Spike protein is highly variable and immune escape mutations (i.e., N439K) emerge independently in multiple lineages spreading worldwide, leading to the accumulation of different changes, which may increase the risk of a significant failure of host immunity or therapy against these variants [TE72, PMID:33621484]. Dissecting the mechanism of SARS-CoV-2 entry and the interactions with host factors is of paramount importance for the understanding of pathogenesis, discovery of disease biomarkers, and development of drugs. Two studies explored the cross talk and integrative network between SARS-CoV-2 and host proteins during the entry and later step of viral replication. In the first, 38 human proteins were identified showing physical affinity to the structural surface viral proteins S, E, and M. Four of these proteins, namely ATP6V1A, AP3B1, STOM, and ZDHHC5, represent the seed proteins, localized at the cellular membrane, which may enable SARS-CoV-2 binding. An interactome was generated identifying 45 neighbouring proteins directly connected to the four seed proteins and their expression in the lung was confirmed. When analysing the expression of the 49 factors identified, eight genes were differentially expressed in primary human lung epithelium upon SARS-CoV-2 infection. The enrichment analysis showed over representing pathways in the hub of receptor binding machinery including association with immune mechanism, trafficking of vesicles and small molecules, and cell cycle. Moreover, the study showed that miRNAs can regulate the proteins of interconnecting hubs, and therefore can be used as therapeutics for SARS-CoV-2. Indeed, seven miRNA (miR-124-3p, let-7g-5p, miR-133a-3p, miR-133b, miR-218-5p, miR-22-3p, and miR-506-3p) targeting a minimum of three interconnected proteins and involved in the intermediate phase between receptor binding and viral replication were identified [TE73, PMID: 32695025].

The Calu-3-specific human-SARS-CoV-2 interactome (CSI) generated using a multi-omics network-biology-fuelled approach, showed that SARS-CoV-2 proteins interact with central nodes of the interactome implicated in core molecular and cellular pathways, including eIF2 signaling/translation, striated muscle contraction, protein ubiquitination pathway, viral infection, apoptosis T cell receptors and p38 MK2 pathway. Through network centrality analyses, 33 high values SARS-CoV-2 targets were identified as possibly involved in viral life cycle and disease progression. A probabilistic modelling using iDREM (interactive Dynamic Regulatory Events Miner) revealed 63 significant regulators expressed in SARS-CoV-2 infected Calu-3 cells, 14 of these regulators were also identified analysing the transcriptome of PBMC and BALF cells derived from COVID-19 patients. Three groups of significant interactomes were proposed: the first involved in creating a protective environment for the virus upon infection (e.g. modulating autophagy, apoptosis, trafficking) and includes the ATP6V1A; the second in viral entry and further replication steps and includes the prohibitin (PHB) proposed as alternative receptor or co-receptor; iii) the third in sustaining viral proliferation and pathogenesis and includes proteins involved in oxidative stress and inflammation processes. Stomatin, a protein involved in platelet aggregation, is another factor identified [TE48, PMID: 32895641].

### **Annex 2 – Report from Working Group 2 – Pathways**

Working Group 2: Manuela Antonioli, Fabiola Ciccosanti, Gian Maria Fimia.

Signal Transduction

Like other viruses, both SARS-CoV-1 and SARS-CoV-2 infection strongly modulate signal transduction within infected cells [TE67, PMID: 20392858]. Among viral proteins, nsp8, nsp13, N and ORF9b play a major role in host signalling regulation [Gordon et al., 2020, PMID: 32353859]. Interestingly, the analysed literature highlights that mTOR signalling (a key cellular regulator of gene expression) is highly modulated by SARS-CoV-2 leading to PI3K/AKT activation and TNF cascade immediately after viral entry [Gordon et al., 2020, TE74, TE75, TE76, TE77, TE78, PMID: 32353859, PMID: 20392858, PMID: 32691695, PMID: 32971089, PMID: 33060197, PMID: 33259812]. In SARS-CoV-2 infected HuH7 cells, Appelberg and colleagues analysed several downstream effectors of these pathways, revealing a dose-dependent activation of Akt, mTOR, S6K1 and 4E-BP1 at 24 hours post infection (hpi) [TE75, PMID: 32691695]. Of note, the mTOR-regulated translational repressor LARP1 is a host mRNA binding protein, which interacts with the SARS-CoV-2 nucleocapsid [Gordon et al., 2020, PMID: 32353859]. Another important pathway is represented by TNF signalling (Receptor-interacting serine/threonine-protein kinase 1 (RIPK1) interacting with SARS-CoV-2 nsp12) [TE75, PMID: 32691695]. Moreover, *in silico* analysis of RNAseq and protein-protein interactions data highlight *TNF* signalling as hub significantly upregulated in the lung epithelial in response to SARS-CoV-2 [TE76, PMID: 32971089]. Less literature is reported about MAPK family cascade signalling, mainly related to SARS-CoV-1 [PMID: 32522207, PMID: 33206959, PMID: 20392858, TE43, TE44, TE74].

Translation and post-translation modifications

All mRNAs processes rely on host cap-dependent translation to produce viral proteins. Translation is targeted also during SARS-CoV-2 infection; on one hand, the virus usurps cellular translational machinery to promote its replication, on the other cells tempt to reduce translation to contrast the infection. SARS-CoV-2 N, nsp2 and nsp8 proteins interfere with the eIF4F-cap complex (*e.g.* eIF4A inhibitors or more general translational inhibitors) [Gordon et al., 2020, PMID: 32353859]. Except for five viral proteins (*i.e.* S, N, nsp8, ORF7a and ORF9b), only minor changes of the global translation are reported following SARS-CoV-2 infection in Caco-2 cell lines, revealing an extensive increase in the translation machinery of the host, with significant enrichment of splicing and nucleobase synthesis [TE79, PMID: 32408336]. Studies on Vero E6 cells and iAT2s revealed that most of host proteins decreased in abundance (24 hpi) consistently with a host mRNA translation inhibition [TE80, PMID: 32645325]. The early activation of mTOR signalling, could also contribute to the strong upregulation of host protein abundance, reflected by the protein levels modulation of 40S (e.g., RPS6/11/26) and 60S (e.g., RPL6/12/19) ribosomal subunits and by the upregulation of the mitochondrial 28S ribosome at 3–6 hpi, necessary for viral replication [TE78, PMID: 33259812]. Moreover, SARS-CoV-2 infection strongly modulates post-translational modifications, such as phosphorylation and ubiquitination [TE80, PMID: 32645325]. Hekman RM and colleagues reported the increase of positive phosphorylation on AKT (*i.e.* S129), and of its substrate YBX1 (S102), known to attenuate its repressor function. In addition, an increase of eIF2S1 S49/S52 phosphorylation, linked to reduced translation of 5′-capped mRNA, suggests a cellular response to prolonged SARS-CoV-2 infection (24 hpi) [TE78, PMID: 33259812]. An extensive analysis of the phosphoproteome following SARS-CoV-2 infection delineates the modulation of different kinases axis, leading to the modulation of several cellular pathways (*e.g.* EPHA2 – focal adhesion, RPS6Ks – cell survival, CDKs – cell cycle progression, AKT– cell growth, survival and motility, p38, JNK, ERK – stress responses, ATM, and CHEK1/2 – DNA) [TE81 33845483]. Viruses’ hijack ubiquitination is an emerging differential ability to modulate host proteins ubiquitination. This mechanism could represent a keystone in understanding diversely impact of SARS-CoV-2 compared to other coronaviruses [TE41, PMID: 33103435]; MERS-CoV specifically interacts with the E3-ubiquitin ligase UBR5 through nsp14 [TE77, PMID: 33060197]; SARS-CoV-1 interacts with E3-ligase CTLH (nsp4) [Gordon et al., 2020, PMID: 32353859]. TE40, PMID: 33263384], a target of the NEDD8-Cullin E3 ligase pathway (MRFAP1) and with the proteasome subunit p42 (binding to SARS-CoV-1 N) [TE41, PMID: 33103435, TE82, PMID: 20478047]. Some E3-ubiquitin ligases were also described to overlap in both SARS-CoV-1 and SARS-CoV-2 interactome analysis; e.g. viral nsp14 binds RNF170, an E3-ligases involved in the degradation of inositol 1,4,5-triphosphate receptors (IP_3_Rs) [TE41(33103435]. Only four host proteins (*i.e.* EFNB1, POLR2B, TYMS and DHFR) have shown concomitant ubiquitination and decrease at the protein level. By contrast, a plethora of E3-ubiquitin ligases are emerging in the viral-host protein-protein interactions both from *in silico* [PMID: 32522207, TE43], TE41(33103435, TE49, PMID: 32244779) ], and *in vitro* studies, specifically related to SARS-CoV-2 [Gordon et al., 2020, PMID: 32353859] TE40, PMID: 33263384) , TE81, PMID: 33845483, TE83, PMID: 32838362]. Several ubiquitination sites have been identified on SARS-CoV-2 proteins which could be achieved by E3-ligases observed in the SARS-CoV-2 interactome (*e.g.* ORF3 and TRIM47, WWP1/2, STUB1; M and TRIM7; NSP13 and RING1) as cellular response to counteract viral replication [TE80, PMID: 32645325]. In addition, other viral proteins linked to host ubiquitination are represented by interactions ORF7a-UBE2J1 (E2 ligase), ORF3a-TRIM59 (E3 ligase), and ORF6-RNF20, RNF40, RFWD3, and MYCBP2 (E3 ligase), suggesting that the host ubiquitin system could be targeted by these viral protein complexes; TRIM59 and MIB1 were found associated to SARS-CoV-2 ORF3a and nsp9, respectively [Gordon et al., 2020, PMID: 32353859], TE83, PMID: 32838362]. E3-ligase activity of Cullins (CUL) s requires neddylation, thus NEDD8-activating enzyme (NAE) could represent a druggable target to delineate the role of CUL s in SARS-CoV-2 infection [Nakagawa et al., 2016, PMID: 27712623].

Immune system

Omics data derived from both *in silico* and *in vitro* studies and highlighted the immune system as one of the most relevant cellular process modulated by SARS-CoV-2 [TE73, PMID: 32695025, TE76, PMID: 32971089]. The analysis of SARS-CoV-2 interactome with the host revealed that the IFN pathway is targeted by nsp13 through interactions with TBK1 and TBKBP1, by nsp15 which binds cellular RNF41 and Nrdp1 and, by ORF9b interacting with TOMM70 [Gordon et al., 2020, PMID: 32353859]. In addition, the interaction between ORF6 and an INF-inducible mRNA nuclear export complex may antagonize host IFN signalling in both SARS-CoV-1 and SARS-CoV-2 [Frieman et al., 2007, PMID: 17596301, [Gordon et al., 2020, PMID: 32353859]. Overall, PPIs reported by Gordon, highlight potential cellular targets of SARS-CoV-2, which could orchestrate the weak production of type I and III IFN, contributing to the severe forms of COVID-19 [Gordon et al., 2020, PMID: 32353859]. Interestingly, another SARs-CoV-2 PPIs study revealed RAE1 and TBK1 as cellular proteins associated to SARS-CoV-2 regulation of type I IFN and NF-κB pathways upon viral infection [TE86, PMID: 32619390]. Another important cellular mechanism involved in immunity, upregulated during SARS-CoV-2 infection and associated to severe COVID-19 is represented by NF-κB [Hirano et al., 2020, PMID: 32325025]. Several cellular proteins involved in NF-κB pathway were associated with SARS-CoV-2 proteins, *i.e.* TLE1, 3, and 5 (binding nsp13), and NLRX1, F2RL1 and NDFIP, interacting with viral ORF9c. TRIM59 and MIB1, two E3 ubiquitin ligases regulating antiviral innate immune signalling, bind SARS-CoV-2 ORF3a and nsp9, respectively [Gordon et al., 2020, PMID: 32353859]. SARS-CoV-2 affected innate immunity at different levels. Recent proteomic studies report a decrease of proteins involved in innate immunity such as the cytokine SPP1, the growth factor GRN, and the receptor tyrosine kinase AXL [TE85, PMID: 32591346], and upregulated pathways involved in innate immunity as TNF, NOD-like receptor (NLR) and RIG-I signalling [TE76, PMID: 32971089]. Complement cascade is also reported as innate immune mechanism system modulated by SARS-CoV-2 infection [TE76, PMID: 32971089, TE85, PMID: 32591346]. Finally, a modulation of cytokine signalling pathways was reported using omics approaches both *in vitro* and *in silico* [PMID: 32522207, PMID: 33206959, PMID: 32971089, TE76 TE43, TE44].

Cell cycle

A SARS-CoV-2-time course infection analysis revealed a rapid reshapes of several host mechanisms leading to cell cycle arrest. In particular, DNA damage response is rapidly activated 6 hours post infection (hpi), characterized by the upregulation of the hypoactive cell cycle kinase, ATR. Moreover, SARS-CoV-2 upregulates proteins involved in the mitotic/cell cycle, translation, and DNA damage responses (*i.e.* pH2AX, PCNA, pS6 and γTub) [TE78, PMID: 33259812]. A proteomic study on A459 lung cells expressing ACE2 suggests that phosphorylation and ubiquitination sites are modulated by the involvement of central regulators of cell cycle progression, such as CDKs kinases [TE81, PMID: 33845483]. Altogether, omics data following SARS-CoV-2 infection suggest a modulation of cell cycle in the host cells in response to infection. It is important to consider that the majority of the reported studies have been performed in cell culture, which represents an undisputed model for infection; however, cell cycle is intrinsically altered in the culture system introducing a bias in COVID-19 disease model conceptualization.

Other pathways

The network analysis of cellular pathways highlighted by omics studies both *in vitro* [TE44, TE74, TE77, TE78, TE80, PMID: 33206959, PMID: 20392858, PMID: 33060197, PMID: 33259812, PMID: 32645325] and *in silico* [TE73, PMID: 32695025]*,* showing that cell death and, specifically apoptosis, is altered [TE78, PMID: 33259812]. In addition, SARS-CoV-2 nsp4, nsp5, nsp8 and ORF9c interactions with host proteins revealed an important association with mitochondria [Gordon et al., 2020, PMID: 32353859]. SARS-CoV and SARS-CoV-2 ORF9b interacts with the mitochondrial outer membrane protein Tom70, the major import receptor which mediates the translocation into mitochondria of cytosolic pre-proteins [TE77, PMID: 33060197, Young et al., 2003 PMID: 12526792] Moreover, autophagy seems to be altered by SARS-CoV-2 infection; in particular, the downregulation of ATP6AP1 hampers SARS-CoV-2 infection both in A549 and Caco2 cell lines models, supporting a pro-viral role of this protein, which facilitates the assembly of the vacuolar ATPase and autophagy [TE40, PMID: 33263384]. Stukalov and colleagues observed an accumulation of several autophagic proteins (*e.g.* SQSTM1, GABARAPL2, NBR1, CALCOCO2, MAP1LC3B, TAX1BP1) following ORF3 expression, also observed in virus-infected cells (SQSTM1, MAP1LC3B), suggesting an inhibition of the autophagic flux [TE81, PMID: 33845483]. Both *in silico* and *in vitro* studies describe a general impact of SARS-VoV2 infection on metabolism [TE41 PMID: 33103435, TE44 PMID: 33206959, TE73 PMID: 32695025, TE78 PMID: 33259812, TE83 PMID: 32838362]. Importantly, the host protein SigmaR1 involved in lipid remodelling and ER stress response binds the SARS-CoV-2 nsp6 and ORF9c [Gordon et al., 2020 PMID: 32353859, TE77 PMID: 33060197, TE83 PMID: 32838362] sharpening, among others, the metabolism of lipids. Another important cellular mechanism is represented by the vesicle-mediated transport [Gordon et al., 2020, PMID: 32353859], [TE45, TE44, TE73, TE86, PMID: 33357464, PMID: 33206959, PMID: 32695025, PMID: 32619390]. Approximately 40% of SARS-CoV-2-host interactions are linked to vesicle trafficking, engaged nsp2, nsp6, nsp7, nsp10, nsp13, nsp15, ORF3a, E, M and ORF8 [Gordon et al., 2020, PMID: 32353859]. It is emerging that different coronaviruses hijack specific Rab GTPases during the infectious cycle. Host RAB2A and RAB7A are critical for HCoV-229E, HCoV-OC43 and HCoV-NL63 infection, while RAB10 and RAB14 play a major in SARS-CoV-2 infection [TE45 PMID: 33357464].

### **Annex 3 – Report from Working Group 3 - Host signatures**

Veronica Bordoni, Alessandra Sacchi, Chiara Farroni, Saeid Najafi Fard, Delia Goletti, Chiara Agrati.

#### Soluble mediators during SARS-CoV-2 infection (proteomic and metabolomic data)

Soluble factors including cytokines, chemokines, growth factors, inhibitory factors, hormones, and metabolites can influence induction, activation and functions of innate immune cells both in the peripheral circulation and in tissues. Soluble mediators in innate immune cells play a fundamental role as both sender and receiver. The kinetics and concentrations of soluble mediators modulate cellular responses during SARS-CoV-2 infection. Therefore, we can summarize the modulation of soluble factors during SARS-CoV-2 infection based on classical cytokines and chemokines and Growth factors, inhibitory molecules, hormones and metabolites.

Proinflammatory mediators

During the early immune response, the activation type I/III IFN responses represent key local innate immune players and are associated to chemokines (CCL2, CCL8 CXCL2, CXCL8, CXCL9, and CXCL16) [TE87 PMID: 32416070] and proinflammatory mediators (IL20, CCL27, IL21, PLG, IL6, IL17, CCL2, CXCL10, IL1b) [TE88 PMID: 32788344]. The next phase of SARS-CoV-2 infection is characterized by a huge release of inflammatory mediators, called “cytokine storm”. The systemic profile of these soluble markers was evaluated in plasma/serum of patients with different disease severity: mild disease is characterized by increase of IFNa, IFNg, IL6, IL-10, IL1RA [TE87,TE88,TE89, PMID: 32416070, PMID: 32788344, PMID: 32661059]; severe disease is characterized by a downregulation of IFNa and a parallele up-regulation of IFNb, IFNg ,IL6, TNF, IL10, CCL7 [TE89 PMID: 32661059, TE90 PMID 33171100, TE94, PMID 32492406]. Finally, critical disease is characterized by the upregulation of IL6, TNFs, IL-10, RIPK-3 [TE89 PMID: 32661059]. Several proteins differentially expressed with the severity of COVID-19 are linked to IL-6-mediated proinflammatory cytokine signaling: (1) the CD14-LBP LPS recognition system, (2) upregulation of LRG1, an angiogenesis and anti-apoptotic factor associated with inflammation, and (3) upregulation of the LGALS3BP, can induce the expression of IL-6 by stromal cells in Galectin-3-dependent manner. For this reason, Galectin-3 is an interesting druggable target to mitigate TGF-b-mediated fibrosis and aberrant inflammation [TE92, PMID 32619549]. Low levels of plasma GSN are usually associated with inflammation, because the protein is recruited to the sites of tissue injury to handle the released actin. In particular, abundance of plasma GSN (pGSN) correlates strongly with COVID-19 severity, while cytosol GSN (cGSN) levels are not reduced, suggesting that the reduction of pGSN in COVID-19 may result from decreased biosynthesis and/or increased clearance from plasma, rather than tissue injury [TE92, PMID 32619549]. Another important class of mediators is the acute phase proteins (APPs), which are involved in early states of immune responses to viral infections. In addition to CRP, the serum amyloids A-1 (SAA1), A-2 (SAA2), and A-4 (SAA4), the serum amyloid P-component (SAP/APCS), and alpha-1-antichymotrypsin (SERPINA3), Alpha-1-acid glycoprotein 1 (ORM1), Alpha-1-acid glycoprotein 2 (ORM2), Alpha-2-Glycoprotein 1 (AZGP1), were found to be elevated in severe COVID-19 [TE92 PMID 32619549, TE93 PMID 33128875, TE94 PMID 32492406, TE95 PMID 33096026]. Upregulation of APPs can in turn induce inflammatory cytokines and neutrophil activation, as shown for S100A8 and S100A9, thus possibly contributing to amplify the cytokine storm in COVID-19 patients. APPs may also participate in platelet aggregation and activation of coagulation cascades, and CRP can play a role in the activation of the complement system. Moreover, APPs can influence lipid metabolism in the course of a pro-inflammatory response. For example, SAA2 can displace APOA1 within HDL particles altering HDL particles’ functions and possibly contributing to increased clearance of HDL observed in COVID-19 patients. Multiple correlations between ORM1 and increased blood triglycerides, insulin resistance and cardiovascular disease were also reported. Moreover, AZGP1 may act as an adipokine involved in lipid metabolism. During COVID-19, proteins involved in fatty acid metabolism as FETUB and CETP, known as inflammation suppressors, were reduced in serum, as was PI6, a suppressor of chemotaxis [TE93 PMID 33128875]. Moreover, a machine learning-based classifier of eleven plasma proteins, including orosomucoid 1/alpha-1-acid glycoprotein-1 (ORM1), orosomucoid- 1/alpha-1-acid glycoprotein-2 (ORM2), fetuin-B (FETUB), and cholesteryl ester transfer protein (CETP), was generated to determine and predict disease outcome [TE93 PMID 33128875]. A consistent and sustained upregulation of apoptosis-associated proteins CASP8, TNFSF14, HGF, and TGFB1, with HGF discriminating between ICU and non-ICU cohorts, was reported [TE94 PMID 32492406]. In addition, the ICU group tends to be separated from non-ICU patients by OSM and S100A12, whereas all COVID-19 patients can be distinguished from healthy controls by CASP8, IFNγ, IL-18R and CCL8. Several enzymes with antimicrobial activity were increased in COVID-19 patient sera, including cystatin C (CST3), defensin A1 (DEFA1), and lysozyme C (LYZC), indicating a possible secondary bacterial infection [TE97 PMID 32786691].

Complement activation - Coagulation and Platelet Degranulation DA RIVEDERE LE PARTI GIALLE IN COMUNE CON FIMIA.

Another main class of APPs upregulated in severe COVID-19 patients are proteins of classical complement pathway (C1R, C1S, C5, C6 and C8A) as well as the alternative pathway factor B (CFB) and the complement modulators factors I (CFI) and H (CFH) [TE94 PMID 32492406, TE95 PMID 33096026, TE97 PMID 32786691, TE100]. Two regulators of complement system, Properdin (CFP) and Carboxypeptidase N catalytic chain (CPN1), are upregulated [TE92 PMID 32619549, TE94 PMID 32492406]. In severe COVID-19 patients, mannose and its derivatives resulted to be increased [TE94 PMID 32492406]. In the complement system, binding of mannose to lectin leads to cleavage of C2 and C4. Increases in pro-coagulant components (including Factors 5, 7, 10, kininogen 1, fibrinogen alpha and beta, SERPINF2 (alpha-1 antiplasmin) and CPB2 (thrombin-activatable fibrinolysis inhibitor, TAFI) is accompanied by increases in anticoagulant components, such as SERPINA1, SERPINA3, vitamin K dependent protein S (PROS1). In addition, Shu et al highlight the contribution of platelet degranulation and complement and coagulation cascades to the pathogenesis [TE93 PMID 33128875]. Several papers reported that the signature of pbmc/blood related to blood coagulation are closely related with the severity of COVID-19 [TE95 PMID 33096026, TE96 PMID 33140861, TE98 33597532, TE99 PMID 32747830]. McClain reported an increased expression of many thrombotic pathway genes in a subset of early COVID-19 cases including prekallikrein (KLKB1), Factor 12 (F12), the plasminogen activator inhibitor (SERPINE1) and others, along with decreased expression of antithrombotic protein S (PROS). Poor prognosis appeared associated with increased levels of D-dimer and fibrinogen degradation products, and with a decrease in F13A1 expression, suggesting that blood-clotting status may be one of the key factors to monitor in COVID-19 progression [TE99 PMID 32747830]. Similarly, the upregulation of cellular proteins related to blood coagulation was found in severe COVID-19 PBMCs. These findings imply that the dysfunction of platelet degranulation and coagulation cascades are closely related with the severity of COVID-19. Differently Xin Hou reported that, complement cascade, and coagulation cascades were down-regulated in early COVID-19 infection (C1R, C7) respect to influenza infection [TE88 PMID 32788344]. Most proteins involved in platelet degranulation were downregulated in SARS-CoV-2 infected patients [TE94 PMID 32492406, TE95 PMID 33096026]. including, GPLD1, CLEC3B, histidine-rich glycoprotein (HRG), heparin, thrombospondin, and plasminogen, which may be associated with observed thrombocytopenia in COVID-19 patient. This decrease could due to: i) platelet consumption by platelet aggregation in the lungs, ii) inhibition of platelet synthesis in the bone marrow, iii) platelet destruction by the immune system.

The abundances of other platelet-associated proteins were increased in COVID-19 samples versus non-COVID-19 samples, including serglycin (SRGN) and the von Willebrand Factor (VWF) and Fibronectin (FN1), which mediate the interaction of platelets with endothelial surfaces of injured vessels. Monitoring of unique peptides discriminating cellular (cFN) and plasma (pFN) isoforms of FN1 revealed that only cFN was significantly increased in COVID-19 patients [TE88 PMID 32788344].

Lipid Metabolism

Most of lipid-related molecules are decreased in moderate and severe COVID-19 patients, indicating a clear preference toward downregulation of lipids. Main observed alterations are related to:

- Lipoproteins. High density lipoproteins (HDL) are downregulated in sera of severe patients compared with healthy and non-severe COVID-19 patients. Very low-density lipoproteins (VLDL) component are upregulated [TE88, TE92, TE94, TE100, PMID: 33043283, PMID: 32619549. PMID: 32788344, PMID: 32492406]. A normal Apo-A1 to Apo-A2 ratio is maintained, indicating that SARS-CoV-2 infection did not modify HDL composition. These alterations are consistent with a remodeling of the lipoprotein particle phenotype in COVID-19 patients, with a reduction of the mean HDL size, an enlargement of the mean size of LDL, and increased level of VLDL subclasses with intermediate size [TE96, TE100, TE101] (PMID: 33140861, PMID: 33043283, PMID: 33207699).

- Serum triglyceride (TG) content and the mean concentration of TG-VLDL, TG-IDL, TG-LDL, and TG-HDL were significantly increased in COVID-19 patients. Inversely, the total cholesterol (TC) and mean concentration of bound TC-LDL and TC-HDL are decreased in COVID-19 serum, while TC-VLDL and TC-IDL levels are slightly increased [TE94, TE96, TE100, TE102, PMID: 32492406, PMID: 32610096, PMID: 33140861, PMID: 33043283]. The identification of high levels of TG suggests an increase in adipose tissue lipolysis in COVID-19 patients (PMID: 33207699, PMID: 32559180) [TE101, TE146]. The high levels of free fatty acids also confirm the role of adipose tissue in the circulating lipid profile [PMID: 33207699, PMID: 32559180, TE101, TE146].

- - Plasma glycerophospholipids including phosphatidic acids (PA), phosphatidylinositols (PI), and phosphatidylcholines (PC), major membrane components of circulating lipoproteins, are decreased. This decrease is accompanied with an increase in their corresponding lysophospholipids (i.e., LPA, LPI, and LPC), which indicates enhanced phospholipase A2 (PLA2) activity in COVID-19 patients [TE94, TE102, PMID: 32492406, PMID:32610096]. Importantly, PLA2 is required for coronavirus replication, contributing to form specialized membrane compartments for viral replication. Relevant for COVID-19, LPCs are known as important homeostatic mediators involved in all stages of vascular inflammation. Moreover, LPCs are demonstrated to potentiate the activation of T lymphocytes, macrophages and neutrophils [Kabarowski 2009, PMID: 19383550].

- Plasmenyl-PCs, lipids acting as potent antioxidants, known as plasmalogens, were decreased in most cases [TE95, PMID: 33096026].

- Sphingolipid classes of Sphingomyelins (SMs) and gangliosides GM3, displayed progressive increases with increasing severity, possibly reflecting the augmented secretion of these lipids into the circulation. These observations suggest that plasma lipidomic changes in COVID-19 patients may be due to alterations of exosome contents [PMID: 33096026, PMID: 33207699, TE95, TE101].

Oxidative Pathways of Cellular Energy Production

Metabolites of the tricarboxylic acid cycle (TCA) and beta-oxidation were reduced in COVID-19 particularly in severe patients, while metabolic intermediates of the glycolysis and pentose phosphate pathways are increased [TE90, TE96, TE100, TE101, TE102, TE103, PMID: 33140861, PMID: 32610096, PMID: 33171100, PMID: 33043283, PMID: 33103907 PMID: 33207699]. This reduction may be the consequence of lung functions declines and blood oxygen decrease, but may also reflect a response to change in nutrition, especially in severe patients. Ketone bodies induced by fasting conditions are also elevated in the serum of COVID-19 patients even when compared to healthy patient’s cohort collected under uncontrolled fasting conditions [TE100, PMID: 33043283]. The accumulation of TG and TG-VLDL in COVID-19 patients may be due to an impaired oxidation of mitochondrial acetyl-CoA. The elevation of glucose observed in the serum of COVID-19, can also result from impaired TCA and compensative increase of gluconeogenesis [TE100] (PMID: 33043283). The dysregulated metabolism of glyoxylate and dicarboxylate detected in COVID-19 patients is also indicative of energy metabolic dysfunction [TE101] (PMID: 33207699).

Amino Acid Metabolism

- Amino acids and their derivatives, are significantly decreased in the sera of COVID-19 patients [TE101, TE102, TE103, TE104, PMID: 32610096 PMID: 33043283 PMID: 32806897 PMID: 33207699]. Specifically, gluconeogenic (e.g., alanine, glycine, serine, glutamine, and histidine) and sulfur-containing amino acids (e.g., cysteine, taurine) tended to decrease (PMID: 32559180) [TE146]. Moreover, metabolites involved in arginine metabolism including urea cycle metabolic intermediates and arginine derivatives were decreased in the sera of COVID-19 patients [TE103, TE104] (PMID: 32559180, PMID: 32806897). The observed changes in amino acid levels could be indicative of liver dysfunction in SARS-CoV-2 infected patients. In fact, increased plasmatic and urinary levels of taurine are markers of acute hepatic failure(PMID: 28469468), while reduced glutamine levels are associated to insulin resistance and an increased risk of Type-2 diabetes(PMID: 31377179). These alterations could be triggered by high interferon levels, which may alter urea cycle activity during viral infection (PMID: 31784108). In addition, creatine, creatinine, polyamines spermidine and acetyl-spermidine are increased, suggesting renal dysfunction (PMID: 32559180) [TE146]. The Fischer’s ratio (ratio of branched-chain AAs, leucine, valine, isoleucine, to aromatic AAs, phenylalanine, tyrosine) is decreased, due to an increase in AAA (PMID: 32559180) [TE146]. A decrease in the Fischer’s ratio is an indication of liver dysfunction, which fails to catabolize aromatic amino acids released from endogenous proteins, thus accumulating in the circulation. Reduced circulating tryptophan levels are observed in COVID-19 patients, associated to elevated kynurenine levels [TE94, TE104, TE146, PMID: 32806897 PMID: 32492406 PMID: 32559180]. This ratio is altered in multiple diseases such as inflammatory lung disease and kidney disease. Kynurenine/tryptophan is a general measure of indole 2,3-dioxygenase (IDO) activity, which is induced by interferon gamma in response to viral infection to play an immunoregulatory role by limiting inflammation [Gelpi et al., 2017, PMID: 28511640].

#### Immune cell response (bulk RNAseq on peripheral blood)

Early SARS-CoV-2 infection triggers a powerful transcriptomic response in peripheral blood that is heavily interferon (IFN)-driven. There was a strong increase in IFN-related transcripts during the peak of disease activity: the transcription factor (TF) activity in active disease were related to inflammation and IFN signaling (e.g., IRF1 and STAT3) and hypoxic signaling (HIF1A) [TE105, 33296687]. Comparing to SARS-CoV-1 or influenza A virus (IAV), SARS-CoV-2 infection fails to induce significant type I interferon responses in PBMCs and whole blood, which corroborates the low concentrations of type I interferon in the circulation of COVID-19 patients [TE106, 32670298; TE87, 32416070]. The lack of type III IFN was also observed [TE87, 32416070]. Some ISGs, such as OAS2 and IFIT3, are upregulated like in other viral infections, while other ISGs were not associated with classical ISG response, showing relatively over (LY6E, OASL, IFI27, IFI6) or downexpression (RSAD2, IFIT2, CCL2, LAMP3) in SARS-CoV-2 [TE107, 32599245; TE98, 33597532]. In early presentation of COVID-19 in mild-moderate stages, a significant dysregulation of IL-1, JAK/STAT, IL-6, and IL-10 signaling pathways was observed compared to influenza or seasonal coronaviruses [TE98, 33597532]. The interleukin genes IL1RN and IL-1β were significantly upregulated in SARS-CoV-2 infection by mirroring the elevated protein levels of IL-1Ra and IL-1β. IL1RN and SOCS3 encode cytokine-signaling antagonists, and their overexpression suggests a negative feedback loops effect were elicited [TE108, 32407669]. Additionally, most of the differentially expressed proteins in COVID-19 vs healthy controls were localized to the cell nucleus. In particular, the related molecules (RIG-I, IPS-I, and TNF receptor associated factors (TRAFs) of RIG-I-like receptor signal pathways are associated to virus infection, as well as the activation of RIG-I pathway [TE109, 33060566]. Studies with more in-depth stratification by disease severity showed significant differences between mild and severe COVID-19-patients and healthy controls in multi-omics data, suggesting that the identified molecular changes are likely to reflect the severity of COVID-19. Severe patients expressed elevated levels of chemokines and chemokine receptors and exhibited a marked increase in neutrophils. SARS-CoV-2 targets the type I IFN system at multiple steps, thus interfering with a well-orchestrated interplay between antiviral and pro-inflammatory innate and adaptive defense mechanisms. Type I IFN response was impaired in severe and critical COVID-19 patients: striking downregulation of IFN-stimulated genes (ISGs) (such as MX1, IFITM1, and IFIT2), IRF-1 and STAT3, the absence of circulating IFN-β in COVID-19 patients with all disease-severity grades and the low IFN-α production in severe COVID-19 patients were reported [TE89, 32661059]. The low IFN-α levels correlated with the reduction and functional impairment of plasmacytoid dendritic cells (pDCs), the main cell source of IFN-α. Thus, type I IFN deficiency could be a hallmark of severe COVID-19 [TE89, 32661059]. Moreover, the expression of exhaustion related genes, such as BATF, IRF4, and CD274 (PD-L1), significantly increased with disease severity [TE89, 32661059]. Accordingly, T cell surface molecules (CD4, CD8a, CD8b, CD2, CD5, and CD7), and TCR signalling kinases and adaptors (LCK, FYN, SKAP1, CARD11, CAMK4, and BTN3A1) were downregulated in severe patients [TE83, 32838362; TE89, 2661059].

Severely ill patients exhibited also a trend towards greater IL-8, IL-10, IL-6 and IL-12 [TE96, 33140861; TE98, 33597532; TE83, 32838362]. Moreover, comparing the proteome changes in PBMCs isolated from severe and mild COVID-19 patients, the expression levels of neutrophil surface receptors (CXCR1, Siglec5, and CD177) and neutrophil granule contents (DEFA1) were dramatically upregulated in severe COVID-19. Further analysis of the signature components revealed that p38 MAPK activation was dominant in fatal cases, while higher levels of IL-13 and IFN-g were present in survivors [TE96, 33140861].

#### Innate immune cell compartment (scRNAseq/CyTOF)

The initial SARS-CoV-2 infection triggers dynamic changes of different cell types in peripheral blood, especially in innate compartment. An elevated neutrophil/lymphocyte ratio has been identified as a hallmark of severe COVID-19 [TE89, 32661059]. The neutrophil transcriptomic signature analysis showed excessive neutrophil activation, associated with severe rather than mild disease. These markers are genes encoding molecules associated with neutrophil extracellular traps (NETs) (AZU1, DEFA3, MPO, PRTN3, CTSG, myeloperoxidase, lactoferrin, and histones) and result upregulated in severe COVID-19 patients [TE96, 33140861; TE112, 32810438]. Moreover, Low Density Neutrophils with immature phenotype, including pro- and pre-neutrophils expressing S100A12, S100A9, ARG1, OLFM4, and PD-L1 were up-regulated in severe disease and associated with myeloid derived suppressor cells [TE112, 32810438]. An increase of classical CD14+ monocytes, especially in convalescence stages, non-classical CD16+ monocytes, natural killer (NK) cells and several bone marrow-derived precursor cell types was observed in the peripheral blood of COVID-19 patients [TE105, 33296687; TE89, 32661059; TE113, 33131070; TE112, 32810438]. In particular, down-modulation of HLA-DR, and marker genes indicative of anti-inflammatory functions (e.g., CD163, PLAC8, S100A8/A9/A12) in monocytes were associated with disease severity [TE112, 32810438, TE90, 33171100]. Accordingly, the up-regulation of HLA-DR was observed in patients who recovered [TE90, 33171100]. Megakaryocytes (MK) proportions are linked to inflammatory parameters, e.g., serum CRP, IL-6, and IFN-a and increase during the disease course [TE105, 33296687]. In critical patients, it was observed a strong increase in PKM transcript amount, encoding a pyruvate kinase involved in ATP formation [TE105, 33296687]. Moreover, the markers of exhaustion (PD-1 and Tim-3) were increased on NK cells [TE89, 32661059].

#### Adaptive immune cell compartment (scRNAseq/CyTOF)

SARS-CoV-2 infection has a strong effect on the transcriptional profile of T and B cells. ScRNAseq confirmed previous data showing the drop in the percentage of circulating lymphocytes, including CD4+ T cells, and CD8+ T cells relative to increasing severity [TE112, 32810438; TE144, 28831119; TE90, 33171100; TE89, 32661059]. Expression of IRF1, TP53, and CASP3 is increased in T, B cell subsets in COVID-19 patients compared to controls. TNFSF10 (TRAIL) and its receptor TNFRSF10A expression increased in T cells of COVID-19 patients compared to healthy subjects, while TNFRSF1B was just relatively upregulated in COVID-19 patients. In B cell subset, TNFSF10 and FADD were notably increased in COVID-19 patients [TE111, 32783921]. The decrease of T cell differentiation and survival (BCL11B) genes was observed in severe compared to mild COVID-19 [TE83, 32838362]. These data suggest the increased gene expression related to T cell apoptosis in COVID-19 patients that may be contributed to circulating lymphocytes depletion. SARS-CoV- 2 immunity in mild patients was characterized by the induction of genes related to a robust T-cell response, reflected in T-cell signaling activation and T-cell differentiation, followed by subsequent rapid reduction. Differently, a negative T-cell signaling was continuously observed in severe patients along time. CD4 T cells maintained certain level of T-cell activation in severe-survivors’ group, but it did not in the severe-fatality group [TE96, 33140861]. These results were confirmed by proteomic analysis showing a dramatic decreasing of transcriptional levels in severe compared to mild COVID-19: the expression of TCR subunits (CD3e, CD3g, and CD247); major histocompatibility complex (MHC) class II molecules (HLA-DRA); CD38 in CD4 and CD8 T cells [TE83, 32838362; TE89, 32661059]. The transcriptional signatures of the different T cells profiles show that severe patients present an increase of naive clusters and a decrease in activated effector T cells relative to moderate disease. A proliferative exhausted CD8+ T cell subpopulation emerges in moderate COVID-19 characterized by the expression of LAG3, TIGIT, and CD279, and counterintuitively, exclusively expresses proliferation markers (MKI67 and TYMS). This cell population displays high cytotoxic signatures, not fully losing its naïve signature. ScRNAseq data on CD4 T cells allowed identifying naive-like cells, featured by elevated naive-related transcripts of TCF7 andCCR7, while CD197 surface protein was reduced in COVID-19 patients. An unusual CD4+ T cell subpopulation exhibiting elevated cytotoxic transcripts PRF1 and GNLY increased in patients with moderate/severe infections [TE90, 33171100].

Su and colleagues evaluated the functionality of T cells by single cell multiplex secreatome: polyfunctionality was similar for healthy and mild cases, upregulated in moderate cases, with increased frequency of cells secreting granzyme B and perforin, and reduced in severe cases [TE90, 33171100]. Accordingly, T cells from COVID-19 survivors expressed high levels of IFNG and GZMB, while T-cell dysfunction was observed in the severe group [TE96, 33140861]. IL17-A, IL17-F are increased in COVID-19 patient samples, [TE90, 33171100], and genes related to IL17 signaling were significantly enriched in the severe-fatal group [TE96, 33140861]. The proportion of plasma cells and cycling plasma cells were increased significantly in COVID-19 patients and increase in plasmablasts/plasma cells was observed in severe compared to mild patients [TE111, 32783921; TE105, 33296687; TE98 33597532]. Moreover, subjects with early symptomatic COVID-19 exhibited marked upregulation of B-cell activation (CD79A/B) and a broad diversity of immunoglobulin genes (IGHG1, IGHV2-5, IGHV3.30, IGLV3-19, IGLV3-25, and others) compared to other infections (Influenza, other coronaviruses, and bacterial pneumonia) [TE98 33597532]. A significant activation of naive B cells (downregulation of FCER2 and upregulation of SLAMF7) and expansion of antibody-secreting cells (ASCs) was observed in moderate and severe samples compared to healthy and mild samples [TE90, 33171100]. Moreover, B-cell-activation-related pathways enrichment (PRDM1, XBP1, and IRF4), increase in B cell clonality, and an increase of memory B cells and PBs, dominated by the IgA and IgG isotypes, were observed during COVID-19 course [TE111, 32783921; TE105, 33296687].

#### Immune response in tissues

Local mucosal immune response to SARS-CoV-2 infection has been characterized by proteomic and transcriptional changes in the lower and upper respiratory tract as well as in BALF. Proteomic analysis revealed the up-regulation of iNOS and IL-1b and IL-6 protein in lung tissue of COVID-19 patients [TE113, 33131070]. Further proteomics analysis identified several key proteins, such as the SARS-CoV-2 entry-associated protease cathepsins B and L and the inflammatory response modulator S100 A8, A9, A11, A12, and P that are more highly expressed and translated in the fatal cases compared to controls [TE110, 33082228]. The transcriptional profiling of lung tissue showed the over-expression of genes related to neutrophil activation, generation of neutrophil extracellular traps (myeloperoxidase, lactoferrin, and histones) (TE110, 33082228), confirming the role of NET formation in the immunopathology induced by SARS-CoV-2 infection. The transcriptional profiling of nasopharyngeal swabs demonstrated the induction of genes related to pathways with known immune modulatory functions, including inflammatory response, IFN-α response, IL-6/JAK/STAT3 signaling and complement cascade activation in COVID-19 patients. A down-modulation of members of the PPAR complex as EP300 RXRA RARA SUMO1 NR3C1 CCDC88A was observed in CD14+/CD16+ cells from severe patients by scRNAseq [TE99, 32747830]. The bulk sequencing and analysis of the BAL fluid cells from COVID-19 patients also showed the upregulated expression of IL-1B, several chemokines and chemokines receptors (CXCL17, CXCL8, CXCL1, CXCL2, CCL2, CCL7 and CXCR2), IL1RN and SOCS3, calgranulin genes that exert pleiotropic functions in inflammatory disorders (S100A8, S100A9, and S100A12). The down-modulation of genes related with cell activation and signaling (NCKAP1L, DOCK2, SPN, DOCK10) was also described) [TE108, 32407669]. The colon transcriptome of COVID-19 patients suggested that SARS-CoV-2 affected host responses even at a site with no obvious pathogenesis. Up-regulation of genes related to the response to the Transforming Growth Factor beta (TGF-β) and down-modulation of genes involved in immune cell activation were found in fatal cases compared to controls [TE110, 33082228].

### **Annex 4 – Report from Working Group 4 – Phenotype**

Working Group 4: Chiara Montaldo, Francesco Messina, Francesco Nicola Lauria.

Studies with more in-depth stratification by disease severity showed significant differences between mild and severe COVID-19-patients and healthy controls in multi-omics data, suggesting that the identified molecular changes are likely to reflect the severity of COVID-19.

Key Genes and Proteins in SARS-CoV-2- host Interactions and pathogenesis in the Lung

#### Transcriptomics

The SARS-CoV-2 Spike protein primer TMPRSS2 is over-expressed in ciliated cells and type I alveolar epithelial (AT1) and SARS-CoV-2 RNA is highly co-localized in cells expressing TMPRSS2 [PMID: 33180746 TE115].

**SEVERE COVID-19**

As reported before, ISGs genes are highly expressed in respiratory airways [PMID: 32599245 TE107]. The up-regulated genes in the neutrophil activation pathways revealed the involvement in generation of NETs. Other upregulated genes are implicated in the hyaline membrane formation and pulmonary fibrosis PMID: 33082228 [TE110]. ‘Ribosome’’ and ‘‘Chemokine signaling pathways are the top two enriched KEGG categories. Genes involved in morphogenesis and migration of immune cells are under-expressed [PMID: 32407669 TE108]. Genes upregulated in the COVID-19 patients’ BALF recapitulate clinical characteristics, such as increased cytokine signaling causing a “cytokine storm”, hypoxia (HIF1A, HLF), inflammasome and sepsis-related genes (IL1R1/2, IL5RA, IL33, IL31RA) [PMID: 32497778 TE116]. PPARꓬ (a member of peroxisome proliferator-activated receptor family), may play a key role in the cytokine storm of inflammatory monocytes/macrophages in the SARS-CoV-2-infected lung; in fact, a correlation was found between disease severity and reduced expression of several members of the PPARꓬ complex. [PMID: 33015591 TE 114] CD48 is associated with increased disease severity while CD40 is not. In the BALF at the single-cell level, the expression of the lipopolysaccharide (LPS) sensors TLR2 and TLR4 is induced: TLR2 expression increases with disease severity, especially in the CD14-CD16 cell populations, whereas TLR4 induction is less dramatic. HLA-B is highly expressed in all lymphoid and myeloid cell subpopulations in the lungs of the patient with severe COVID-19 [PMID:33015591 TE114]. The enrichment analysis of almost 2000 significant DEGs identified by an integrated gene expression meta-analysis of two datasets of ARDS and venous thromboembolism, suggested inflammation–coagulation–hypoxemia convolutions in COVID-19 pathogenesis, highlighting the TP53, KAT2B, DHX9, RELA, RBX1, PSMB2 as top hub genes [PMID: PMC7746517 TE117].

#### Proteomics

**SEVERE COVID-19**

The upregulated DEPs in the BALF of severe COVDI19 patients were: immunoglobulins, pro-inflammatory and extracellular factors of the ‘cytokine-mediated signaling network’, such as Tenascin-C (TNC), Mucin 1/KL-6 (MUC1), Lipocalin-2 (LCN2), periostin (POSTN), Chitinase 3-like 1 (CHI3L1 or YKL-40), Lactoferrin (LTF), and S100A12). Moreover, in this population, it was found a significant increase of suface antigens, such as lymphocyte antigen 6D/E48 antigen (LY6D), CD9 antigen, CD177 antigen, and prostate stem cell antigen (PSCA) [PMID: 33098359 TE118].

**MILD/ASYMPTOMATIC COVID-19**

In mild and severe COVID-19 patients, an increasing level of CCL2 was reported. CCL2 is an attractive chemokine for monocytes, memory T lymphocytes and basophils, and is associated with lung inflammatory disorders, such as acute respiratory distress syndrome (ARDS), asthma, and pulmonary fibrosis [PMID: 32788344 TE88].

#### Genomics

**SEVERE COVID-19**

A multi-gene cluster on chromosome 3 was significantly associated with respiratory failure. LZTFL1 and SLC6A20 are located within these loci: the first is strongly expressed in human lung cells, the second encodes for the sodium– imino acid (proline) transporter 1 (SIT1), functionally interacting with ACE2 [PMID: 33092637 TE130]. Pathways involved in COVID-19 pathogenesis in the lung are listed in Table S4 A.

DEG and DEP analysis in other organs and tissues

####

#### Genomics

**SEVERE COVID-19**

A test of genome-wide methylation profiling of circulating cell-free DNA in plasma showed that the concentration of cell-free DNA correlates with COVID-19 severity, being significantly increased in patients requiring intubation [PMID: 33521749 TE120].

#### Transcriptomics

Although there is not yet direct evidence that altered expression of intestinal ACE2 directly affects SARS-CoV-2 intestinal entry and tropisms to different intestinal sites, pro-inflammatory cytokines can upregulate ACE2 in colonic epithelial cells of IBD patients, compared to healthy subjects [PMID: 32915959 TE121]. Trophectoderm cells in peri-implantation embryos, as well as syncytiotrophoblast and extravillous trophoblast cells in the maternal–fetal interface, strongly co-expressed ACE2 and TMPRSS2, indicating SARS-CoV-2 infection susceptibility [Chen et al. 2020, PMID: 32837754]. However, it is unlikely that SARS-CoV-2 infection has long-term effects on male and female reproductive function [PMID: 32622411 TE122]. ACE2 exhibits the highest co-expression correlation with TMPRSS2, SLC6A20C, and LZTFL1 in the cornea compared to the lungs and retinal pigment epithelium (RPE) suggesting that the cornea may provide susceptibility for SARS-CoV-2 entry [PMID: 33049061, TE123].

**SEVERE COVID-19**

Mitochondria-localized NADPH oxidase 4 can be modulated by ACE2 upregulation. Such complex can produce reactive oxygen species (ROS) in the mitochondria. Oxidative stress caused by ROS excessiveness was indicated as a major player in COVID-19 pathogenesis and severity [PMID: 33049061, TE123]. Single-cell sequencing contributed to a better understand the etiology of the neurological sequelae. The cerebrospinal fluid (CSF) of neuro-COVID patients exhibited an expansion of de-differentiated monocytes and of exhausted CD4+T cells. Neuro-COVID CSF leukocytes featured an enriched INF signature but less pronounced than in viral encephalitis. Moreover, broad clonal T cell expansion and curtailed INF response was seen in severe compared with mild neuro-COVID patients [PMID: 33382973, TE124].

#### Proteomics

Viral entry of SARS-CoV-2 into the host cell is not facilitated by gut inflammation associated with chronic colitis [PMID: 32975064, TE125]. COVID-19 patients have less severe dysbiosis of gut mycobiome compared with patients with community-acquired pneumonia [PMID: 32598884, TE126]. Expression data from patients with normal endometrium showed that viral gene expression increases with age, suggesting that the endometrium of older women undergoing ART is at higher risk of viral infection [PMID: 32641214 TE127].

**SEVERE COVID-19**

Specific proteins, including lung proteins, peripheral blood cellular proteins (CARD9, CD177, CHI3L1, PODXL, S100A12 and TNC), plasmatic proteins (CP, CTNNA3, LY6D, and PSCA), TNC and KL-6 are significantly higher on serum and BALF of severe patients [PMID: 33098359 TE118]. Upregulated SAA1, SAA2, and alpha-1-antichymotrypsin are predictive factors of potential progression from moderate to severe forms [PMID: 33011738 TE128].

In severe patients, there is a significant suppression of lymphocyte and monocyte count [PMID: 32492406 TE94].

Many DEPs mediate diverse immune signaling pathways related to viral infection, inflammatory response, and immune cell activation and migration. DEPs between COVID-19 and influenza were identified. Among them, high amounts of IFNG, IL6, CXCL8, CXCL10 and CCL2 were reported in mild and severe COVID-19 patients [PMID: 32788344 TE88]. A multi-omics approach on peripheral blood and plasma samples showed down regulation of brain-enhanced proteins regulating neurotransmitter synthesis (GLS, OGDH, DLD, etc.), neurotransmitter transport (GLUL, GLUD2, GLUD1), neurotransmitter receptors (HTRA3, GRIK3, GRIA3), and proteins including ENO1, MBP, and NEFM [PMID: 33140861 TE96]. Significant proteomic changes in the liver of COVID-19 patients suggest that liver injury (such as accumulation of lipid and fibrosis) may be directly associated with SARS-CoV-2 infection [Leng et al. 2020, PMID: 33419962 ].

**MILD/ASYMPTOMATIC COVID-19**

Upregulated serum proteins, such as protein S100-A8, protein S100-A9, SAA1, SAA2, and alpha-1-antichymotrypsin were found in mild COVID-19 patients [PMID: 32492406 TE94].

#### Metabolomics, lipidomics

The homeostasis of glycolysis, lipogenesis, heme and ketone biosynthesis, gluconeogenesis, fatty acid oxidation, and cholesterol biosynthesis is imbalanced: LEPR-JAK1-STAT3 and ADIPOR-AMPK are activated, mediating β-oxidation activation, and leading to insulin resistance. Moreover, the insulin signalling activates both the mTORC1 (AKT2-P, mTORP, and EIF4E2) and mTORC2 (mTOR-P, PRKC, ACACA-P, and ACACBP) pathways leading to protein synthesis, cytoskeletal organization, and fatty acid biosynthesis. The fatty acid transporters (CD36, SLC27A1, and SLC27A4), fatty acid acetylase (CPT1), and lipid synthesis associated enzymes (AGPAT1 and AGPAT5) are highly expressed in the liver, possibly leading to the generation of lipid droplets through the activation [Leng et al. 2020, PMID: 33419962]. Metabolomic analyses on colostrum samples from COVID-19 puerperant women and healthy puerperant women, identified 340 metabolites revealing the alterations of aminoacyl-tRNA biosynthesis and aromatic amino acid metabolism. The levels of metabolites involved in tryptophan catabolism were significantly decreased in breastmilk of COVID-19 patients. Phenylalanine and its decarboxylation product phenethylamine, intertwine with more immune-mediated DEPs. The analysis identified also lipids with significant changes between the two groups of women [PMID: 33097684 TE129].

**SEVERE COVID-19**

In severe COVID-19 patients’ sera it was found increased level of CRP and AST, down-regulation of multiple apolipoproteins (APOA1, APOA2, APOH, APOL1, APOD, and APOM) associated with macrophage functions [PMID: 32492406 TE94], downregulation of liver-enhanced proteins regulating the transport of sterol and cholesterol and up-regulation of those involved in acute inflammatory response in both mild and severe patients [PMID: 33140861 TE96]. Pathways involved in COVID-19 pathogenesis in other organs and tissues are listed in Table S4 B.

Hub genes and pathways of innate immune response

#### Transcriptomics

SARS-CoV-2 viral load in nasopharyngeal swabs during the early stages of the disease was found to be higher in severe patients compared to mild ones [PMID: 32526012 TE119].

Antiviral transcriptional response, marked by low type I IFN levels and elevated chemokine expression, was found lower compared to other respiratory viral infections [PMID: 33092637 TE130]. As described above, the upregulation of genes involved in type I IFN signalling, mainly belonged to the NF-kB pathway, contrasts with a striking downregulation of ISGs in critical patients [PMID: 32661059 TE89].

During COVID-19 there is also an increase of XIAP associated factor 1 (XAF1), tumour necrosis factor (TNF), and FAS inducing T cell apoptosis. SARS-CoV-2 activates distinct pathway (STAT1/IRF3) compared with influenza (STAT3/NFkB); the differences in the expression of key factors, including IL6R and IL6ST, in the two infections support the clinical observations of increased proinflammatory cytokines in COVID-19 patients [PMID: 32783921 TE111].

**SEVERE COVID-19**

A recent transcriptomic analysis of nasopharyngeal tissues identified molecular signatures associated with COVID-19 severity, highlighting a dysregulation of immune related pathways, such as cytokine receptor signaling, complement and coagulation cascades, JAK-STAT, and TGF- b signaling in all severe patients. The excessive release of cytokines and chemokines such as CCL2, CCL22, CXCL9 and CXCL12, certain INFs and interleukins related genes like IFIH1, IFI44, IFIT1 and IL10 were significantly higher in sever patients compared to mild and moderate ones [PMID: 33425248 TE131]. Several genes related to histone modifications (HAT1, HDAC2, KDM5B) were identified in severe COVID-19 patients with comorbidities [PMID: 32526012 TE119 ]. The expression of genes encoding both TNFSF14 and OSM are downregulated in the PBMCs from sever patients suggesting a tissue origin for these cytokines. The gene encoding EN-RAGE, was expressed at high levels in blood myeloid cells in sever patients suggesting the pulmonary origin of the proinflammatory cytokines observed in plasma [PMID: 32788292 TE132].

As reported before, neutrophil transcriptomic analysis revealed excessive neutrophil activation linked to severe rather than mild disease. In fact, severe patients showed highly dysregulated myeloid cell responses marked by the appearance of neutrophil precursors due to emergency myelopoiesis, and dysfunctional neutrophils expressing PD-L1, and exhibiting an impaired oxidative burst response. Expression of PD-L1 on neutrophils only found in severe COVID-19, and increased in later stages of the disease, has been associated with T cell suppression, suggesting that neutrophils in severe COVID-19 might exert suppressive functions. An associated finding was the expression of CD177 on mature activated neutrophils and the identification of genes associated with anti-inflammatory functions (CD274 and ZC3H12A). Moreover, severe patients show transcriptional programs induced in immature neutrophils, including pro- and pre-neutrophils, align with other observations in severe patients, including increased NET formation, coagulation and immune thrombosis. Monocytes characterized by low HLA-DR expression and anti-inflammatory functions (e.g., CD163 and PLAC8) appeared in severe patients. The defective monocyte activation, along with dysregulated myelopoiesis, may induce continuous and harmful tissue inflammation, with ineffective host response [PMID: 32810438 TE112]. IL-17 signaling pathway genes were significantly upregulated in severe-fatal groups. The p38 MAPK activation was dominant in fatal cases, while higher levels of IL13 and IFNG were present in survivors. For the mRNA-based model, low CD3E levels and high OLAH levels, result highly concordant with the immune responses in COVID-19 patients [PMID: 33140861 TE96]. Immune cell landscape is reprogrammed with age and is characterized by T cell polarization from naive and memory cells to effector, cytotoxic, exhausted and regulatory cells. COVID-19 promoted age-induced immune cell polarization and gene expression related to inflammation and cellular senescence [PMID: 32780218 TE133].

**MILD/ASYMPTOMATIC COVID-19**

SARS-CoV-2 infection results in an early wave of IFN-a and strongly induces an ISG signature in moderate patients, and results in an early, transient type I IFN production in the lungs that induces ISGs in the peripheral blood, primarily in patients with mild or moderate disease [PMID: 32788292 TE132]. Activated HLA-DRhiCD11chiCD14+ monocytes were increased in mild COVID-19 patients, similar to patients with SARS-CoV-2 negative flu-like illness. In both COVID-19 and Influenza, plasma cells increase significantly [PMID: 32810438 TE112].

#### Genomics

A genetic study by high-depth whole genome sequencing, to evaluate the association between genome variants and COVID-19 severity, revealed that COVID-19 progression and severity could be determined by both the monogenic and complex genetic basis [PMID: 33298875 TE134].

**SEVERE COVID-19**

The most significant gene locus associated with severity were located in *TMEM189–UBE2V1* that involved in the IL-1 signaling pathway. The p.Val197Met missense variant that affects the stability of the TMPRSS2 and ACE2 binding, displays a decreasing allele frequency among the severe patients compared to the mild and the general population. *HLAA* 11:01, B*51:01,* and *C*14:02* alleles were significantly more prevalent in severe and critical patients compared to mild and moderate patients.

[PMID: 33298875 TE134].

#### Proteomics

SARS-CoV-2 induces robust levels of chemokines, including CCL2, CCL8, and CCL11. Enhancement of generalized inflammation, marked by a significant increase in circulating IL-6, IL1RA levels and significant elevation of CXCL9 and CXCL16 (chemo-attractants of T or natural killer (NK) cells, respectively), CCL8 and CCL2 (which recruit monocytes and/or macrophages), and CXCL8 (a classic neutrophil chemoattractant) suggest that the presence of these cells may be a primary driver of the signature pathology observed in COVID-19 patients [PMID: 32416070 TE87]. Many proteins associated with the immune response were downregulated in urine samples. Most of the upregulated proteins are involved in the complement and coagulation cascades, natural killer cell-mediated cytotoxicity, and platelet activation. Immunosuppression and tight junction impairment occur in the early stage of COVID-19 infection, while the immune response is activated at the late stage of the disease [PMID: 33203833 TE135].

The top canonical pathways associated with plasma ACE2 included clathrin-mediated endocytosis signaling, actin cytoskeleton signaling, mechanisms of viral exit from host cells, eukaryotic initiation factor 2(EIF2)-signaling, and protein ubiquitination pathways. A prominent relationship was shown between plasma ACE2 and actin cytoskeleton signaling, mechanisms of viral exit from host cells, and protein ubiquitination pathways, and a relationship of plasma ACE2 levels with EIF2- and mTOR signaling. [PMID: 32981365 TE136]. A comprehensive proteomics-based investigation of nasopharyngeal swabs showed that few of the host proteins such as interleukin-6, L-lactate dehydrogenase, C-reactive protein, ferritin, and aspartate aminotransferase were upregulated and the most important pathways identified by enrichment analysis were neutrophil degranulation, interleukin-12 signaling pathways, and mRNA translation [PMID: 33558857 TE137].

**SEVERE COVID-19**

Significant downregulation of TCA and glycolytic pathways was observed in both mild and severe patients compared with healthy controls; hypoxia-inducible factors (HIF-1) signaling pathways and host defence pathways were elevated in these patients. Clinical data show significant increases of IL-6, IL-8, and IL-10 levels in severe patients compared with mild patients [PMID: 33140861 TE96]. A recent proteomic study identified 91 plasma DEPs between mild and severe patients: the proteome signatures captured the host response to SARS-CoV-2 infection, highlighting the role of neutrophil activation, complement activation, and platelet function and indicated a high specificity of several inflammatory modulators, particularly IL-6, IL-1B, and TNF [PMID: 33376242 TE138].

**MILD/ASYMPTOMATIC COVID-19**

SARS-CoV-2 immunity in mild patients is characterized by a robust T-cell response, reflected in T-cell signaling activation and T-cell differentiation on admission, followed by subsequent rapid reduction [PMID: 33140861 TE96]. Pathways involved in innate immune response to SARS-CoV-2 infection are listed in Table S4 C.

Comorbidities

1. Comorbidities COVID-19 associated not sharing COVID-19 pathogenesis

#### Genomics

*ACE2* polymorphisms are likely to be associated with cardiovascular and pulmonary conditions by altering the angiotensinogen (AGT)-ACE2 interactions, (e.g.,p.Arg514-Gly in the African/African-American population). This gene is along the X chromosome. An initial finding showed that older men with comorbidities are more likely to have severe COVID-19 compared to women, indicate ACE2 expression might be sex biased in the lung. HAT and HDAC modulate chromatin and DNA condensation by changing histone acetylation status, were positively associated, thus modulating gene transcription of ACE2. This could occur in lung tissue, facilitating ACE2 expression, as observed during lung cancer and COPD [PMID: 33092637 TE130].

#### Transcriptomics

The expression of the gene encoding the ACE2 receptor in lung tissue is upregulated in diseases reported to be comorbidities associated with severe COVID-19. ACE2 is significantly upregulated in lung transcriptome studies suggesting that patients with COPD or PAH, and even people who smoke, may have higher chances of developing severe COVID-19. Several genes related to histone modifications, such as HAT1, HDAC2, KDM5B, and positively correlated genes, as ADAM10, regulate ACE2 cleavage in human airway epithelia. Several genes positively associated with ACE2 are regulated by KDM5B, and by specific histone acetylation (H3K27ac) and histone methylation (H3K4me1 and H3K4me3). In the human lung, peaks for H3K4me1 and H3K4me3, as well as H3K27ac, are in the ACE2 locus, suggesting that ACE2 may be epigenetically regulated in the lung. These findings suggest that the higher expression of ACE2 in the lungs is associated with higher chances of developing severe COVID-19, by facilitating SARS-CoV-2 entry into lung cells [PMID: 32526012 TE119].

The transcriptomic differences identified between patients with hypertrophic cardiomyopathy (HCM) and controls, on cardiac tissue flash frozen showed that the single most upregulated gene in HCM was the transcript for ACE2, suggesting the compensatory effort of the heart to mount an antihypertrophic response. However, given that SARS-CoV-2 uses ACE2 for viral entry, the increase in ACE2 transcript expression and protein levels in the heart may confer increased risk for COVID-19 severe outcomes [PMID: 32448590 TE139]. Impaired ileal ACE2 expression leads to worse outcomes in colon disease indicating that ACE2 pathway has a protective, tryptophan-dependent anti-inflammatory mechanism in severe IBD [PMID: 33160965 TE140]. A total RNA sequencing colon samples from nine fatal COVID-19 cases, even in absence of any gastrointestinal symptom, showed a dramatic change of transcriptome in the colon tissues, in fatal cases compared to healthy controls [PMID: 33082228 TE110]. Similar cellular programs were seen in ACE2-positive proximal tubular epithelial cells obtained from urine samples of COVID-19 patients, suggesting a consistent ACE2-coregulated proximal tubular epithelial cell (PTEC) expression program that may interact with the SARS-CoV-2 infection processes. ACE2 is co-expressed in PTECs with a set of genes that also function in establishing viral replication, host responses, and innate immunity [PMID: 33038424 TE141]. Pathways involved comorbidities COVID-19-associated not sharing COVID-19 pathogenesis are listed in Table S4 D.

1. Comorbidities associated and related to COVID-19 pathway

Lipidomics and Metabolomics

Specific factors may complicate COVID-19 in individuals with diabetes including: 1) the presence of bone marrow changes (myeloidosis), that predispose to an excessive proinflammatory response and contribute to insulin resistance, reducing vascular repair and worsening function of heart, kidney, and systemic vasculature; 2) increased circulating furin levels, that could cleave the spike protein and increase SARS-CoV-2 infectivity; 3) dysregulated autophagy, that may promote replication and/or reduce viral clearance; 4) gut dysbiosis, that leads to widespread systemic inflammation, increased glucose and sodium absorption, and reduced absorption of tryptophan and other amino acids needed for glucose homeostasis [PMID: 32669391 TE142]. Lipid rafts are associated with SARS-CoV-2 virulence and may correlate with different severity profiles in bacterial (*E. coli*), mycobacteria, and viral infections (HIV and coronaviruses) [PMID: 32833058 TE143]. Comparing patients recovered from SARS with healthy volunteers it was shown: a comprehensive elevation of LPIs and PIs; elevation of lactic acid derived from glycolysis, cysteine, aspartic acid, and alanine, increase of the ratio of carnitine to free carnitine, suggesting that fatty acids could easily enter the mitochondria for further β-oxidation in the recovered SARS patients [PMID: 28831119 TE144]. Similar findings have been also observed during SARS-CoV-2 infection: metabolic alterations remarkably differed from COVID-19 patients and healthy controls, and among different COVID-19 severity profiles. Pathway analysis revealed that these alterations mainly involved the metabolic pathways of lysine degradation, and metabolism of taurine, hypotaurine, alphalinolenic acid, glycerophospholipid, arginine, and proline, as well as arginine biosynthesis [PMID: 33596592 TE145]. Particularly, critical patients are characterised by triglycerides upregulation; dysregulation of porphyrin metabolism; activation of gluconeogenesis; abundance of oleic acid and arachidonic acid; glycerophosphocholine and sphingomyelins downregulation, increased 21-hydroxypregnenolone level (essential for corticosterone synthesis); activation of kynurenine pathway; downregulation of choline and its derivatives; accumulation of mannose and its derivatives. Non-critical patients are characterized by strong alteration of lipids, including acylcarnitines, diacylglycerols, fatty acids, glycerophosphoethanolamines, glycerophosphocholines, sphingomyelins, and triacylglycerols; perturbation of phenylalanine, tyrosine and tryptophan biosynthesis, phenylalanine metabolism, aminoacyl-tRNA degradation, arachidonic acid metabolism, and the tricarboxylic acid cycle. By comparing COVID-19 patients with healthy subjects’ acylcarnitines, diacylglycerols, fatty acids, glycerophosphoethanolamines, glycerophosphocholines, sphingomyelins, and triacylglycerols are the most regulated lipid classes and subclasses. [PMID: 33207699 TE101]

**Tryptophan**

The observed serum accumulation of TG and TG-VLDL in COVID-19 patients may be due to a reduced hepatic capacity to oxidize acetyl-CoA in the mitochondria, which is redirected to the synthesis of acetoacetic acid and 2-hydroxybutyric acid. The serum glucose elevation is also consistent with this model, being mitochondrial oxaloacetate driven to the cytoplasm into gluconeogenesis. The increase in succinic acid, citric acid, glutamic acid, and pyruvic acid, may be related to this dysregulation of hepatic central carbon metabolism [PMID: 33043283 TE100]. Reduced HDL cholesterol, a predominance of LDL particles and elevated triglyceride levels as well as the higher Apolipoprotein B100/Apoliprotein-A1 ratio ratios observed in COVID-19 patients are known features of increased risk of cardiovascular disease and diabetes. Related to these observations, a 12-year follow-up study on patients recovered from the SARS-CoV infection showed that 68% presented hyperlipidemia, 44% cardiovascular abnormalities and 60% glucose metabolism disorders [PMID: 32806897 TE104].

As described above, COVID-19 patients showed also major changes in the seric levels of lipoprotein subclasses and their compositional components [PMID: 32492406 TE94, PMID: 33043283 TE100, PMID: 32610096 TE102]. Soluble low-density lipoprotein receptor (sLDLR), lecithin-cholesterol acyltransferase, and cholesteryl ester transfer protein (CETP) are significantly reduced in mild and severe COVID-19 patients compared with healthy controls. ACO2, IDH, OGDH, DLD, SDH, and MDH in the TCA cycle are lower in COVID-19 patients compared with healthy controls, while Acetyl coenzyme A carboxylase and Fatty acid synthetase are elevated. Significant concurrent elevations in plasma lactate and LDH were observable in COVID-19 patients compared with healthy controls [PMID: 33140861 TE96]. Summarizing, metabolites able to discriminate COVID-19 patients from healthy subjects are: i) lipids quantified in positive mode (phosphatidylcholine, phosphatidylcholine, and phosphatidylethanolamine); ii) lipids quantified in negative mode (arachidonic acid, oleic acid, glycerophosphoethanolamines, and glycerophosphoethanolamines); metabolites (2-hydroxy-3-methylbutyric acid, 2,3,4-trihydroxybutyric acid, 3-hydroxyisovaleric acid, palmitic acid, L-pyroglutammic acid, 2-hydroxybutyric acid, butanedioic acid, galactopyranose, myristic acid, l-valine, and heptanoic acid). [PMID: 33207699 TE101] Metabolic alterations in mild/asymptomatic patients include altered tryptophan metabolism into the kynurenine pathway, widespread dysregulation of nitrogen metabolism, increased markers of oxidant stress, proteolysis, renal dysfunction, and increased circulating levels of glucose and free fatty acids [PMID: 32559180 TE146].

Severity-dependent connections between specific elevated cytokines and downregulation of certain classes of metabolites and metabolic processes, suggest an orchestration between increasing disease severity, elevated inflammation, and loss of key circulating nutrients [PMID: 33171100 TE90 ]. Pathways involved comorbidities associated and related to COVID-19 are listed in Table S4 E.

## **11. Glossary**

| **Glossary** |  |
| --- | --- |
| A1BG | Alpha-1-B Glycoprotein |
| A2M | Alpha-2-Macroglobulin |
| ACTB | Actin Beta |
| ACTG1 | Actin Gamma 1 |
| ALB | Albumin |
| ALDOA | Aldolase A, fructose-bisphosphate |
| APOA1 | Apolipoprotein A1 |
| APOA2 | Apolipoprotein A2 |
| APOC1 | Apolipoprotein C1 |
| APOD | Apolipoprotein D |
| APOH | Apolipoprotein H |
| APOL1 | Apolipoprotein L1 |
| APOL6 | Apolipoprotein L6 |
| APOM | Apolipoprotein M |
| APP | Acute Phase Protein |
| ARG1 | Arginase 1 |
| ASC | Antibody-Secreting Cells |
| AZGP1 | Zinc-alpha-2-glycoprotein 1 |
| AZU1 | Azurocidin 1 |
| BATF | Basic Leucine Zipper ATF-Like Transcription Factor |
| BCL11B | B cell leukemia 11B |
| BPI | Bactericidal/permeability-increasing protein |
| BTN3A1 | Butyrophilin Subfamily 3 Member A1 |
| C1R | Complement receptor type 1 |
| C1S | Complement component 1S |
| C4B | Complement component 4B |
| C4BPB | Complement component 4 Binding Protein Beta |
| C6 | Complement component 6 |
| C7 | Complement component 7 |
| C8A | Complement component 8 Alpha Chain |
| C8G | Complement C8 Gamma Chain |
| C9 | Complement component 9 |
| CAMK4 | Calcium/Calmodulin Dependent Protein Kinase IV |
| CARD11 | Caspase Recruitment Domain Family Member 11 |
| CASP8 | Caspase-8 |
| CCDC88A | Coiled-Coil Domain Containing 88A |
| CCL2 | C-C Motif Chemokine Ligand 2 |
| CCL27 | C-C Motif Chemokine Ligand 27 |
| CCL7 | C-C Motif Chemokine Ligand 7 |
| CCL8 | C-C Motif Chemokine Ligand 8 |
| CCR6 | C-C Motif Chemokine Receptor 6 |
| CCR7 | C-C Motif Chemokine Receptor 7 |
| CD109 | Cluster of Differentiation 109 |
| CD14 | Cluster of Differentiation 14 |
| CD16 | Cluster of Differentiation 16 |
| CD163 | Cluster of Differentiation 163 |
| CD177 | Cluster of Differentiation 177 |
| CD19 | Cluster of Differentiation 19 |
| CD197 | Cluster of Differentiation 197 |
| CD2 | Cluster of Differentiation 2 |
| CD226 | Cluster of Differentiation 226 |
| CD24 | Cluster of Differentiation 24 |
| CD244 | Cluster of Differentiation 244 |
| CD27 | Cluster of Differentiation 27 |
| CD279 | Cluster of Differentiation 279 |
| CD38 | Cluster of Differentiation 38 |
| CD3E | Cluster of Differentiation 3 epsilon |
| CD4 | Cluster of Differentiation 4 |
| CD40 | Cluster of Differentiation 40 |
| CD5 | Cluster of Differentiation 5 |
| CD56 | Cluster of Differentiation 56 |
| CD62L (L-selectin) | Cluster of Differentiation 62 Ligand |
| CD64 | Cluster of Differentiation 64 |
| CD69 | Cluster of Differentiation 69 |
| CD7 | Cluster of Differentiation 7 |
| CD8a | Cluster of Differentiation 8a |
| CETP | Cholesteryl Ester Transfer Protein |
| CFB | Complement Factor B |
| CFH | Complement Factor H |
| CFI | Complement Factor I |
| CFP | Complement factor properdin |
| CLEC3B | C-Type Lectin Domain Family 3 Member B |
| CPN1 | Carboxypeptidase N catalytic chain |
| CRP | C-reactive protein |
| CST3 | Cystatin C |
| CTSG | Cathepsin G |
| CX3CR1 | C-X3-C Motif Chemokine Receptor 1 |
| CXCL1 | C-X-C Motif Chemokine Ligand 1 |
| CXCL10 | C-X-C Motif Chemokine Ligand 10 |
| CXCL11 | C-X-C Motif Chemokine Ligand 11 |
| CXCL16 | C-X-C Motif Chemokine Ligand 16 |
| CXCL17 | C-X-C Motif Chemokine Ligand 17 |
| CXCL2 | C-X-C Motif Chemokine Ligand 2 |
| CXCL6 | C-X-C Motif Chemokine Ligand 6 |
| CXCL8 | C-X-C Motif Chemokine Ligand 8 |
| CXCL9 | C-X-C Motif Chemokine Ligand 9 |
| CXCR1 | C-X-C Motif Chemokine Receptor 1 |
| CXCR2 | C-X-C Motif Chemokine Receptor 2 |
| CXCR5 | C-X-C Motif Chemokine Receptor 5 |
| DC | Dendritic Cells |
| DEFA1 | Defensin Alpha 1 |
| DEFA3 | Defensin Alpha 3 |
| DEFA4 | Defensin Alpha 4 |
| DOCK10 | Dedicator Of Cytokinesis 10 |
| DOCK2 | Dedicator Of Cytokinesis 2 |
| ELANE | Elastase, Neutrophil Expressed |
| EP300 | E1A Binding Protein P300 |
| F12 | Coagulation Factor 12 |
| F13A1 | Coagulation Factor XIII A Chain |
| F13B | Coagulation Factor XIII B Chain |
| F2 | Coagulation Factor 2 (prothrombin) |
| F9 | Coagulation Factor 9 |
| FADD | Fas Associated Via Death Domain |
| FCER2 | Fc Fragment Of IgE Receptor II |
| FCGR3A | Fc Fragment Of IgG Receptor III-A |
| FCN1 | Ficolin 1 |
| FETUB | Fetuin B |
| FGA | Fibrinogen alpha |
| FGB | Fibrinogen beta |
| FGG | Fibrinogen gamma |
| FN1 | Fibronectin 1 |
| FYN | FYN Proto-Oncogene, Src Family Tyrosine Kinase |
| GLYC A | α-1-acid glycoprotein A |
| GLYC B | α-1-acid glycoprotein B |
| GNLY | Granulysin |
| GPLD1 | Glycosylphosphatidylinositol Specific Phospholipase D1 |
| GSN | Gelsolin |
| GZMB | Granzyme B |
| GZMH | Granzyme H |
| HD | Healthy Donor |
| HDL | High-Density Lipoprotein |
| HGF | Hepatocyte Growth Factor |
| HLA-DRA | Major Histocompatibility Complex, Class II, DR Alpha |
| HP | High Phosphate |
| HRG | Histidine-Rich Glycoprotein |
| IAV | Influenza A Virus |
| ICU | Intensive Care unit |
| IDL | Intermediate Density Lipoproteins |
| IDO | Indole 2,3-dioxygenase |
| IFI27 | Interferon alpha-inducible protein 27 |
| IFI44L | Interferon Induced Protein 44 Like |
| IFI6 | Interferon alpha-inducible protein 6 |
| IFIH1 | Interferon Induced with Helicase C Domain 1 |
| IFIT2 | Interferon Induced Protein With Tetratricopeptide Repeats 2 |
| IFIT3 | Interferon Induced Protein With Tetratricopeptide Repeats 3 |
| IFITM | Interferon‐Induced Transmembrane Proteins |
| IFN | Interferon |
| IFNα | Interferon-α |
| IFNβ | Interferon-β |
| IFNγ | Interferon-γ |
| IgA | Immunoglobulin A |
| IgG | Immunoglobulin G |
| IGHA1 | Immunoglobulin Heavy Constant Alpha 1 |
| IGHG1 | Immunoglobulin Heavy Constant Gamma 1 |
| IGHV2-5 | Immunoglobulin Heavy Variable 2-5 |
| IGHV3-30 | Immunoglobulin Heavy Variable 3-30 |
| IGLV3-19 | Immunoglobulin Lambda Variable 3-19 |
| IGLV3-25 | Immunoglobulin Lambda Variable 3-25 |
| IL-1 | Interleukin-1 |
| IL-10 | Interleukin-10 |
| IL-12 | Interleukin-12 |
| IL-13 | Interleukin-13 |
| IL-17 | Interleukin-17 |
| IL-17A | Interleukin-17A |
| IL-17F | Interleukin-17F |
| IL-18R | Interleukin-18 Receptor |
| IL-1Ra | Interleukin-1 Receptor Antagonist |
| IL-1β | Interleukin-1β |
| IL-20 | Interleukin-20 |
| IL-21 | Interleukin-21 |
| IL-6 | Interleukin-6 |
| IL-7R | Interleukin-7 Receptor |
| IL-8 | Interleukin-8 |
| iNOS | Inducible nitric oxide synthase |
| IRF1 | Interferon Regulator Factor 1 |
| IRF4 | Interferon Regulator Factor 4 |
| ISG | Interferon Stimulated Gene |
| ISG15 | Interferon Stimulated Gene 15 |
| ITIH3 | Inter-alpha-trypsin inhibitor heavy chain 3 |
| ITIH4 | Inter-alpha-trypsin inhibitor heavy chain 4 |
| JAK | Janus Kinase |
| KLKB1 | Prekallikrein B1 |
| KLRD1 | Killer Cell Lectin Like Receptor D1 |
| KNG1 | Kininogen-1 |
| LAG3 | Lymphocyte Activation Gene 3 |
| LAMP3 | Lysosomal Associated Membrane Protein 3 |
| LBP | Lipopolysaccharide-binding protein |
| LCK | Lymphocyte-specific protein tyrosine kinase |
| LCN2 | Lipocalin-2 |
| LCP1 /LPL | Lymphocyte Cytosolic Protein 1 |
| LDL | Low-Density Lipoproteins |
| LDN | Low Density Neutrophils |
| LEF1 | Lymphoid Enhancer Binding Factor 1 |
| LGALS3BP | Lectin galactoside-binding soluble 3 binding protein |
| LRG1 | Leucine-Rich alpha2 Glycoprotein 1 |
| LTF | Lactotransferrin |
| LY6E | Lymphocyte Antigen 6 Family Member E |
| LYZC | Lysozyme C |
| MAPK | Mitogen-Activated Protein Kinase |
| mDC | Myeloid Dendritic Cells |
| MKI67 | Marker Of Proliferation Ki-67 |
| MMP8 | Matrix metalloproteinase-8 |
| MPO | Myeloperoxidase |
| MX1 |  |
| NCKAP1L | NCK Associated Protein 1 Like |
| NETs | Neutrophil Extracellular Traps |
| NK | Natural Killer |
| NLRC3 | NOD-like receptor family CARD domain containing 3 |
| NR3C1 | Nuclear Receptor Subfamily 3 Group C Member 1 |
| OAS2 | 2'-5'-Oligoadenylate Synthetase 2 |
| OASL | 2'-5'-Oligoadenylate Synthetase Like |
| OLAH | Oleoyl-ACP hydrolase |
| OLFM4 | Olfactomedin-4 |
| ORM1 | Orosomucoid 1/alpha-1-acid glycoprotein-1 |
| ORM2 | Orosomucoid- 1/alpha-1-acid glycoprotein-2 |
| OSM | Oncostatin-M |
| PBMC | Peripheral Blood Mononuclear Cell |
| PBs | Plasmablasts |
| PD1 | Programmed Cell Death Protein 1 |
| pDC | Plasmacytoid dendritic cells |
| PD-L1 | Programmed Death-Ligand 1 |
| PF4 | Platelet factor 4 |
| PI16 | Peptidase Inhibitor 16 |
| PLA2 | Phospholipase A2 |
| PLAC8 | Placenta-specific 8 |
| PLG | Plasminogen |
| PPAR complex | Peroxisome proliferator-activated receptors |
| PPBP | Pro-Platelet Basic Protein |
| PRDM1 | PR domain zinc finger protein 1 |
| PRF1 | Perforin 1 |
| PROC | Protein C |
| PROS | Antithrombotic Protein S |
| PROS1 | Vitamin K dependent protein S |
| PRTN3 | Proteinase 3 |
| RARA | Retinoic Acid Receptor Alpha |
| RIG-I | Retinoic acid-inducible gene I |
| RIPK3 | Receptor Interacting Serine/Threonine Kinase 3 |
| RSAD2 | Radical S-Adenosyl Methionine Domain Containing 2 |
| RXRA | Retinoid X Receptor Alpha |
| S100 A11 | S100 calcium-binding protein A11 |
| S100 A12 | S100 calcium-binding protein A12 |
| S100 A4 | S100 calcium-binding protein A4 |
| S100 A8 | S100 calcium-binding protein A8 |
| S100 A9 | S100 calcium-binding protein A9 |
| S100 P | S100 calcium-binding protein P |
| SAA1 | Serum amyloid A-1 |
| SAA2 | Serum amyloid A-2 |
| SAA4 | Serum amyloid A-4 |
| SAP/APCS | Serum amyloid P-component |
| SARS-CoV-2 | Severe Acute Respiratory Syndrome COronaVirus 2 |
| SERPIN A10 | Serine Protease Inhibitor Family A Member 10 |
| SERPIN A3 / ACT | Serine Protease Inhibitor Family A Member 3/ Alpha-1-antichymotrypsin |
| SERPIN A4 | Serine Protease Inhibitor Family A Member 4 |
| SERPIN A5 | Serine Protease Inhibitor Family A Member 5 |
| SERPIN D1 | Serine Protease Inhibitor Family D Member 1 |
| SERPIN F2 | Serine Protease Inhibitor Family F Member 2 |
| SERPINE1 | Serine Protease Inhibitor Family E Member 1 |
| SIGLEC5 | Sialic acid-binding immunoglobulin-like lectin 5 |
| SKAP1 | Src Kinase Associated Phosphoprotein 1 |
| SLAMF7 | Signaling lymphocytic activation molecule F7 |
| SLC9A3R1 | Solute Carrier family 9 (sodium/hydrogen exchanger) member 3 regulator 1 |
| SOCS3 | Suppressor Of Cytokine Signaling 3 |
| SPN | Sialophorin |
| SPP2 | Secreted Phosphoprotein 2 |
| SRGN | Serglycin |
| STAT1 | Signal Transducer and Activator of Transcription 1 |
| STAT3 | Signal Transducer and Activator of Transcription 3 |
| SUMO1 | Small Ubiquitin Like Modifier 1 |
| TAFI | Thrombin-Activatable Fibrinolysis Inhibitor |
| TAGLN2 | Transgelin 2 |
| TC | Total Cholesterol |
| TCA | Tricarboxylic acid cycle |
| TCF7 | Transcription Factor 7 |
| TCR | T Cell Receptor |
| TF | Transferrin |
| TG | Triglyceride |
| TGFB1 | Transforming Growth Factor Beta 1 |
| Th1 | T helper 1 |
| Th17 | T helper 17 |
| Th2 | T helper 2 |
| THBS1 | Thrombospondin 1 |
| TIGIT | T cell immunoglobulin and ITIM domain |
| TIMP1 | Tissue inhibitor of metalloproteinases 1 |
| TLN1 | Talin 1 |
| TLR | Toll-like receptor |
| TNF | Tumor Necrosis Factor |
| TNFRSF10A | TNF Receptor Superfamily Member 10A |
| TNFRSF1B | TNF Receptor Superfamily Member 1B |
| TNFSF14 | Tumor Necrosis Factor Superfamily Member 14 |
| TRADD | Tumor necrosis factor receptor type 1-associated DEATH domain |
| TRAF | TNF Receptor Associated factor |
| TRAIL | Tumor necrosis factor-related apoptosis-inducing ligand |
| TRIM59 | Tripartite Motif Containing 59 |
| TYMS | Thymidylate synthase |
| USP21 | Ubiquitin Specific Peptidase 21 |
| VLDL | Very low-density lipoproteins |
| VTN | Vitronectin |
| VWF | Von Willebrand Factor |
| XAF1 | X-linked inhibitor of apoptosis (XIAP)-associated factor 1 |
| XBP1 | X-box binding protein 1 |
